# Variable responses of human and non-human primate gut microbiomes to a Western diet

**DOI:** 10.1186/s40168-015-0120-7

**Published:** 2015-11-16

**Authors:** Katherine R. Amato, Carl J. Yeoman, Gabriela Cerda, Christopher A. Schmitt, Jennifer Danzy Cramer, Margret E. Berg Miller, Andres Gomez, Trudy R. Turner, Brenda A. Wilson, Rebecca M. Stumpf, Karen E. Nelson, Bryan A. White, Rob Knight, Steven R. Leigh

**Affiliations:** Department of Anthropology, Northwestern University, Evanston, USA; Department of Anthropology, University of Colorado Boulder, Boulder, USA; BioFrontiers Institute, University of Colorado Boulder, Boulder, USA; Department of Range Sciences, Montana State University, Bozeman, USA; Department of Anthropology, University of Illinois, Urbana, USA; Department of Anthropology, Boston University, Boston, USA; Department of Sociology, Anthropology, and Women’s Studies, American Military University and American Public University, Charles Town, USA; The Institute for Genomic Biology, University of Illinois, Urbana, IL 61801 USA; Department of Ecology, Evolution, and Behavior, University of Minnesota, Minneapolis, USA; Department of Anthropology, University of Wisconsin, Milwaukee, USA; Department of Genetics, University of the Free State, Bloemfontein, South Africa; Department of Microbiology, University of Illinois, Urbana, USA; The J. Craig Venter Institute, Rockville, USA; Department of Animal Sciences, University of Illinois, Urbana, USA; School of Medicine, University of California San Diego, La Jolla, USA; Center for Neurobehavioral Genetics, University of California, Los Angeles, CA USA

**Keywords:** Gut microbiome, Vervet, *Chlorocebus aethiops*, Western diet, Human evolution

## Abstract

**Background:**

The human gut microbiota interacts closely with human diet and physiology. To better understand the mechanisms behind this relationship, gut microbiome research relies on complementing human studies with manipulations of animal models, including non-human primates. However, due to unique aspects of human diet and physiology, it is likely that host-gut microbe interactions operate differently in humans and non-human primates.

**Results:**

Here, we show that the human microbiome reacts differently to a high-protein, high-fat Western diet than that of a model primate, the African green monkey, or vervet (*Chlorocebus aethiops sabaeus*). Specifically, humans exhibit increased relative abundance of Firmicutes and reduced relative abundance of *Prevotella* on a Western diet while vervets show the opposite pattern. Predictive metagenomics demonstrate an increased relative abundance of genes associated with carbohydrate metabolism in the microbiome of only humans consuming a Western diet.

**Conclusions:**

These results suggest that the human gut microbiota has unique properties that are a result of changes in human diet and physiology across evolution or that may have contributed to the evolution of human physiology. Therefore, the role of animal models for understanding the relationship between the human gut microbiota and host metabolism must be re-focused.

**Electronic supplementary material:**

The online version of this article (doi:10.1186/s40168-015-0120-7) contains supplementary material, which is available to authorized users.

## Background

A common theme in human gut microbiome research currently is the effect of diet on gut microbiota composition, and ultimately, host physiology [1]. In particular, a number of studies focus on the impact of a “Western” diet (high in animal fat and protein and low in fiber) compared to a “non-Western” diet (low in animal fat and protein and high in fiber; [2–6]). Western and non-Western human diets are consistently associated with distinct gut microbial communities [2–6], and humans consuming a high-protein, high-fat Western diet also have higher rates of obesity and other metabolic syndromes such as diabetes [7]. Gut microbes appear to be implicated in many of these conditions [8–10], indicating a link between the human gut microbiota, diet, and physiology.

As data describing patterns in the human gut microbiota accumulate, researchers are beginning to turn toward animal models to test the mechanisms driving host-gut microbe interactions and their physiological consequences. While the use of rodent models is particularly popular due to ease of manipulation, non-human primate models are being integrated as well as a result of their close phylogenetic relationships with humans and presumably similar physiology [1, 11–13]. For example, a recent study examined the impact of a high-fat diet on the gut microbiota of adult female macaques and their offspring [12].

However, human evolution appears to reflect a major dietary shift from a predominantly plant-based diet to an increasingly carnivorous diet, with relatively recent increases in food digestibility due to tool use, cooking, and other processing techniques [14–16]. Additionally, compared to non-human primates, humans are characterized by a number of unique physiological adaptations, including increased brain size, reduced gut size, increased fat deposition, and decreased muscle mass [17–19]. Differences also exist between human and non-human primate Toll-like receptors in the gut as well as expression of enzymes for the production of hormones such as prostaglandin [20–22]. Because diet can alter the taxonomic composition of the human gut microbiota during an individual’s lifetime [23, 24], and human metabolism and physiology is closely linked to the gut microbiota [25], it is likely that evolutionary shifts in human diet and physiology were accompanied by shifts in the gut microbiota. As a result, the human gut microbiota should exhibit unique properties when compared to the non-human primate gut microbiota. If this is the case, non-human primate models of host-gut microbe relationships may be less ideal than assumed for addressing questions regarding human diet and physiology in the context of the gut microbiota.

Recent data provide evidence that the human gut microbiota is less diverse and better adapted to a meat-rich diet compared to closely related extant primates [26], but no direct comparison of host-gut microbe dynamics in humans and non-human primates currently exists. Therefore, it is unclear if the relationship between the gut microbiota and host diet and physiology differs in humans compared to other primates. To improve our understanding of how host-gut microbe interactions are similar or different in human and non-human primates, here, we use 454 titanium pyrosequencing and predictive metagenomics (PICRUSt) [27] to compare the gut microbiota of humans and African green monkeys, or vervets (*Chlorocebus aethiops sabaeus*), consuming both a Western and a non-Western diet. Physiological similarities to humans make vervets, an attractive model for biomedical trials [28], particularly with regards to human metabolic disorders and obesity [29, 30]. Vervets may therefore provide an excellent reference system for understanding how unique the responses of the human gut microbiota to diet are compared to other primates.

Despite the utility of vervets as models for human physiology in many contexts, we hypothesized that the vervet gut microbiota would exhibit distinct responses to a Western diet when compared with the human gut microbiota. Specifically, we predicted that the relative abundances of fewer microbial taxa and genes would differ in response to diet in the vervet gut microbiota since vervets have not evolved to include large amounts of animal fat and protein in their natural diets [31]. Consequently, the vervet gut microbiota should be less able to adapt to this type of Western diet and may not be an appropriate model for understanding human gut microbe interactions in the context of diet.

## Results

### Vervets

**Fig. 1:**
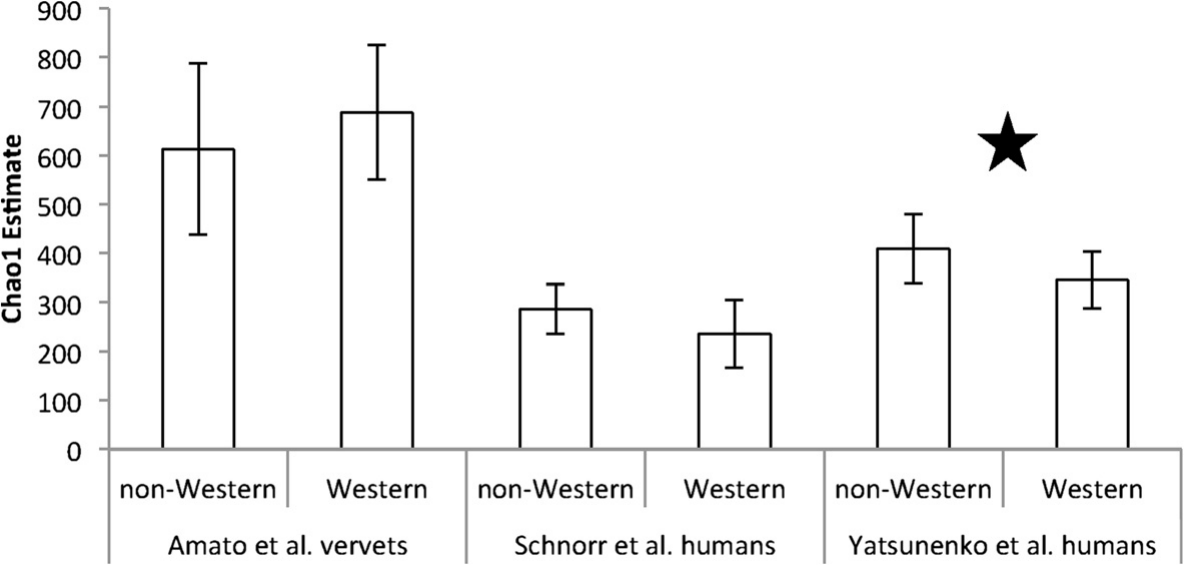
A Western diet reduces gut microbial diversity in humans but not vervets. Chao1 estimates (average ± SD) of microbial community richness at 1000 sequence reads per sample for vervets and humans consuming a non-Western vs. a Western diet. Star indicates significant differences (FDR-corrected *p* < 0.05) in microbial richness between diets. Western humans are from Italy (Schnorr et al. [3]) and the USA (Yatsunenko et al. [2]). Non-western humans are from Tanzania (Hadza, Schnorr et al. [3]), Venezuela (Guahibo, Yatsunenko et al. [2]), and Malawi (Yatsunenko et al. [2]). (*N* = 13 non-Western vervets; *N* = 14 Western vervets; *N* = 17 non-Western (Schnorr et al.) humans; *N* = 11 Western (Schnorr et al.) humans; *N* = 76 non-Western (Yatsunenko et al.) humans; *N* = 118 Western (Yatsunenko et al.) humans)

**Fig. 2:**
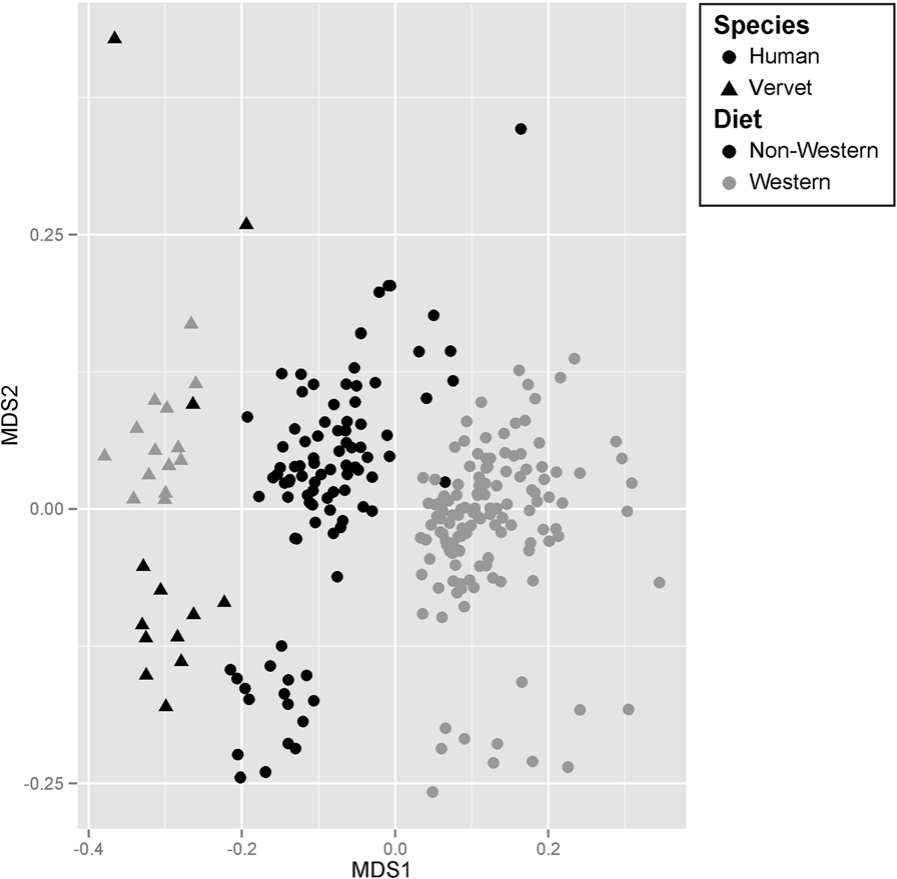
The vervet gut microbiome resembles a non-Western human gut microbiome regardless of the vervet diet. Non-metric multi-dimensional scaling (NMDS) plot based on unweighted UniFrac distances illustrating clustering patterns in gut microbiomes across sampling groups at the OTU level

Results show that although vervets fed a typical Western diet (TWD, see Methods for composition) had similar gut microbial richness compared to wild vervets consuming a non-Western diet (Fig. 1), the composition of their gut microbiota was distinct (unweighted UniFrac distance, *F*_1,26_ = 4.8, *p* < 0.001, *r*^2^ = 0.16, Fig. 2; weighted UniFrac distance, *F*_1,26_ = 9.6, *p* < 0.001, *r*^2^ = 0.28, Additional file 1: Figure S1, Table S1-S5). Specifically, at the phylum level, TWD-fed vervets had significantly lower relative abundances of Firmicutes, Lentisphaerae, Proteobacteria, Tenericutes, and Verrucomicrobia than wild vervets (Additional file 1: Table S1). In contrast, TWD-fed vervets had significantly higher abundances of Bacteroidetes and TM7, with a trend for higher relative abundances of Spirochaetes (Additional file 1: Table S1). At the genus level, we also detected lower relative abundances of *Clostridium* and a tendency for lower relative abundances of *Bifidobacterium* in TWD-fed vervets as well as higher relative abundances of *Desulfovibrio*, *Prevotella, Cantenibacterium*, and *Collinsella* (Additional file 1: Table S5).

Predictive metagenomics also revealed differences in the vervet gut microbiome in response to diet (*F*_1,57_ = 4.94, *p* = 0.01, *r*^2^ = 0.08). Specifically, 22 genes differed in relative abundance between wild vervets and TWD-fed vervets. For example, K00432, a glutathione peroxidase, which is associated with lipid and amino acid metabolism, was present at higher relative abundances in vervets consuming a TWD than wild vervets (Additional file 1: Table S6). The same pattern was observed in the relative abundance of K02619, a 4-amino-4-deoxychorismate lyase, which is associated with folate biosynthesis. In contrast, K00068 (sorbitol-6-phosphate 2-dehydrogenase), K00844 (hexokinase), and K05884 (L-2-hydroxycarboxylate dehydrogenase), with roles in fructose and mannose metabolism, carbohydrate and lipid metabolism, and coenzyme M biosynthesis, respectively, were all predicted in lower relative abundances in TWD-fed vervets (Additional file 1: Table S6). At the pathway level, TWD-fed vervets exhibited increased relative abundances of genes associated with amino acid metabolism (Additional file 1: Table S7).

### Humans vs. vervets

Both human studies revealed differences in the gut microbiota associated with diet both in terms of microbial taxonomic composition (unweighted UniFrac distance, Hadza/Italy, *F*_1,27_ = 4.9, *p* < 0.001, *r*^2^ = 0.16, Malawi/Venezuela/U.S., *F*_1,193_ = 31.6, *p* < 0.001, *r*^2^ = 0.14, Fig. 2; weighted UniFrac distance, Hadza/Italy, *F*_1,27_ = 5.2, *p* = 0.001, *r*^2^ = 0.17; Malawi/Venezuela/USA, *F*_1,193_ = 72.7, *p* < 0.001, *r*^2^ = 0.27 Additional file 1: Figure S1, Additional file 1: Table S1-S5) and gene relative abundances (Hadza/Italy, *F*_1,42_ = 6.1, *p* = 0.003, *r*^2^ = 0.13; Malawi/Venezuela/USA, *F*_1,193_ = 61.3, *p* < 0.001, *r*^2^ = 0.24), but we detected more differences in the relative abundances of microbial taxa between Western and non-Western gut microbiomes in the Malawi/Venezuela/USA dataset, presumably as a result of larger sample sizes (Additional file 1: Tables S1-S7). In addition, several microbial taxa reacted similarly to a Western diet across both human and vervet datasets despite different storage, DNA extraction, and sequencing technologies as well as variation in Western and non-Western diet across countries and species. Like the vervets, Western diet humans from the USA and Italy exhibited lower relative abundances of Proteobacteria, Lentisphaerae, and Tenericutes (*p* = 0.06 for Italians) compared to those on a non-Western diet. Relative abundances of Spirochaetes were also lower in the humans from Malawi and Venezuela compared to the USA, and we detected lower relative abundances of *Clostridium* and higher relative abundances of Desulfovibrionaceae and *Collinsella* in humans from the USA compared to Malawi and Venezuela.

Despite the similarities noted above, we also observed key differences between the gut microbiota of humans and vervets associated with each type of diet. First, examination of the presence or absence of microbial taxa revealed that the vervets clustered more strongly with non-Western humans, especially the Hadza, regardless of whether the vervets were consuming a wild or Western diet (Fig. 2, Additional file 1: Figure S2). Although recent bottlenecks have resulted in low genetic diversity in both vervet populations sampled [28, 32], gut microbiome data from another wild, non-human primate species (*Cercocebus agilis*, Central African Republic) also cluster with the vervets (Additional file 1: Figure S3), indicating that increased genetic diversity among human populations compared to the vervet populations is not driving the observed patterns.

**Fig. 3:**
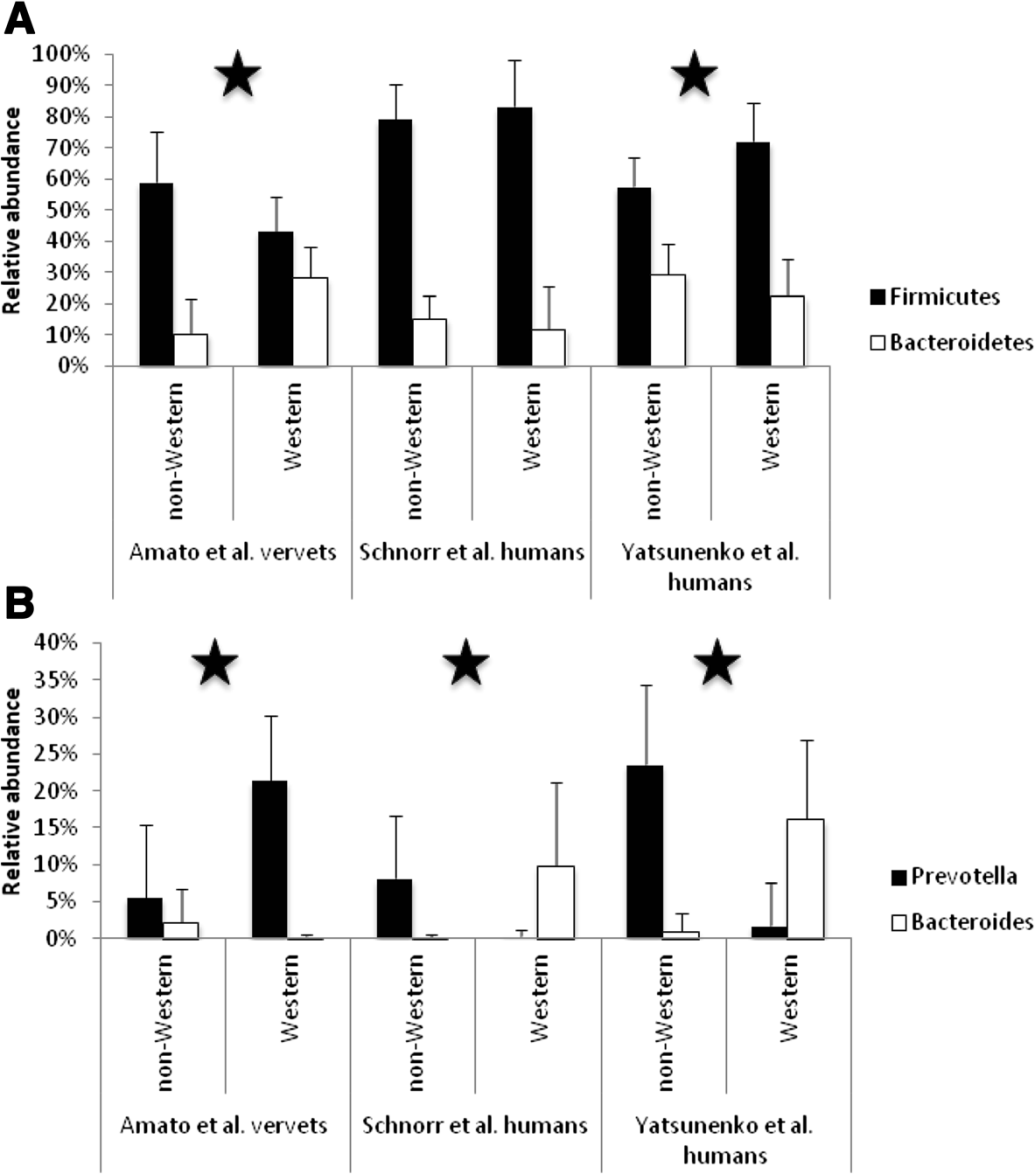
Human and vervet gut microbiomes react differently to a Western diet. Relative abundances of key **a** phyla and **b** genera in humans and vervets consuming non-Western and Western diets. Western humans are from Italy (Schnorr et al. [3]) and the USA (Yatsunenko et al. [2]). Non-Western humans are from Tanzania (Hadza, Schnorr et al. [3]), Venezuela (Guahibo, Yatsunenko et al. [2]), and Malawi (Yatsunenko et al. [2]). Stars indicate significant differences (FDR-corrected p < 0.05) in relative abundances between diets for both humans and vervets. However, *Bacteroides* relative abundances were not significantly different between diets for vervets

In addition, Chao1 species diversity estimates tended to be lower in humans consuming a Western diet vs. a non-Western diet (Fig. 1), while diversity in vervets was unaffected by diet. Compared to humans, vervets also exhibited distinct changes in the relative abundances of microbial taxa in response to a Western diet. While people in the USA exhibited increased relative abundances of Firmicutes and reduced relative abundances of Bacteroidetes compared to people in Venezuela and Malawi, vervets exhibited the following opposite pattern: a reduction in Firmicutes relative abundances and an increase in Bacteroidetes relative abundances in response to a Western diet (Fig. 3a). Furthermore, Western human populations exhibited lower relative abundances of *Prevotella* and higher relative abundances of *Bacteroides* and *Bifidobacterium*, which contrasts sharply with the patterns observed in the vervets (Fig. 3b). Vervets also exhibited increased levels of *Cantenibacterium* on a Western diet, but both human populations we examined showed the opposite pattern. Finally, in both human populations, relative abundances of *Succinovibrio* and *Treponema* were lower for people consuming a Western diet, but diet did not signficantly affect these taxa in vervets.

Predictive metagenomic data also showed strong differences in the reactions of the vervet and human gut microbiota to diet. Although the relative abundance of K00432 (glutathione peroxidase) changed in response to diet in both humans and vervets, its relative abundance decreased in humans on a Western diet while in vervets its relative abundance increased (Additional file 1: Table S6). The same was true of K01858 (myo-inositol-1-phosphate synthase), K02548 (general secretion pathway protein I), and K02619 (4-amino-4-deoxychorismate lyase), associated with carbohydrate metabolism, cofactor and vitamin metabolism/biosynthesis, and folate biosynthesis, respectively (Additional file 1: Table S6). Additionally, vervets showed shifts in the relative abundances of eight genes that did not shift in response to diet in humans in either study. At the pathway level, humans also showed an increased relative abundance of genes associated with carbohydrate metabolism when consuming a Western diet, a difference that was not observed in vervets (Additional file 1: Table S7).

## Discussion

As hypothesized, our data indicate that the composition of the human and vervet gut microbiota is distinct on a Western diet. However, we did not observe differences in the relative abundance of fewer microbial taxa and genes in the vervets compared to the humans. Instead, the effect of a Western diet on the vervet gut microbiota was driven by distinct patterns in microbial taxa and genes compared to humans.

Many of the patterns we detected in both vervets and humans have been reported in other studies. For example, we observed elevated microbial richness and higher relative abundances of *Prevotella* in non-Western humans and elevated relative abundances of *Bacteroides* in Western humans. These results are concordant with a study of the human gut microbiota that associates diets high in protein and animal fat with high levels of *Bacteroides* and diets high in plant carbohydrates with high levels of *Prevotella* [23, 33]. Similarly, all published studies of Western and non-Western humans to-date report higher microbial richness and higher relative abundances of *Prevotella* in non-Western populations [2–6, 34]. In contrast, vervets on a Western diet showed similar microbial richness and higher relative abundances of *Prevotella*, a change that is mirrored in the macaque model [12].

In addition, we detected an elevated relative abundance of *Collinsella* in humans on a Western diet, and *Collinsella* has been associated with obesity in other studies of humans [35]. Similarly, while decreased relative abundances of Bacteroidetes coupled with increased relative abundances of Firmicutes, like those we observed in Western humans from the USA, are not reported in many studies comparing Western and non-Western humans [4, 5, 34]; they have been associated with obesity in human and mouse studies [36]. In our TWD-fed vervets, we measured the opposite patterns, and these findings are analogous to previous work contrasting a high-fat diet to a captive control (chow) diet in a macaque model (*Macaca fuscata*) where a shift to a high-fat diet also resulted in significant increases in Bacteroidetes, especially *Prevotella* and no significant variations in Firmicutes [12].

These commonalities suggest that our results are not unique to the human and vervet populations that we examined in this study. The human gut microbiota and its response to an easy-to-digest Western diet that is low in fiber differ fundamentally from the non-human primate gut microbiota. These findings provide the first evidence implying a specialization of the human gut microbiota. Therefore, while non-human primates may serve as comparative biomedical models for other aspects of human physiology [28], we suggest that the non-human primate gut microbiota may not provide an ideal direct model for understanding the effect of the human gut microbiota on host metabolism and nutrition in the context of a Western diet.

It is important to note that both the human and vervet data used in this study compare distinct populations, and therefore it is impossible to control for potential non-diet influences on the gut microbiota such as host genetics, exposure to local microbial pools, and antibiotic use. Likewise, future studies must control for the potential effects of non-human primate captivity such as host social contact networks and early life influences on the gut microbiota. However, it is unlikely that the results we present here are primarily driven by these potential confounds. Patterns in confounding factors such as antibiotic use across populations of the same host are likely to be similar for both humans and vervets, with Western humans and captive vervets generally having more exposure to antibiotics than non-Western humans and wild vervets. Therefore, while diet may not be the only factor causing differences between populations of the same host species, the comparisons of patterns between the two host species remains valid. Additionally, the captive population of vervets was taken from St. Kitts between 1975 and 1980. As a result, the two populations are only separated by 3–8 generations, reducing the impact of host genetics as a potential confound for the vervets.

Despite the comparison of multiple datasets in this analysis, studies generally indicate that host diet has the strongest effect on the gut microbiota compared to other factors [37], reducing the potential for confounding factors associated with different host populations to drive the observed patterns. For example, when human studies broadly control for host ethnic backgrounds, they continue to illustrate strong impacts of Western and non-Western diets, suggesting that differences in gut microbiota composition between populations of the same host species with distinct diets are unlikely to be genetically-driven [6]. Furthermore, although the vervet Western diet is distinct from a human Western diet, it is important to note that Western and non-Western human diets vary markedly across populations (e.g., USA vs. Italy and Malawi vs. Venezuela vs. Tanzania; [2, 3]). Despite differences within these diet categories, Western and non-Western human populations still cluster together in terms of gut microbiota composition, and other studies comparing Western and non-Western humans consistently report similar patterns [2–6, 34], suggesting that differences in the human and vervet gut microbiota are not driven by subtle variation in the composition of either a Western or non-Western diet or by idiosyncrasies in the selected datasets. Likewise, while the data we present were not all generated using the same methodology, the potential effects of distinct DNA extraction protocols, PCR primers, or sequencing platforms on gut microbiota data have been shown to be small [38, 39] compared to the effects of diet. Additionally, all DNA extractions utilized bead-beating step, which reduces extraction bias [40], and our use of the same closed-reference OTU picking pipeline (see methods) on rarefied data also reduces the potential effect of sequencing error and read depth on the results [41]. Therefore, while we cannot completely eliminate the biases of study-specific methodology on this dataset, we are confident that the patterns presented are biologically-driven.

Although data for other non-human primate taxa must be collected, we propose two explanations for the distinct responses of the human and non-human primate gut microbiota to diet. First, it is possible that the unique properties observed in the human gut microbiota are simply the result of unique human diet and physiology (namely an easy-to-digest, meat-heavy diet and increased brain size, reduced gut size, increased fat deposition, and decreased muscle mass [17–19]). Since both host diet and physiology drive gut microbiota composition [42], evolutionary changes in human diet and physiology could have easily led to a distinct gut microbiota. Another possible explanation is that, in addition to other factors such as diet, unique properties of the human gut microbiota contributed to the evolution of human physiology. For example, if over time the human gut microbiota shifted in a way that confers an increased capacity for energy production and storage, it could have promoted increased brain size during human evolution. Similarly, because the human gut microbiota plays a role in regulating host energy intake and fat production [43], and an increased capacity to store energy as fat has been hypothesized to have enabled humans to develop larger brains [18, 19], changes in the gut microbiota that affected host metabolic pathways could have contributed to the evolution of the human brain as well. To distinguish between these two alternatives, further studies are necessary that measure the metabolic potential of the human and non-human gut microbiota and more directly compare the physiological consequences of consuming a Western diet. However, a recent study suggests that large-brained primates endure seasonal periods of food limitation more successfully than small-brained primates [44]. Together with evidence that gut microbes compensate for periodically reduced energy intake in some wild primates [45], these data could indicate a role for the gut microbiota in buffering hosts nutritionally against energetically expensive physiological adaptations.

Finally, our data indicated that, in addition to the gut microbiota, the physiological responses of humans and non-human primates to a Western diet may be distinct. Although vervets have an adverse metabolic reaction to a Western diet and can become obese in captivity [46, 47], most of the TWD-fed vervets did not gain weight during the study (Additional file 1: Table S8). It is possible that two factors restricted vervet weight gain: (1) finite food availability and (2) study duration (6 months). We regard these factors as unlikely to be limiting because the vervets were provided with enough food to result in a 10 % daily surplus, and no obvious reduction in food intake was observed [47]. Moreover, a sufficiently high-protein, high-fat diet can impact the gut microbiota almost immediately in humans, and changes are similar to those observed across populations with distinct diets [24], suggesting that a 6-month interval was sufficient. Although the captive vervets weighed more than the wild vervets both before and after the diet challenge, indicating an effect of captivity on body weight, this effect is likely a result of increased food availability and decreased activity, both of which would be expected to exacerbate weight gain and negative health outcomes on a Western diet, not mitigate them. While additional research is necessary to confirm and examine this pattern in more detail, we suggest that a side effect of the proposed unique human gut microbiota may be an increased susceptibility to obesity and metabolic disorders, particularly when hosts are consuming a high-protein, high-fat diet. If the non-human primate gut microbiota does, in fact, possess properties that make it resistant to obesity when subjected to a Western diet, it may open new avenues of exploration for translational metabolic therapies. What might confer this resistance and whether it can be maintained over generations of Western diet consumption remains to be investigated, but understanding these factors could help develop treatments to improve human resistance to obesity via the gut microbiota.

## Conclusions

Although animal models such as non-human primates are commonly used to test mechanisms of human gut microbe interactions, direct comparisons of the influence of commonly investigated factors on the gut microbiota of humans and non-human primates are lacking. This study demonstrates that similar host diets differentially affect the human and non-human primate gut microbiota. These results indicate that non-human primates are not appropriate models for directly testing the relationships between human diet, physiology and the gut microbiota. However, the observed patterns have implications for human evolution since they suggest an association between the gut microbiota and unique shifts in human diet and physiology across evolutionary time. A more detailed examination of this association has the potential to transform our understanding of the role of gut microbes in human biology.

## Methods

### Sample collection and processing

Rectal swabs were used to sample the gut microbiota of 27 wild and captive vervets. In January 2010, 15 wild vervet monkeys were captured, sampled, and released on the island of St. Kitts. Wild vervets on St. Kitts are not provisioned and consume mainly ripe fruits, flowers, and seeds [48]. We considered this a non-Western diet. In September 2009, 23 vervets at the Wake Forest University Primate Center were sampled in a similar manner. These vervets are directly descended from the St. Kitts population (with an initial group obtained in 1975) and were fed a typical Western human diet (TWD; LabDiet 5L0P, Purina, St. Louis, MO; 18 % protein, 37 % fat, 45 % carbohydrates, 9 % fiber) for 6 months before sampling. Sampling of and care for the captive TWD vervets was approved by the Institutional Animal Care and Use Committee at Wake Forest University (IACUC #A10-091). Sampling of the wild monkeys was approved by the University of Illinois Institutional Animal Care and Use Committee (IACUCs #08044, #11046) as well as the University of California Los Angeles IACUC (#2009-053-13). All sampling was carried out in accordance with the approved guidelines at each institution.

All rectal swabs were immediately placed in RNAlater (Qiagen Inc., Valencia, CA, USA) and stored at −80 °C until processing. DNA was extracted from rectal swabs using a MoBio UltraClean Soil DNA Isolation kit (MoBio, Carlsbad, CA, USA) per manufacturer instructions. The V1–V3 region of the bacterial 16S rRNA gene was amplified using pyrotagged primers (MID 1-15) 27f and 534r. Amplicons were checked for specificity with gel electrophoresis and cleaned using the AMPure XP system (Beckman Coulter, Danvers, MA, USA). Amplicons were sequenced using 454 pyrosequencing technology at the J. Craig Venter Institute and/or the University of Illinois KECK Center. No differences were seen in the microbial taxonomic distributions of samples sequenced at both sequencing centers. Therefore, sequencing center was eliminated as a possible effect.

Raw sequence data from non-Western and Western human populations published by Yatsunenko et al. [2] and Schnorr et al. [3] were also obtained for analysis. Non-Western human populations were sampled in Malawi, Venezuela (Guahibo Amerindians), and Tanzania (Hadza). Western human populations were sampled in the USA and Italy. Only individuals between the ages of 18 and 50 were included from the Yatsunenko et al. [2] dataset (Malawi/Venezuela *N* = 76 people, USA *N* = 118 people), while all samples from Schnorr et al. [3] were included (Hadza *N* = 17 people, Italy *N* = 11 people).

### Data analysis

All sequence data were quality filtered so that sequences shorter than 200 nt, longer than 1000 nt, containing incorrect primer sequences, more than six ambiguous base calls and/or homopolymers longer than 7 nt were discarded, resulting in 387,785,923 sequences across all studies. All subsequent data analyses were performed using QIIME (version 1.8.0) [49]. Operational taxonomic units (OTUs) were picked closed-reference against the Green Genes 13_8 database with OTUs defined as sharing ≥97 % identity. This strategy made it possible to compare patterns across studies despite the use of different primers and/or sequencing platforms and reduced the impact of sequencing error on the data. Due to the use of 454 technology in two of the datasets, we rarefied the number of sequences from each sample to 1000 for all studies. Previous studies suggest that this level allows for meaningful comparison [50]. Additionally, nine TWD vervet samples were removed from analysis as a result of unusually high relative abundances of *Brachyspira*. Several *Brachyspira* species are known gut pathogens, and loose stools were observed in some of the enclosures during the diet trial. Removing these samples did not affect the overall patterns observed in beta diversity within and across studies (Additional file 1: Figure S4, S5) but did lead to *Brachyspira* relative abundances in a more typical range.

Chao1 diversity estimates were produced for each sample using QIIME. We also predicted the metagenome associated with each sample using Phylogenetic Investigation of Communities by Reconstruction of Unobserved States (PICRUSt 1.0.0) [27] after normalizing for 16S copy number. The average Nearest Sequenced Taxon Index was 0.11 ± 0.03, with Western humans falling at the lower end of the range and non-Western humans and vervets falling at the higher end.

We tested for significant differences between wild and TWD vervets as well as Western and non-Western humans from each human study. Although inter-study effects on patterns of gut microbial community composition have been demonstrated to be low [38, 39], we tested for the effect of diet within each individual dataset independently to eliminate the potential for confounding factors. Principle coordinate analyses based on unweighted and weighted UniFrac distances were used to visualize differences in gut microbial community composition among populations. PERMANOVA (R software, version 3.0.2, adonis package) was used to test for the effect of diet on microbial community composition and predicted metagenomes for all three datasets. Similarly, for each dataset, we tested for differences in the relative abundances of microbial taxa and genes between wild and TWD vervets or between Western and non-Western humans using a series of Kruskal-Wallis tests at each taxonomic level. *p* values were adjusted using either a family-wide detection rate (microbial taxonomy) or a Bonferroni correction (predicted metagenomes). All statistics were performed using QIIME’s implementation of R.

## Availability of supporting data

The raw sequence files supporting the results of this article are available in the EBI repository, [deposited upon acceptance].

## Additional file

Additional file 1:
**Supplementary tables and figures.** (DOC 2591 KB)
