# Supplementary material for: Variable responses of human and non-human primate gut microbiomes to a Western diet
Source: Microbiome. 2015 Nov 16;3:53. doi: 10.1186/s40168-015-0120-7 (PMC4645477; doi:10.1186/s40168-015-0120-7)
Supplement: Additional file 1: — Supplementary tables and figures. (DOC 2591 KB) [file 40168_2015_120_MOESM1_ESM.doc]

**Variable responses of human and nonhuman primate gut microbiomes to a Western diet**

Katherine R. Amato^1,2*^, Carl J. Yeoman^3^, Gabriella Cerda^4^, Christopher Schmitt^5^, Jennifer Danzy Cramer^6^, Margret E. Berg Miller^7^, Andres Gomez^7,8^, Trudy Turner^9,10^, Brenda A. Wilson^7,11^, Rebecca M. Stumpf^4,7^, Karen E. Nelson^12^, Bryan A. White^6,8^, Rob Knight^2^, Steven R. Leigh^1,7^

Supplementary Tables

Table S1. Average (± SD) relative abundance () of phyla in samples from vervets and humans consuming non-Western and Western diets. Western humans are from Italy (Schnorr et al. 2014) and the U.S. (Yatsunenko et al. 2012). Non-western humans are from Tanzania (Hadza, Schnorr et al. 2014), Venezuela (Guahibo, Yatsunenko et al. 2012), and Malawi (Yatsunenko et al. 2012).

|  | Amato et al. vervets | | | | Schnorr et al. humans | | | | Yatsunenko et al. humans | | | |
| --- | --- | --- | --- | --- | --- | --- | --- | --- | --- | --- | --- | --- |
|  | non-Western | | Western | | non-Western | | Western | | non-Western | | Western | |
|  | Avg | SD | Avg | SD | Avg | SD | Avg | SD | Avg | SD | Avg | SD |
| Firmicutes | 59.000 | 16.371 | 43.286 | 11.042 | 79.365 | 10.907 | 83.473 | 14.648 | 57.649 | 9.497 | 72.178 | 12.256 |
| Proteobacteria | 17.162 | 14.853 | 8.150 | 10.390 | 1.524 | 2.267 | 0.191 | 0.259 | 8.672 | 7.358 | 1.553 | 1.341 |
| Bacteroidetes | 10.285 | 11.252 | 28.264 | 10.083 | 15.212 | 7.496 | 11.791 | 13.686 | 29.438 | 9.598 | 22.336 | 11.889 |
| Spirochaetes | 8.315 | 9.712 | 17.979 | 17.762 | 3.235 | 6.341 | 0.000 | 0.000 | 0.996 | 2.239 | 0.000 | 0.000 |
| Actinobacteria | 1.623 | 2.077 | 0.971 | 1.252 | 0.059 | 0.094 | 4.318 | 3.752 | 1.203 | 2.712 | 2.472 | 3.092 |
| Tenericutes | 1.546 | 2.020 | 0.193 | 0.213 | 0.224 | 0.286 | 0.127 | 0.313 | 0.784 | 0.695 | 0.342 | 0.691 |
| Verrucomicrobia | 0.738 | 1.144 | 0.107 | 0.194 | 0.000 | 0.000 | 0.100 | 0.332 | 0.068 | 0.184 | 0.875 | 2.094 |
| Lentisphaerae | 0.508 | 0.525 | 0.064 | 0.139 | 0.147 | 0.237 | 0.000 | 0.000 | 0.141 | 0.341 | 0.011 | 0.041 |
| Cyanobacteria | 0.462 | 1.229 | 0.379 | 0.370 | 0.159 | 0.328 | 0.000 | 0.000 | 0.689 | 0.957 | 0.017 | 0.071 |
| WPS-2 | 0.246 | 0.627 | 0.414 | 0.934 | 0.047 | 0.118 | 0.000 | 0.000 | 0.004 | 0.034 | 0.000 | 0.000 |
| Fusobacteria | 0.069 | 0.197 | 0.014 | 0.036 | 0.018 | 0.073 | 0.000 | 0.000 | 0.025 | 0.082 | 0.148 | 1.537 |
| GN02 | 0.038 | 0.112 | 0.000 | 0.000 | 0.000 | 0.000 | 0.000 | 0.000 | 0.000 | 0.000 | 0.000 | 0.000 |
| Euryarchaeota | 0.000 | 0.000 | 0.000 | 0.000 | 0.000 | 0.000 | 0.000 | 0.000 | 0.082 | 0.171 | 0.059 | 0.142 |
| Acidobacteria | 0.000 | 0.000 | 0.000 | 0.000 | 0.000 | 0.000 | 0.000 | 0.000 | 0.000 | 0.000 | 0.001 | 0.009 |
| Chloroflexi | 0.000 | 0.000 | 0.007 | 0.027 | 0.000 | 0.000 | 0.000 | 0.000 | 0.000 | 0.000 | 0.000 | 0.000 |
| Elusimicrobia | 0.000 | 0.000 | 0.000 | 0.000 | 0.012 | 0.049 | 0.000 | 0.000 | 0.247 | 0.563 | 0.000 | 0.000 |
| Fibrobacteres | 0.000 | 0.000 | 0.021 | 0.058 | 0.000 | 0.000 | 0.000 | 0.000 | 0.000 | 0.000 | 0.000 | 0.000 |
| Synergistetes | 0.000 | 0.000 | 0.014 | 0.053 | 0.000 | 0.000 | 0.000 | 0.000 | 0.001 | 0.011 | 0.003 | 0.016 |
| TM7 | 0.000 | 0.000 | 0.136 | 0.210 | 0.000 | 0.000 | 0.000 | 0.000 | 0.000 | 0.000 | 0.005 | 0.022 |

Table S2. Average (± SD) relative abundance (%) of microbial classes in samples from vervets and humans consuming non-Western and Western diets. Western humans are from Italy (Schnorr et al. 2014) and the U.S. (Yatsunenko et al. 2012). Non-western humans are from Tanzania (Hadza, Schnorr et al. 2014), Venezuela (Guahibo, Yatsunenko et al. 2012), and Malawi (Yatsunenko et al. 2012).

|  | Amato et al. vervets | | | | Schnorr et al. humans | | | | Yatsunenko et al. humans | | | |
| --- | --- | --- | --- | --- | --- | --- | --- | --- | --- | --- | --- | --- |
|  | non-Western | | Western | | non-Western | | Western | | non-Western | | Western | |
|  | Avg | SD | Avg | SD | Avg | SD | Avg | SD | Avg | SD | Avg | SD |
| Clostridia | 55.608 | 21.021 | 37.493 | 10.667 | 76.759 | 10.195 | 80.182 | 14.700 | 52.967 | 9.960 | 69.114 | 12.322 |
| Gammaproteobacteria | 11.700 | 11.352 | 1.443 | 2.241 | 1.282 | 2.230 | 0.055 | 0.104 | 7.411 | 7.339 | 0.231 | 0.522 |
| Bacteroidia | 9.654 | 11.454 | 27.607 | 10.052 | 15.206 | 7.486 | 11.791 | 13.686 | 29.438 | 9.598 | 22.336 | 11.888 |
| Bacilli | 2.846 | 7.824 | 1.664 | 3.117 | 0.141 | 0.200 | 0.236 | 0.323 | 2.149 | 4.095 | 0.871 | 1.439 |
| Epsilonproteobacteria | 2.485 | 3.790 | 3.429 | 9.540 | 0.088 | 0.111 | 0.000 | 0.000 | 0.266 | 0.562 | 0.007 | 0.058 |
| Alphaproteobacteria | 1.538 | 3.183 | 0.800 | 0.858 | 0.012 | 0.033 | 0.018 | 0.060 | 0.257 | 0.741 | 0.135 | 0.498 |
| Actinobacteria | 1.438 | 1.940 | 0.500 | 1.251 | 0.029 | 0.059 | 4.191 | 3.828 | 0.746 | 2.553 | 1.974 | 3.040 |
| Betaproteobacteria | 1.438 | 2.804 | 1.700 | 1.549 | 0.024 | 0.056 | 0.100 | 0.141 | 0.514 | 0.474 | 0.816 | 0.935 |
| Mollicutes | 1.262 | 1.714 | 0.193 | 0.213 | 0.218 | 0.290 | 0.127 | 0.313 | 0.737 | 0.656 | 0.315 | 0.665 |
| Spirochaetes | 1.238 | 1.839 | 0.150 | 0.348 | 3.229 | 6.344 | 0.000 | 0.000 | 0.996 | 2.239 | 0.000 | 0.000 |
| Flavobacteriia | 0.631 | 1.901 | 0.657 | 1.811 | 0.000 | 0.000 | 0.000 | 0.000 | 0.000 | 0.000 | 0.000 | 0.000 |
| Opitutae | 0.577 | 0.839 | 0.000 | 0.000 | 0.000 | 0.000 | 0.000 | 0.000 | 0.008 | 0.032 | 0.000 | 0.000 |
| Erysipelotrichi | 0.546 | 0.539 | 4.129 | 2.012 | 2.465 | 3.249 | 3.055 | 3.160 | 2.533 | 2.089 | 2.192 | 2.256 |
| Chloroplast | 0.369 | 1.244 | 0.014 | 0.053 | 0.000 | 0.000 | 0.000 | 0.000 | 0.007 | 0.025 | 0.001 | 0.009 |
| RF3 | 0.285 | 0.682 | 0.000 | 0.000 | 0.006 | 0.024 | 0.000 | 0.000 | 0.047 | 0.163 | 0.027 | 0.099 |
| Coriobacteriia | 0.185 | 0.212 | 0.471 | 0.358 | 0.029 | 0.059 | 0.127 | 0.329 | 0.457 | 0.633 | 0.498 | 0.567 |
| Verrucomicrobiae | 0.162 | 0.393 | 0.079 | 0.185 | 0.000 | 0.000 | 0.100 | 0.332 | 0.028 | 0.154 | 0.875 | 2.094 |
| 4C0d-2 | 0.092 | 0.189 | 0.364 | 0.369 | 0.159 | 0.328 | 0.000 | 0.000 | 0.683 | 0.959 | 0.016 | 0.070 |
| Fusobacteriia | 0.069 | 0.197 | 0.014 | 0.036 | 0.018 | 0.073 | 0.000 | 0.000 | 0.025 | 0.082 | 0.148 | 1.537 |
| BD1-5 | 0.038 | 0.112 | 0.000 | 0.000 | 0.000 | 0.000 | 0.000 | 0.000 | 0.000 | 0.000 | 0.000 | 0.000 |
| Deinococci | 0.008 | 0.028 | 0.000 | 0.000 | 0.000 | 0.000 | 0.000 | 0.000 | 0.000 | 0.000 | 0.000 | 0.000 |
| Methanobacteria | 0.000 | 0.000 | 0.000 | 0.000 | 0.000 | 0.000 | 0.000 | 0.000 | 0.082 | 0.171 | 0.059 | 0.142 |
| Acidobacteriia | 0.000 | 0.000 | 0.000 | 0.000 | 0.000 | 0.000 | 0.000 | 0.000 | 0.000 | 0.000 | 0.001 | 0.009 |
| Anaerolineae | 0.000 | 0.000 | 0.007 | 0.027 | 0.000 | 0.000 | 0.000 | 0.000 | 0.000 | 0.000 | 0.000 | 0.000 |
| Elusimicrobia | 0.000 | 0.000 | 0.000 | 0.000 | 0.012 | 0.049 | 0.000 | 0.000 | 0.247 | 0.563 | 0.000 | 0.000 |
| Fibrobacteria | 0.000 | 0.000 | 0.021 | 0.058 | 0.000 | 0.000 | 0.000 | 0.000 | 0.000 | 0.000 | 0.000 | 0.000 |
| Deltaproteobacteria | 0.000 | 0.000 | 0.779 | 1.023 | 0.118 | 0.281 | 0.018 | 0.040 | 0.225 | 0.323 | 0.364 | 0.497 |
| Synergistia | 0.000 | 0.000 | 0.014 | 0.053 | 0.000 | 0.000 | 0.000 | 0.000 | 0.001 | 0.011 | 0.003 | 0.016 |
| TM7-3 | 0.000 | 0.000 | 0.136 | 0.210 | 0.000 | 0.000 | 0.000 | 0.000 | 0.000 | 0.000 | 0.000 | 0.000 |
| Verruco-5 | 0.000 | 0.000 | 0.029 | 0.083 | 0.000 | 0.000 | 0.000 | 0.000 | 0.033 | 0.096 | 0.000 | 0.000 |

Table S3. Average (± SD) relative abundance (%) of microbial orders in samples from vervets and humans consuming non-Western and Western diets. Western humans are from Italy (Schnorr et al. 2014) and the U.S. (Yatsunenko et al. 2012). Non-western humans are from Tanzania (Hadza, Schnorr et al. 2014), Venezuela (Guahibo, Yatsunenko et al. 2012), and Malawi (Yatsunenko et al. 2012).

|  | Amato et al. vervets | | | | Schnorr et al. humans | | | | Yatsunenko et al. humans | | | |
| --- | --- | --- | --- | --- | --- | --- | --- | --- | --- | --- | --- | --- |
|  | non-Western | | Western | | non-Western | | Western | | non-Western | | Western | |
|  | Avg | SD | Avg | SD | Avg | SD | Avg | SD | Avg | SD | Avg | SD |
| Clostridiales | 55.592 | 21.009 | 37.493 | 10.667 | 76.759 | 10.195 | 80.182 | 14.700 | 52.964 | 9.957 | 69.113 | 12.322 |
| Bacteroidales | 9.654 | 11.454 | 27.607 | 10.052 | 15.206 | 7.486 | 11.791 | 13.686 | 29.438 | 9.598 | 22.336 | 11.888 |
| Aeromonadales | 9.362 | 11.489 | 0.336 | 0.468 | 0.547 | 1.749 | 0.000 | 0.000 | 5.887 | 6.736 | 0.000 | 0.000 |
| Lactobacillales | 2.646 | 7.577 | 1.529 | 2.990 | 0.141 | 0.200 | 0.209 | 0.305 | 2.075 | 4.115 | 0.639 | 1.340 |
| Campylobacterales | 2.485 | 3.790 | 3.429 | 9.540 | 0.088 | 0.111 | 0.000 | 0.000 | 0.266 | 0.562 | 0.007 | 0.058 |
| Burkholderiales | 1.415 | 2.773 | 1.693 | 1.548 | 0.024 | 0.056 | 0.100 | 0.141 | 0.511 | 0.474 | 0.812 | 0.935 |
| Enterobacteriales | 1.169 | 2.520 | 0.214 | 0.218 | 0.571 | 1.204 | 0.055 | 0.104 | 1.184 | 3.535 | 0.172 | 0.451 |
| RF39 | 0.992 | 1.698 | 0.186 | 0.199 | 0.194 | 0.290 | 0.127 | 0.313 | 0.646 | 0.641 | 0.315 | 0.665 |
| Spirochaetales | 0.962 | 1.773 | 0.100 | 0.180 | 3.229 | 6.344 | 0.000 | 0.000 | 0.996 | 2.239 | 0.000 | 0.000 |
| Pseudomonadales | 0.938 | 3.028 | 0.793 | 2.054 | 0.000 | 0.000 | 0.000 | 0.000 | 0.034 | 0.298 | 0.000 | 0.000 |
| Bifidobacteriales | 0.846 | 1.718 | 0.000 | 0.000 | 0.012 | 0.033 | 4.182 | 3.818 | 0.720 | 2.548 | 1.953 | 3.028 |
| Rhodobacterales | 0.754 | 1.981 | 0.286 | 0.789 | 0.000 | 0.000 | 0.000 | 0.000 | 0.000 | 0.000 | 0.000 | 0.000 |
| Flavobacteriales | 0.631 | 1.901 | 0.657 | 1.811 | 0.000 | 0.000 | 0.000 | 0.000 | 0.000 | 0.000 | 0.000 | 0.000 |
| Actinomycetales | 0.592 | 1.014 | 0.500 | 1.251 | 0.018 | 0.053 | 0.009 | 0.030 | 0.026 | 0.077 | 0.020 | 0.050 |
| Erysipelotrichales | 0.546 | 0.539 | 4.129 | 2.012 | 2.465 | 3.249 | 3.055 | 3.160 | 2.533 | 2.089 | 2.192 | 2.256 |
| Victivallales | 0.508 | 0.525 | 0.021 | 0.043 | 0.076 | 0.120 | 0.000 | 0.000 | 0.132 | 0.321 | 0.011 | 0.041 |
| Streptophyta | 0.369 | 1.244 | 0.014 | 0.053 | 0.000 | 0.000 | 0.000 | 0.000 | 0.007 | 0.025 | 0.001 | 0.009 |
| ML615J-28 | 0.285 | 0.682 | 0.000 | 0.000 | 0.006 | 0.024 | 0.000 | 0.000 | 0.047 | 0.163 | 0.027 | 0.099 |
| RF32 | 0.285 | 0.416 | 0.136 | 0.287 | 0.012 | 0.033 | 0.018 | 0.060 | 0.197 | 0.731 | 0.135 | 0.498 |
| Sphaerochaetales | 0.277 | 0.517 | 0.050 | 0.187 | 0.000 | 0.000 | 0.000 | 0.000 | 0.000 | 0.000 | 0.000 | 0.000 |
| Sphingomonadales | 0.269 | 0.852 | 0.086 | 0.266 | 0.000 | 0.000 | 0.000 | 0.000 | 0.000 | 0.000 | 0.000 | 0.000 |
| Anaeroplasmatales | 0.269 | 0.598 | 0.007 | 0.027 | 0.024 | 0.097 | 0.000 | 0.000 | 0.087 | 0.178 | 0.000 | 0.000 |
| Coriobacteriales | 0.185 | 0.212 | 0.471 | 0.358 | 0.029 | 0.059 | 0.127 | 0.329 | 0.457 | 0.633 | 0.498 | 0.567 |
| Bacillales | 0.177 | 0.309 | 0.121 | 0.246 | 0.000 | 0.000 | 0.000 | 0.000 | 0.014 | 0.051 | 0.006 | 0.040 |
| Rhizobiales | 0.177 | 0.332 | 0.064 | 0.108 | 0.000 | 0.000 | 0.000 | 0.000 | 0.000 | 0.000 | 0.000 | 0.000 |
| Xanthomonadales | 0.177 | 0.444 | 0.100 | 0.215 | 0.000 | 0.000 | 0.000 | 0.000 | 0.001 | 0.011 | 0.000 | 0.000 |
| Verrucomicrobiales | 0.162 | 0.393 | 0.079 | 0.185 | 0.000 | 0.000 | 0.100 | 0.332 | 0.028 | 0.154 | 0.875 | 2.094 |
| YS2 | 0.092 | 0.189 | 0.343 | 0.382 | 0.159 | 0.328 | 0.000 | 0.000 | 0.683 | 0.959 | 0.016 | 0.070 |
| Fusobacteriales | 0.069 | 0.197 | 0.014 | 0.036 | 0.018 | 0.073 | 0.000 | 0.000 | 0.025 | 0.082 | 0.148 | 1.537 |
| Alteromonadales | 0.046 | 0.120 | 0.000 | 0.000 | 0.000 | 0.000 | 0.000 | 0.000 | 0.000 | 0.000 | 0.000 | 0.000 |
| Caulobacterales | 0.031 | 0.111 | 0.007 | 0.027 | 0.000 | 0.000 | 0.000 | 0.000 | 0.000 | 0.000 | 0.000 | 0.000 |
| Turicibacterales | 0.023 | 0.060 | 0.007 | 0.027 | 0.000 | 0.000 | 0.027 | 0.065 | 0.058 | 0.102 | 0.224 | 0.536 |
| Rickettsiales | 0.015 | 0.055 | 0.000 | 0.000 | 0.000 | 0.000 | 0.000 | 0.000 | 0.001 | 0.011 | 0.000 | 0.000 |
| Rhodocyclales | 0.015 | 0.038 | 0.000 | 0.000 | 0.000 | 0.000 | 0.000 | 0.000 | 0.001 | 0.011 | 0.002 | 0.013 |
| Rhodospirillales | 0.008 | 0.028 | 0.000 | 0.000 | 0.000 | 0.000 | 0.000 | 0.000 | 0.000 | 0.000 | 0.000 | 0.000 |
| Methylophilales | 0.008 | 0.028 | 0.000 | 0.000 | 0.000 | 0.000 | 0.000 | 0.000 | 0.000 | 0.000 | 0.000 | 0.000 |
| Oceanospirillales | 0.008 | 0.028 | 0.000 | 0.000 | 0.000 | 0.000 | 0.000 | 0.000 | 0.001 | 0.011 | 0.000 | 0.000 |
| Deinococcales | 0.008 | 0.028 | 0.000 | 0.000 | 0.000 | 0.000 | 0.000 | 0.000 | 0.000 | 0.000 | 0.000 | 0.000 |
| Methanobacteriales | 0.000 | 0.000 | 0.000 | 0.000 | 0.000 | 0.000 | 0.000 | 0.000 | 0.082 | 0.171 | 0.059 | 0.142 |
| Acidobacteriales | 0.000 | 0.000 | 0.000 | 0.000 | 0.000 | 0.000 | 0.000 | 0.000 | 0.000 | 0.000 | 0.001 | 0.009 |
| Anaerolineales | 0.000 | 0.000 | 0.007 | 0.027 | 0.000 | 0.000 | 0.000 | 0.000 | 0.000 | 0.000 | 0.000 | 0.000 |
| MLE1-12 | 0.000 | 0.000 | 0.021 | 0.080 | 0.000 | 0.000 | 0.000 | 0.000 | 0.000 | 0.000 | 0.000 | 0.000 |
| Elusimicrobiales | 0.000 | 0.000 | 0.000 | 0.000 | 0.012 | 0.049 | 0.000 | 0.000 | 0.247 | 0.563 | 0.000 | 0.000 |
| Fibrobacterales | 0.000 | 0.000 | 0.021 | 0.058 | 0.000 | 0.000 | 0.000 | 0.000 | 0.000 | 0.000 | 0.000 | 0.000 |
| Gemellales | 0.000 | 0.000 | 0.007 | 0.027 | 0.000 | 0.000 | 0.000 | 0.000 | 0.001 | 0.011 | 0.003 | 0.016 |
| SHA-98 | 0.000 | 0.000 | 0.000 | 0.000 | 0.000 | 0.000 | 0.000 | 0.000 | 0.000 | 0.000 | 0.002 | 0.018 |
| Z20 | 0.000 | 0.000 | 0.043 | 0.134 | 0.071 | 0.220 | 0.000 | 0.000 | 0.009 | 0.037 | 0.000 | 0.000 |
| Ellin329 | 0.000 | 0.000 | 0.000 | 0.000 | 0.000 | 0.000 | 0.000 | 0.000 | 0.000 | 0.000 | 0.000 | 0.000 |
| Gallionellales | 0.000 | 0.000 | 0.000 | 0.000 | 0.000 | 0.000 | 0.000 | 0.000 | 0.001 | 0.011 | 0.001 | 0.009 |
| Neisseriales | 0.000 | 0.000 | 0.007 | 0.027 | 0.000 | 0.000 | 0.000 | 0.000 | 0.001 | 0.011 | 0.000 | 0.000 |
| Procabacteriales | 0.000 | 0.000 | 0.000 | 0.000 | 0.000 | 0.000 | 0.000 | 0.000 | 0.000 | 0.000 | 0.000 | 0.000 |
| Desulfovibrionales | 0.000 | 0.000 | 0.779 | 1.023 | 0.024 | 0.066 | 0.018 | 0.040 | 0.193 | 0.267 | 0.364 | 0.497 |
| GMD14H09 | 0.000 | 0.000 | 0.000 | 0.000 | 0.094 | 0.238 | 0.000 | 0.000 | 0.032 | 0.160 | 0.000 | 0.000 |
| Pasteurellales | 0.000 | 0.000 | 0.000 | 0.000 | 0.165 | 0.291 | 0.000 | 0.000 | 0.296 | 0.578 | 0.057 | 0.206 |
| Thiohalorhabdales | 0.000 | 0.000 | 0.000 | 0.000 | 0.000 | 0.000 | 0.000 | 0.000 | 0.005 | 0.028 | 0.002 | 0.013 |
| Synergistales | 0.000 | 0.000 | 0.014 | 0.053 | 0.000 | 0.000 | 0.000 | 0.000 | 0.001 | 0.011 | 0.003 | 0.016 |
| CW040 | 0.000 | 0.000 | 0.136 | 0.210 | 0.000 | 0.000 | 0.000 | 0.000 | 0.000 | 0.000 | 0.000 | 0.000 |
| WCHB1-41 | 0.000 | 0.000 | 0.029 | 0.083 | 0.000 | 0.000 | 0.000 | 0.000 | 0.033 | 0.096 | 0.000 | 0.000 |

Table S4. Average (± SD) relative abundance (%) of microbial families in samples from vervets and humans consuming non-Western and Western diets. Western humans are from Italy (Schnorr et al. 2014) and the U.S. (Yatsunenko et al. 2012). Non-western humans are from Tanzania (Hadza, Schnorr et al. 2014), Venezuela (Guahibo, Yatsunenko et al. 2012), and Malawi (Yatsunenko et al. 2012).

|  | Amato et al. vervets | | | | Schnorr et al. humans | | | | | | Yatsunenko et al. humans | | | | | | | |  |
| --- | --- | --- | --- | --- | --- | --- | --- | --- | --- | --- | --- | --- | --- | --- | --- | --- | --- | --- | --- |
|  | non-Western | | Western | | non-Western | | Western | | | | non-Western | | | | Western | | | |  |
|  | Avg | SD | Avg | SD | Avg | SD | Avg | | SD | | Avg | | SD | | Avg | | SD | |  |
| Ruminococcaceae | 24.485 | 12.520 | 16.036 | 6.940 | 51.535 | 11.432 | | 40.373 | | 18.816 | | 22.787 | | 6.803 | | 30.419 | | 10.707 | |
| Lachnospiraceae | 10.962 | 8.653 | 10.336 | 5.265 | 15.965 | 5.118 | | 29.982 | | 9.558 | | 16.187 | | 7.533 | | 27.773 | | 11.075 | |
| Succinivibrionaceae | 9.331 | 11.516 | 0.307 | 0.476 | 0.547 | 1.749 | | 0.000 | | 0.000 | | 5.887 | | 6.736 | | 0.000 | | 0.000 | |
| Clostridiaceae | 7.454 | 6.787 | 1.950 | 1.250 | 1.724 | 1.067 | | 2.064 | | 2.306 | | 5.426 | | 4.086 | | 2.563 | | 2.235 | |
| Brachyspiraceae | 7.077 | 9.387 | 17.829 | 17.761 | 0.006 | 0.024 | | 0.000 | | 0.000 | | 0.000 | | 0.000 | | 0.000 | | 0.000 | |
| Prevotellaceae | 5.569 | 9.781 | 21.400 | 8.874 | 8.112 | 8.567 | | 0.391 | | 0.718 | | 23.709 | | 10.706 | | 1.808 | | 5.778 | |
| Bacteroidaceae | 2.269 | 4.592 | 0.300 | 0.359 | 0.224 | 0.313 | | 9.855 | | 11.402 | | 0.905 | | 2.503 | | 16.160 | | 10.785 | |
| Streptococcaceae | 1.931 | 6.102 | 0.271 | 0.640 | 0.082 | 0.178 | | 0.173 | | 0.261 | | 1.395 | | 3.048 | | 0.549 | | 1.263 | |
| Campylobacteraceae | 1.823 | 3.430 | 0.629 | 1.085 | 0.082 | 0.113 | | 0.000 | | 0.000 | | 0.262 | | 0.563 | | 0.007 | | 0.058 | |
| Veillonellaceae | 1.462 | 2.226 | 2.007 | 1.003 | 0.718 | 0.991 | | 0.291 | | 0.378 | | 3.407 | | 2.144 | | 2.268 | | 3.278 | |
| Enterobacteriaceae | 1.169 | 2.520 | 0.214 | 0.218 | 0.571 | 1.204 | | 0.055 | | 0.104 | | 1.184 | | 3.535 | | 0.172 | | 0.451 | |
| Comamonadaceae | 1.000 | 2.649 | 1.043 | 1.551 | 0.000 | 0.000 | | 0.000 | | 0.000 | | 0.012 | | 0.054 | | 0.002 | | 0.013 | |
| Spirochaetaceae | 0.962 | 1.773 | 0.100 | 0.180 | 3.229 | 6.344 | | 0.000 | | 0.000 | | 0.996 | | 2.239 | | 0.000 | | 0.000 | |
| Bifidobacteriaceae | 0.846 | 1.718 | 0.000 | 0.000 | 0.012 | 0.033 | | 4.182 | | 3.818 | | 0.720 | | 2.548 | | 1.953 | | 3.028 | |
| Rhodobacteraceae | 0.754 | 1.981 | 0.286 | 0.789 | 0.000 | 0.000 | | 0.000 | | 0.000 | | 0.000 | | 0.000 | | 0.000 | | 0.000 | |
| Helicobacteraceae | 0.662 | 1.835 | 2.800 | 9.674 | 0.006 | 0.024 | | 0.000 | | 0.000 | | 0.004 | | 0.026 | | 0.000 | | 0.000 | |
| Moraxellaceae | 0.662 | 2.237 | 0.364 | 0.836 | 0.000 | 0.000 | | 0.000 | | 0.000 | | 0.034 | | 0.298 | | 0.000 | | 0.000 | |
| Erysipelotrichaceae | 0.546 | 0.539 | 4.129 | 2.012 | 2.465 | 3.249 | | 3.055 | | 3.160 | | 2.533 | | 2.089 | | 2.192 | | 2.256 | |
| Porphyromonadaceae | 0.531 | 1.161 | 0.221 | 0.302 | 0.341 | 0.780 | | 0.727 | | 1.795 | | 0.218 | | 0.369 | | 1.732 | | 2.251 | |
| Victivallaceae | 0.508 | 0.525 | 0.021 | 0.043 | 0.076 | 0.120 | | 0.000 | | 0.000 | | 0.132 | | 0.321 | | 0.011 | | 0.041 | |
| Leuconostocaceae | 0.431 | 1.465 | 1.007 | 2.815 | 0.006 | 0.024 | | 0.009 | | 0.030 | | 0.058 | | 0.372 | | 0.003 | | 0.028 | |
| Propionibacteriaceae | 0.415 | 0.925 | 0.293 | 0.844 | 0.000 | 0.000 | | 0.000 | | 0.000 | | 0.000 | | 0.000 | | 0.000 | | 0.000 | |
| Alcaligenaceae | 0.315 | 0.348 | 0.629 | 0.341 | 0.024 | 0.056 | | 0.100 | | 0.141 | | 0.483 | | 0.462 | | 0.797 | | 0.934 | |
| Peptostreptococcaceae | 0.315 | 0.316 | 0.029 | 0.107 | 0.047 | 0.087 | | 0.336 | | 0.843 | | 0.032 | | 0.094 | | 0.027 | | 0.089 | |
| Pseudomonadaceae | 0.277 | 0.796 | 0.429 | 1.329 | 0.000 | 0.000 | | 0.000 | | 0.000 | | 0.000 | | 0.000 | | 0.000 | | 0.000 | |
| Sphaerochaetaceae | 0.277 | 0.517 | 0.050 | 0.187 | 0.000 | 0.000 | | 0.000 | | 0.000 | | 0.000 | | 0.000 | | 0.000 | | 0.000 | |
| Anaeroplasmataceae | 0.269 | 0.598 | 0.007 | 0.027 | 0.024 | 0.097 | | 0.000 | | 0.000 | | 0.087 | | 0.178 | | 0.000 | | 0.000 | |
| Sphingomonadaceae | 0.254 | 0.796 | 0.086 | 0.266 | 0.000 | 0.000 | | 0.000 | | 0.000 | | 0.000 | | 0.000 | | 0.000 | | 0.000 | |
| Christensenellaceae | 0.238 | 0.384 | 0.107 | 0.107 | 0.459 | 0.735 | | 0.018 | | 0.060 | | 0.251 | | 0.630 | | 0.547 | | 1.243 | |
| S24-7 | 0.231 | 0.475 | 0.500 | 0.709 | 0.424 | 0.641 | | 0.000 | | 0.000 | | 0.924 | | 2.042 | | 0.208 | | 0.732 | |
| Lactobacillaceae | 0.223 | 0.344 | 0.221 | 0.345 | 0.053 | 0.133 | | 0.000 | | 0.000 | | 0.343 | | 0.796 | | 0.051 | | 0.380 | |
| RF16 | 0.215 | 0.297 | 0.479 | 0.414 | 0.247 | 0.697 | | 0.000 | | 0.000 | | 0.141 | | 1.068 | | 0.000 | | 0.000 | |
| Coriobacteriaceae | 0.185 | 0.212 | 0.471 | 0.358 | 0.029 | 0.059 | | 0.127 | | 0.329 | | 0.457 | | 0.633 | | 0.498 | | 0.567 | |
| Xanthomonadaceae | 0.177 | 0.444 | 0.100 | 0.215 | 0.000 | 0.000 | | 0.000 | | 0.000 | | 0.001 | | 0.011 | | 0.000 | | 0.000 | |
| Verrucomicrobiaceae | 0.162 | 0.393 | 0.079 | 0.185 | 0.000 | 0.000 | | 0.100 | | 0.332 | | 0.028 | | 0.154 | | 0.875 | | 2.094 | |
| Actinomycetaceae | 0.077 | 0.205 | 0.036 | 0.074 | 0.018 | 0.053 | | 0.009 | | 0.030 | | 0.013 | | 0.055 | | 0.012 | | 0.035 | |
| Corynebacteriaceae | 0.077 | 0.196 | 0.043 | 0.065 | 0.000 | 0.000 | | 0.000 | | 0.000 | | 0.008 | | 0.039 | | 0.002 | | 0.013 | |
| Oxalobacteraceae | 0.077 | 0.159 | 0.014 | 0.036 | 0.000 | 0.000 | | 0.000 | | 0.000 | | 0.013 | | 0.034 | | 0.013 | | 0.061 | |
| Staphylococcaceae | 0.069 | 0.155 | 0.064 | 0.115 | 0.000 | 0.000 | | 0.000 | | 0.000 | | 0.000 | | 0.000 | | 0.000 | | 0.000 | |
| Fusobacteriaceae | 0.062 | 0.194 | 0.014 | 0.036 | 0.000 | 0.000 | | 0.000 | | 0.000 | | 0.021 | | 0.074 | | 0.146 | | 1.537 | |
| Paenibacillaceae | 0.054 | 0.133 | 0.014 | 0.053 | 0.000 | 0.000 | | 0.000 | | 0.000 | | 0.003 | | 0.023 | | 0.001 | | 0.009 | |
| Bacillaceae | 0.046 | 0.113 | 0.007 | 0.027 | 0.000 | 0.000 | | 0.000 | | 0.000 | | 0.011 | | 0.039 | | 0.000 | | 0.000 | |
| Aerococcaceae | 0.046 | 0.139 | 0.014 | 0.053 | 0.000 | 0.000 | | 0.000 | | 0.000 | | 0.003 | | 0.016 | | 0.001 | | 0.009 | |
| Methylobacteriaceae | 0.046 | 0.097 | 0.000 | 0.000 | 0.000 | 0.000 | | 0.000 | | 0.000 | | 0.000 | | 0.000 | | 0.000 | | 0.000 | |
| Bradyrhizobiaceae | 0.038 | 0.096 | 0.007 | 0.027 | 0.000 | 0.000 | | 0.000 | | 0.000 | | 0.000 | | 0.000 | | 0.000 | | 0.000 | |
| Rhizobiaceae | 0.038 | 0.077 | 0.029 | 0.061 | 0.000 | 0.000 | | 0.000 | | 0.000 | | 0.000 | | 0.000 | | 0.000 | | 0.000 | |
| Rikenellaceae | 0.031 | 0.111 | 0.021 | 0.058 | 0.012 | 0.049 | | 0.373 | | 0.450 | | 0.042 | | 0.187 | | 1.628 | | 1.474 | |
| Flavobacteriaceae | 0.031 | 0.075 | 0.007 | 0.027 | 0.000 | 0.000 | | 0.000 | | 0.000 | | 0.000 | | 0.000 | | 0.000 | | 0.000 | |
| Caulobacteraceae | 0.031 | 0.111 | 0.007 | 0.027 | 0.000 | 0.000 | | 0.000 | | 0.000 | | 0.000 | | 0.000 | | 0.000 | | 0.000 | |
| Aeromonadaceae | 0.031 | 0.111 | 0.029 | 0.073 | 0.000 | 0.000 | | 0.000 | | 0.000 | | 0.000 | | 0.000 | | 0.000 | | 0.000 | |
| Shewanellaceae | 0.031 | 0.075 | 0.000 | 0.000 | 0.000 | 0.000 | | 0.000 | | 0.000 | | 0.000 | | 0.000 | | 0.000 | | 0.000 | |
| Turicibacteraceae | 0.023 | 0.060 | 0.007 | 0.027 | 0.000 | 0.000 | | 0.027 | | 0.065 | | 0.058 | | 0.102 | | 0.224 | | 0.536 | |
| Phyllobacteriaceae | 0.023 | 0.044 | 0.014 | 0.036 | 0.000 | 0.000 | | 0.000 | | 0.000 | | 0.000 | | 0.000 | | 0.000 | | 0.000 | |
| Micrococcaceae | 0.015 | 0.055 | 0.114 | 0.374 | 0.000 | 0.000 | | 0.000 | | 0.000 | | 0.005 | | 0.022 | | 0.007 | | 0.034 | |
| Enterococcaceae | 0.015 | 0.038 | 0.014 | 0.053 | 0.000 | 0.000 | | 0.018 | | 0.060 | | 0.067 | | 0.343 | | 0.007 | | 0.043 | |
| Peptococcaceae | 0.015 | 0.038 | 0.029 | 0.061 | 0.006 | 0.024 | | 0.018 | | 0.060 | | 0.051 | | 0.122 | | 0.054 | | 0.132 | |
| Rhodocyclaceae | 0.015 | 0.038 | 0.000 | 0.000 | 0.000 | 0.000 | | 0.000 | | 0.000 | | 0.001 | | 0.011 | | 0.002 | | 0.013 | |
| Microbacteriaceae | 0.008 | 0.028 | 0.000 | 0.000 | 0.000 | 0.000 | | 0.000 | | 0.000 | | 0.000 | | 0.000 | | 0.000 | | 0.000 | |
| Planococcaceae | 0.008 | 0.028 | 0.021 | 0.058 | 0.000 | 0.000 | | 0.000 | | 0.000 | | 0.000 | | 0.000 | | 0.003 | | 0.018 | |
| Dehalobacteriaceae | 0.008 | 0.028 | 0.000 | 0.000 | 0.018 | 0.039 | | 0.000 | | 0.000 | | 0.007 | | 0.025 | | 0.008 | | 0.030 | |
| Eubacteriaceae | 0.008 | 0.028 | 0.000 | 0.000 | 0.000 | 0.000 | | 0.000 | | 0.000 | | 0.001 | | 0.011 | | 0.003 | | 0.022 | |
| Leptotrichiaceae | 0.008 | 0.028 | 0.000 | 0.000 | 0.018 | 0.073 | | 0.000 | | 0.000 | | 0.004 | | 0.020 | | 0.003 | | 0.021 | |
| Methylocystaceae | 0.008 | 0.028 | 0.000 | 0.000 | 0.000 | 0.000 | | 0.000 | | 0.000 | | 0.000 | | 0.000 | | 0.000 | | 0.000 | |
| Rhodospirillaceae | 0.008 | 0.028 | 0.000 | 0.000 | 0.000 | 0.000 | | 0.000 | | 0.000 | | 0.000 | | 0.000 | | 0.000 | | 0.000 | |
| Burkholderiaceae | 0.008 | 0.028 | 0.007 | 0.027 | 0.000 | 0.000 | | 0.000 | | 0.000 | | 0.001 | | 0.011 | | 0.000 | | 0.000 | |
| Methylophilaceae | 0.008 | 0.028 | 0.000 | 0.000 | 0.000 | 0.000 | | 0.000 | | 0.000 | | 0.000 | | 0.000 | | 0.000 | | 0.000 | |
| Oceanospirillaceae | 0.008 | 0.028 | 0.000 | 0.000 | 0.000 | 0.000 | | 0.000 | | 0.000 | | 0.001 | | 0.011 | | 0.000 | | 0.000 | |
| Deinococcaceae | 0.008 | 0.028 | 0.000 | 0.000 | 0.000 | 0.000 | | 0.000 | | 0.000 | | 0.000 | | 0.000 | | 0.000 | | 0.000 | |
| Methanobacteriaceae | 0.000 | 0.000 | 0.000 | 0.000 | 0.000 | 0.000 | | 0.000 | | 0.000 | | 0.082 | | 0.171 | | 0.059 | | 0.142 | |
| Acidobacteriaceae | 0.000 | 0.000 | 0.000 | 0.000 | 0.000 | 0.000 | | 0.000 | | 0.000 | | 0.000 | | 0.000 | | 0.001 | | 0.009 | |
| Intrasporangiaceae | 0.000 | 0.000 | 0.014 | 0.053 | 0.000 | 0.000 | | 0.000 | | 0.000 | | 0.000 | | 0.000 | | 0.000 | | 0.000 | |
| Marinilabiaceae | 0.000 | 0.000 | 0.000 | 0.000 | 0.000 | 0.000 | | 0.000 | | 0.000 | | 0.000 | | 0.000 | | 0.001 | | 0.009 | |
| p-2534-18B5 | 0.000 | 0.000 | 0.079 | 0.189 | 0.124 | 0.317 | | 0.000 | | 0.000 | | 0.039 | | 0.241 | | 0.000 | | 0.000 | |
| Chitinophagaceae | 0.000 | 0.000 | 0.000 | 0.000 | 0.006 | 0.024 | | 0.000 | | 0.000 | | 0.000 | | 0.000 | | 0.001 | | 0.009 | |
| Anaerolinaceae | 0.000 | 0.000 | 0.007 | 0.027 | 0.000 | 0.000 | | 0.000 | | 0.000 | | 0.000 | | 0.000 | | 0.000 | | 0.000 | |
| Elusimicrobiaceae | 0.000 | 0.000 | 0.000 | 0.000 | 0.012 | 0.049 | | 0.000 | | 0.000 | | 0.247 | | 0.563 | | 0.000 | | 0.000 | |
| Fibrobacteraceae | 0.000 | 0.000 | 0.021 | 0.058 | 0.000 | 0.000 | | 0.000 | | 0.000 | | 0.000 | | 0.000 | | 0.000 | | 0.000 | |
| Listeriaceae | 0.000 | 0.000 | 0.014 | 0.053 | 0.000 | 0.000 | | 0.000 | | 0.000 | | 0.000 | | 0.000 | | 0.000 | | 0.000 | |
| Gemellaceae | 0.000 | 0.000 | 0.007 | 0.027 | 0.000 | 0.000 | | 0.000 | | 0.000 | | 0.001 | | 0.011 | | 0.003 | | 0.016 | |
| EtOH8 | 0.000 | 0.000 | 0.000 | 0.000 | 0.000 | 0.000 | | 0.000 | | 0.000 | | 0.000 | | 0.000 | | 0.003 | | 0.021 | |
| R4-45B | 0.000 | 0.000 | 0.043 | 0.134 | 0.071 | 0.220 | | 0.000 | | 0.000 | | 0.009 | | 0.037 | | 0.000 | | 0.000 | |
| Erythrobacteraceae | 0.000 | 0.000 | 0.000 | 0.000 | 0.000 | 0.000 | | 0.000 | | 0.000 | | 0.000 | | 0.000 | | 0.000 | | 0.000 | |
| Gallionellaceae | 0.000 | 0.000 | 0.000 | 0.000 | 0.000 | 0.000 | | 0.000 | | 0.000 | | 0.001 | | 0.011 | | 0.001 | | 0.009 | |
| Neisseriaceae | 0.000 | 0.000 | 0.007 | 0.027 | 0.000 | 0.000 | | 0.000 | | 0.000 | | 0.001 | | 0.011 | | 0.000 | | 0.000 | |
| Procabacteriaceae | 0.000 | 0.000 | 0.000 | 0.000 | 0.000 | 0.000 | | 0.000 | | 0.000 | | 0.000 | | 0.000 | | 0.000 | | 0.000 | |
| Desulfovibrionaceae | 0.000 | 0.000 | 0.779 | 1.023 | 0.024 | 0.066 | | 0.018 | | 0.040 | | 0.193 | | 0.267 | | 0.364 | | 0.497 | |
| Pasteurellaceae | 0.000 | 0.000 | 0.000 | 0.000 | 0.147 | 0.292 | | 0.000 | | 0.000 | | 0.296 | | 0.578 | | 0.057 | | 0.206 | |
| Synergistaceae | 0.000 | 0.000 | 0.014 | 0.053 | 0.000 | 0.000 | | 0.000 | | 0.000 | | 0.001 | | 0.011 | | 0.003 | | 0.016 | |
| F16 | 0.000 | 0.000 | 0.129 | 0.213 | 0.000 | 0.000 | | 0.000 | | 0.000 | | 0.000 | | 0.000 | | 0.000 | | 0.000 | |
| RFP12 | 0.000 | 0.000 | 0.029 | 0.083 | 0.000 | 0.000 | | 0.000 | | 0.000 | | 0.033 | | 0.096 | | 0.000 | | 0.000 | |

Table S5. Average (± SD) relative abundance (%) of microbial genera in samples from vervets and humans consuming non-Western and Western diets. Western humans are from Italy (Schnorr et al. 2014) and the U.S. (Yatsunenko et al. 2012). Non-western humans are from Tanzania (Hadza, Schnorr et al. 2014), Venezuela (Guahibo, Yatsunenko et al. 2012), and Malawi (Yatsunenko et al. 2012).

|  | Amato et al. vervets | | | | Schnorr et al. humans | | | | Yatsunenko et al. humans | | | |
| --- | --- | --- | --- | --- | --- | --- | --- | --- | --- | --- | --- | --- |
|  | non-Western | | Western | | non-Western | | Western | | non-Western | | Western | |
|  | Avg | SD | Avg | SD | Avg | SD | Avg | SD | Avg | SD | Avg | SD |
| *Succinivibrio* | 9.07 | 11.34 | 0.19 | 0.22 | 0.54 | 1.75 | 0.00 | 0.00 | 4.35 | 5.50 | 0.00 | 0.00 |
| *Brachyspira* | 7.08 | 9.39 | 17.83 | 17.76 | 0.01 | 0.02 | 0.00 | 0.00 | 0.00 | 0.00 | 0.00 | 0.00 |
| *Ruminococcus* | 6.05 | 4.86 | 2.52 | 3.22 | 1.86 | 1.73 | 3.29 | 3.03 | 2.85 | 2.31 | 7.28 | 5.93 |
| *Prevotella* | 5.55 | 9.76 | 21.39 | 8.88 | 8.11 | 8.57 | 0.39 | 0.72 | 23.63 | 10.74 | 1.81 | 5.78 |
| *Clostridium* | 5.28 | 5.10 | 0.41 | 0.37 | 0.91 | 0.60 | 0.53 | 0.65 | 0.65 | 0.60 | 0.58 | 0.72 |
| *Coprococcus* | 2.83 | 3.35 | 1.19 | 0.80 | 0.79 | 0.70 | 3.43 | 3.08 | 1.08 | 0.87 | 1.61 | 1.24 |
| *Bacteroides* | 2.27 | 4.59 | 0.30 | 0.36 | 0.22 | 0.31 | 9.85 | 11.40 | 0.91 | 2.50 | 16.16 | 10.79 |
| *Lactococcus* | 1.89 | 6.08 | 0.06 | 0.19 | 0.00 | 0.00 | 0.00 | 0.00 | 0.05 | 0.25 | 0.02 | 0.08 |
| *Campylobacter* | 1.71 | 3.16 | 0.59 | 1.09 | 0.08 | 0.11 | 0.00 | 0.00 | 0.26 | 0.56 | 0.01 | 0.06 |
| *Faecalibacterium* | 1.45 | 1.50 | 2.83 | 1.70 | 5.54 | 4.32 | 3.31 | 4.57 | 5.79 | 3.77 | 3.63 | 3.05 |
| *Blautia* | 1.25 | 1.25 | 2.53 | 1.68 | 4.93 | 3.62 | 6.87 | 5.75 | 3.75 | 4.65 | 6.45 | 4.66 |
| *Oscillospira* | 0.98 | 0.79 | 1.44 | 0.83 | 0.89 | 0.88 | 1.38 | 1.48 | 0.50 | 0.43 | 0.91 | 0.86 |
| *Treponema* | 0.96 | 1.77 | 0.10 | 0.18 | 3.23 | 6.34 | 0.00 | 0.00 | 1.00 | 2.24 | 0.00 | 0.00 |
| *Bifidobacterium* | 0.85 | 1.72 | 0.00 | 0.00 | 0.01 | 0.03 | 4.18 | 3.82 | 0.72 | 2.55 | 1.94 | 3.02 |
| *Paracoccus* | 0.70 | 1.87 | 0.24 | 0.74 | 0.00 | 0.00 | 0.00 | 0.00 | 0.00 | 0.00 | 0.00 | 0.00 |
| *Roseburia* | 0.68 | 1.38 | 0.35 | 0.36 | 0.82 | 0.89 | 1.96 | 4.16 | 0.84 | 0.97 | 0.92 | 1.10 |
| *Acinetobacter* | 0.65 | 2.18 | 0.31 | 0.69 | 0.00 | 0.00 | 0.00 | 0.00 | 0.03 | 0.29 | 0.00 | 0.00 |
| *Flexispira* | 0.64 | 1.81 | 0.65 | 1.67 | 0.01 | 0.02 | 0.00 | 0.00 | 0.00 | 0.00 | 0.00 | 0.00 |
| *Dialister* | 0.57 | 1.18 | 0.07 | 0.14 | 0.01 | 0.03 | 0.05 | 0.10 | 2.00 | 1.78 | 1.06 | 1.62 |
| *Lachnospira* | 0.41 | 0.69 | 0.09 | 0.16 | 0.28 | 0.39 | 1.09 | 1.03 | 0.94 | 0.94 | 0.99 | 1.23 |
| *Propionibacterium* | 0.40 | 0.92 | 0.29 | 0.84 | 0.00 | 0.00 | 0.00 | 0.00 | 0.00 | 0.00 | 0.00 | 0.00 |
| *Leuconostoc* | 0.40 | 1.38 | 0.96 | 2.71 | 0.01 | 0.02 | 0.00 | 0.00 | 0.00 | 0.00 | 0.00 | 0.03 |
| *Cloacibacterium* | 0.33 | 0.91 | 0.60 | 1.73 | 0.00 | 0.00 | 0.00 | 0.00 | 0.00 | 0.00 | 0.00 | 0.00 |
| *Megasphaera* | 0.32 | 0.86 | 0.03 | 0.11 | 0.00 | 0.00 | 0.10 | 0.25 | 0.22 | 0.58 | 0.53 | 2.57 |
| *Porphyromonas* | 0.31 | 1.05 | 0.01 | 0.05 | 0.00 | 0.00 | 0.00 | 0.00 | 0.01 | 0.04 | 0.00 | 0.00 |
| *Sutterella* | 0.30 | 0.30 | 0.63 | 0.34 | 0.02 | 0.06 | 0.10 | 0.14 | 0.48 | 0.46 | 0.80 | 0.93 |
| *Dorea* | 0.29 | 0.30 | 1.12 | 1.02 | 0.65 | 0.50 | 1.45 | 1.32 | 0.44 | 0.40 | 1.34 | 1.21 |
| *Sphaerochaeta* | 0.28 | 0.52 | 0.05 | 0.19 | 0.00 | 0.00 | 0.00 | 0.00 | 0.00 | 0.00 | 0.00 | 0.00 |
| *Pseudomonas* | 0.24 | 0.66 | 0.39 | 1.23 | 0.00 | 0.00 | 0.00 | 0.00 | 0.00 | 0.00 | 0.00 | 0.00 |
| *Parabacteroides* | 0.22 | 0.57 | 0.21 | 0.31 | 0.34 | 0.78 | 0.73 | 1.80 | 0.21 | 0.37 | 1.73 | 2.25 |
| *Lactobacillus* | 0.22 | 0.34 | 0.21 | 0.35 | 0.05 | 0.13 | 0.00 | 0.00 | 0.34 | 0.80 | 0.05 | 0.38 |
| *Anaerobiospirillum* | 0.22 | 0.80 | 0.11 | 0.28 | 0.00 | 0.00 | 0.00 | 0.00 | 0.00 | 0.00 | 0.00 | 0.00 |
| WAL_1855D | 0.19 | 0.39 | 0.39 | 0.87 | 0.00 | 0.00 | 0.00 | 0.00 | 0.00 | 0.00 | 0.00 | 0.02 |
| *Phascolarctobacterium* | 0.17 | 0.16 | 0.95 | 0.78 | 0.41 | 0.45 | 0.02 | 0.06 | 0.52 | 0.54 | 0.28 | 0.42 |
| *Akkermansia* | 0.16 | 0.39 | 0.08 | 0.18 | 0.00 | 0.00 | 0.10 | 0.33 | 0.03 | 0.15 | 0.87 | 2.09 |
| *Butyricimonas* | 0.15 | 0.50 | 0.01 | 0.03 | 0.06 | 0.10 | 0.14 | 0.25 | 0.02 | 0.05 | 0.08 | 0.24 |
| *Anaerococcus* | 0.15 | 0.27 | 0.31 | 0.76 | 0.00 | 0.00 | 0.00 | 0.00 | 0.00 | 0.02 | 0.00 | 0.01 |
| *Stenotrophomonas* | 0.15 | 0.37 | 0.08 | 0.20 | 0.00 | 0.00 | 0.00 | 0.00 | 0.00 | 0.00 | 0.00 | 0.00 |
| *Sphingomonas* | 0.14 | 0.47 | 0.01 | 0.03 | 0.00 | 0.00 | 0.00 | 0.00 | 0.00 | 0.00 | 0.00 | 0.00 |
| *Butyrivibrio* | 0.12 | 0.41 | 0.04 | 0.06 | 0.02 | 0.08 | 0.00 | 0.00 | 0.00 | 0.02 | 0.00 | 0.02 |
| *Victivallis* | 0.11 | 0.18 | 0.01 | 0.04 | 0.04 | 0.09 | 0.00 | 0.00 | 0.01 | 0.05 | 0.00 | 0.01 |
| *Comamonas* | 0.11 | 0.26 | 0.31 | 0.64 | 0.00 | 0.00 | 0.00 | 0.00 | 0.00 | 0.01 | 0.00 | 0.00 |
| *Collinsella* | 0.09 | 0.14 | 0.41 | 0.37 | 0.01 | 0.02 | 0.07 | 0.21 | 0.21 | 0.50 | 0.28 | 0.45 |
| *Diaphorobacter* | 0.08 | 0.28 | 0.11 | 0.21 | 0.00 | 0.00 | 0.00 | 0.00 | 0.00 | 0.00 | 0.00 | 0.00 |
| *Peptoniphilus* | 0.08 | 0.20 | 0.03 | 0.08 | 0.00 | 0.00 | 0.00 | 0.00 | 0.00 | 0.00 | 0.01 | 0.03 |
| RFN20 | 0.08 | 0.17 | 0.01 | 0.03 | 0.00 | 0.00 | 0.00 | 0.00 | 0.02 | 0.08 | 0.00 | 0.05 |
| *Corynebacterium* | 0.08 | 0.20 | 0.04 | 0.06 | 0.00 | 0.00 | 0.00 | 0.00 | 0.01 | 0.04 | 0.00 | 0.01 |
| ph2 | 0.07 | 0.18 | 0.00 | 0.00 | 0.00 | 0.00 | 0.00 | 0.00 | 0.00 | 0.01 | 0.00 | 0.00 |
| *Arcobacter* | 0.07 | 0.25 | 0.02 | 0.08 | 0.00 | 0.00 | 0.00 | 0.00 | 0.00 | 0.00 | 0.00 | 0.00 |
| *Staphylococcus* | 0.06 | 0.16 | 0.06 | 0.12 | 0.00 | 0.00 | 0.00 | 0.00 | 0.00 | 0.00 | 0.00 | 0.00 |
| 02d06 | 0.06 | 0.11 | 0.01 | 0.03 | 0.00 | 0.00 | 0.00 | 0.00 | 0.01 | 0.03 | 0.23 | 0.27 |
| *Fusobacterium* | 0.06 | 0.19 | 0.01 | 0.04 | 0.00 | 0.00 | 0.00 | 0.00 | 0.02 | 0.07 | 0.00 | 0.02 |
| *Paenibacillus* | 0.05 | 0.13 | 0.01 | 0.05 | 0.00 | 0.00 | 0.00 | 0.00 | 0.00 | 0.01 | 0.00 | 0.00 |
| *Anaerostipes* | 0.05 | 0.08 | 0.04 | 0.09 | 0.22 | 0.69 | 0.19 | 0.22 | 0.02 | 0.06 | 0.14 | 0.16 |
| *Novosphingobium* | 0.05 | 0.14 | 0.06 | 0.19 | 0.00 | 0.00 | 0.00 | 0.00 | 0.00 | 0.00 | 0.00 | 0.00 |
| *Aerococcus* | 0.05 | 0.14 | 0.01 | 0.05 | 0.00 | 0.00 | 0.00 | 0.00 | 0.00 | 0.00 | 0.00 | 0.00 |
| *Sarcina* | 0.05 | 0.09 | 0.04 | 0.06 | 0.00 | 0.00 | 0.00 | 0.00 | 0.02 | 0.04 | 0.00 | 0.02 |
| *Bulleidia* | 0.05 | 0.14 | 0.17 | 0.16 | 0.14 | 0.16 | 0.00 | 0.00 | 0.31 | 0.42 | 0.00 | 0.03 |
| *Sphingobium* | 0.05 | 0.17 | 0.01 | 0.05 | 0.00 | 0.00 | 0.00 | 0.00 | 0.00 | 0.00 | 0.00 | 0.00 |
| *Streptococcus* | 0.04 | 0.07 | 0.21 | 0.63 | 0.08 | 0.18 | 0.17 | 0.26 | 1.34 | 3.06 | 0.53 | 1.26 |
| *Anaerovibrio* | 0.04 | 0.14 | 0.71 | 0.37 | 0.25 | 0.66 | 0.00 | 0.00 | 0.23 | 0.53 | 0.00 | 0.00 |
| *Ruminobacter* | 0.04 | 0.10 | 0.00 | 0.00 | 0.01 | 0.03 | 0.00 | 0.00 | 1.53 | 3.58 | 0.00 | 0.00 |
| *Escherichia* | 0.04 | 0.11 | 0.01 | 0.04 | 0.00 | 0.00 | 0.00 | 0.00 | 0.00 | 0.00 | 0.00 | 0.00 |
| *Actinomyces* | 0.03 | 0.11 | 0.01 | 0.03 | 0.02 | 0.05 | 0.00 | 0.00 | 0.01 | 0.05 | 0.01 | 0.03 |
| *Shewanella* | 0.03 | 0.08 | 0.00 | 0.00 | 0.00 | 0.00 | 0.00 | 0.00 | 0.00 | 0.00 | 0.00 | 0.00 |
| *Arcanobacterium* | 0.02 | 0.08 | 0.00 | 0.00 | 0.00 | 0.00 | 0.00 | 0.00 | 0.00 | 0.00 | 0.00 | 0.00 |
| *Mobiluncus* | 0.02 | 0.08 | 0.00 | 0.00 | 0.00 | 0.00 | 0.00 | 0.00 | 0.00 | 0.00 | 0.00 | 0.00 |
| CF231 | 0.02 | 0.08 | 0.75 | 0.65 | 0.10 | 0.26 | 0.00 | 0.00 | 0.19 | 0.37 | 0.00 | 0.00 |
| *Anoxybacillus* | 0.02 | 0.06 | 0.00 | 0.00 | 0.00 | 0.00 | 0.00 | 0.00 | 0.00 | 0.01 | 0.00 | 0.00 |
| *Bacillus* | 0.02 | 0.06 | 0.01 | 0.03 | 0.00 | 0.00 | 0.00 | 0.00 | 0.00 | 0.02 | 0.00 | 0.00 |
| *Turicibacter* | 0.02 | 0.06 | 0.01 | 0.03 | 0.00 | 0.00 | 0.03 | 0.06 | 0.06 | 0.10 | 0.22 | 0.54 |
| *Rhodobacter* | 0.02 | 0.08 | 0.02 | 0.06 | 0.00 | 0.00 | 0.00 | 0.00 | 0.00 | 0.00 | 0.00 | 0.00 |
| *Helicobacter* | 0.02 | 0.06 | 2.13 | 7.96 | 0.00 | 0.00 | 0.00 | 0.00 | 0.00 | 0.03 | 0.00 | 0.00 |
| *Adlercreutzia* | 0.02 | 0.06 | 0.00 | 0.00 | 0.00 | 0.00 | 0.00 | 0.00 | 0.00 | 0.00 | 0.04 | 0.08 |
| *Enterococcus* | 0.02 | 0.04 | 0.01 | 0.05 | 0.00 | 0.00 | 0.02 | 0.06 | 0.06 | 0.33 | 0.01 | 0.04 |
| SMB53 | 0.02 | 0.06 | 0.00 | 0.00 | 0.00 | 0.00 | 0.13 | 0.42 | 0.58 | 0.67 | 0.66 | 0.96 |
| *Lachnobacterium* | 0.02 | 0.04 | 0.01 | 0.03 | 0.01 | 0.03 | 0.10 | 0.25 | 0.25 | 0.64 | 0.34 | 0.72 |
| p-75-a5 | 0.02 | 0.06 | 0.86 | 0.70 | 0.26 | 0.44 | 0.00 | 0.00 | 0.12 | 0.25 | 0.00 | 0.00 |
| *Afipia* | 0.02 | 0.06 | 0.00 | 0.00 | 0.00 | 0.00 | 0.00 | 0.00 | 0.00 | 0.00 | 0.00 | 0.00 |
| *Balneimonas* | 0.02 | 0.06 | 0.00 | 0.00 | 0.00 | 0.00 | 0.00 | 0.00 | 0.00 | 0.00 | 0.00 | 0.00 |
| *Agrobacterium* | 0.02 | 0.06 | 0.01 | 0.03 | 0.00 | 0.00 | 0.00 | 0.00 | 0.00 | 0.00 | 0.00 | 0.00 |
| *Achromobacter* | 0.02 | 0.06 | 0.00 | 0.00 | 0.00 | 0.00 | 0.00 | 0.00 | 0.00 | 0.00 | 0.00 | 0.00 |
| *Oxalobacter* | 0.02 | 0.06 | 0.00 | 0.00 | 0.00 | 0.00 | 0.00 | 0.00 | 0.01 | 0.03 | 0.01 | 0.06 |
| *Microbacterium* | 0.01 | 0.03 | 0.00 | 0.00 | 0.00 | 0.00 | 0.00 | 0.00 | 0.00 | 0.00 | 0.00 | 0.00 |
| *Micrococcus* | 0.01 | 0.03 | 0.00 | 0.00 | 0.00 | 0.00 | 0.00 | 0.00 | 0.00 | 0.00 | 0.00 | 0.00 |
| *Rothia* | 0.01 | 0.03 | 0.04 | 0.16 | 0.00 | 0.00 | 0.00 | 0.00 | 0.01 | 0.02 | 0.01 | 0.03 |
| YRC22 | 0.01 | 0.03 | 0.01 | 0.04 | 0.08 | 0.14 | 0.00 | 0.00 | 0.05 | 0.24 | 0.00 | 0.00 |
| *Jeotgalicoccus* | 0.01 | 0.03 | 0.01 | 0.03 | 0.00 | 0.00 | 0.00 | 0.00 | 0.00 | 0.00 | 0.00 | 0.00 |
| *Dehalobacterium* | 0.01 | 0.03 | 0.00 | 0.00 | 0.02 | 0.04 | 0.00 | 0.00 | 0.01 | 0.02 | 0.01 | 0.03 |
| *Anaerofustis* | 0.01 | 0.03 | 0.00 | 0.00 | 0.00 | 0.00 | 0.00 | 0.00 | 0.00 | 0.01 | 0.00 | 0.00 |
| *Mogibacterium* | 0.01 | 0.03 | 0.07 | 0.12 | 0.00 | 0.00 | 0.00 | 0.00 | 0.00 | 0.01 | 0.00 | 0.02 |
| *Allobaculum* | 0.01 | 0.03 | 0.04 | 0.13 | 0.00 | 0.00 | 0.00 | 0.00 | 0.00 | 0.00 | 0.00 | 0.02 |
| *Coprobacillus* | 0.01 | 0.03 | 0.00 | 0.00 | 0.00 | 0.00 | 0.03 | 0.06 | 0.01 | 0.07 | 0.04 | 0.14 |
| *Holdemania* | 0.01 | 0.03 | 0.00 | 0.00 | 0.00 | 0.00 | 0.02 | 0.04 | 0.00 | 0.01 | 0.04 | 0.07 |
| *Sneathia* | 0.01 | 0.03 | 0.00 | 0.00 | 0.02 | 0.07 | 0.00 | 0.00 | 0.00 | 0.02 | 0.00 | 0.00 |
| *Mycoplana* | 0.01 | 0.03 | 0.00 | 0.00 | 0.00 | 0.00 | 0.00 | 0.00 | 0.00 | 0.00 | 0.00 | 0.00 |
| *Bradyrhizobium* | 0.01 | 0.03 | 0.01 | 0.03 | 0.00 | 0.00 | 0.00 | 0.00 | 0.00 | 0.00 | 0.00 | 0.00 |
| *Methylobacterium* | 0.01 | 0.03 | 0.00 | 0.00 | 0.00 | 0.00 | 0.00 | 0.00 | 0.00 | 0.00 | 0.00 | 0.00 |
| *Pleomorphomonas* | 0.01 | 0.03 | 0.00 | 0.00 | 0.00 | 0.00 | 0.00 | 0.00 | 0.00 | 0.00 | 0.00 | 0.00 |
| *Mesorhizobium* | 0.01 | 0.03 | 0.01 | 0.04 | 0.00 | 0.00 | 0.00 | 0.00 | 0.00 | 0.00 | 0.00 | 0.00 |
| *Amorphomonas* | 0.01 | 0.03 | 0.00 | 0.00 | 0.00 | 0.00 | 0.00 | 0.00 | 0.00 | 0.00 | 0.00 | 0.00 |
| *Kaistobacter* | 0.01 | 0.03 | 0.00 | 0.00 | 0.00 | 0.00 | 0.00 | 0.00 | 0.00 | 0.00 | 0.00 | 0.00 |
| *Burkholderia* | 0.01 | 0.03 | 0.00 | 0.00 | 0.00 | 0.00 | 0.00 | 0.00 | 0.00 | 0.01 | 0.00 | 0.00 |
| *Alicycliphilus* | 0.01 | 0.03 | 0.02 | 0.08 | 0.00 | 0.00 | 0.00 | 0.00 | 0.00 | 0.00 | 0.00 | 0.00 |
| *Pelomonas* | 0.01 | 0.03 | 0.00 | 0.00 | 0.00 | 0.00 | 0.00 | 0.00 | 0.00 | 0.00 | 0.00 | 0.00 |
| *Herbaspirillum* | 0.01 | 0.03 | 0.00 | 0.00 | 0.00 | 0.00 | 0.00 | 0.00 | 0.00 | 0.00 | 0.00 | 0.00 |
| *Janthinobacterium* | 0.01 | 0.03 | 0.00 | 0.00 | 0.00 | 0.00 | 0.00 | 0.00 | 0.00 | 0.00 | 0.00 | 0.00 |
| *Massilia* | 0.01 | 0.03 | 0.00 | 0.00 | 0.00 | 0.00 | 0.00 | 0.00 | 0.00 | 0.00 | 0.00 | 0.00 |
| *Ralstonia* | 0.01 | 0.03 | 0.01 | 0.04 | 0.00 | 0.00 | 0.00 | 0.00 | 0.00 | 0.00 | 0.00 | 0.00 |
| *Methylobacillus* | 0.01 | 0.03 | 0.00 | 0.00 | 0.00 | 0.00 | 0.00 | 0.00 | 0.00 | 0.00 | 0.00 | 0.00 |
| *Dechloromonas* | 0.01 | 0.03 | 0.00 | 0.00 | 0.00 | 0.00 | 0.00 | 0.00 | 0.00 | 0.00 | 0.00 | 0.00 |
| *Hydrogenophilus* | 0.01 | 0.03 | 0.00 | 0.00 | 0.00 | 0.00 | 0.00 | 0.00 | 0.00 | 0.00 | 0.00 | 0.00 |
| *Erwinia* | 0.01 | 0.03 | 0.00 | 0.00 | 0.00 | 0.00 | 0.00 | 0.00 | 0.00 | 0.01 | 0.00 | 0.02 |
| *Ewingella* | 0.01 | 0.03 | 0.00 | 0.00 | 0.00 | 0.00 | 0.00 | 0.00 | 0.00 | 0.00 | 0.00 | 0.00 |
| *Shigella* | 0.01 | 0.03 | 0.00 | 0.00 | 0.00 | 0.00 | 0.00 | 0.00 | 0.00 | 0.00 | 0.00 | 0.00 |
| *Trabulsiella* | 0.01 | 0.03 | 0.00 | 0.00 | 0.00 | 0.00 | 0.00 | 0.00 | 0.00 | 0.01 | 0.00 | 0.00 |
| *Marinomonas* | 0.01 | 0.03 | 0.00 | 0.00 | 0.00 | 0.00 | 0.00 | 0.00 | 0.00 | 0.00 | 0.00 | 0.00 |
| *Enhydrobacter* | 0.01 | 0.03 | 0.01 | 0.03 | 0.00 | 0.00 | 0.00 | 0.00 | 0.00 | 0.00 | 0.00 | 0.00 |
| *Deinococcus* | 0.01 | 0.03 | 0.00 | 0.00 | 0.00 | 0.00 | 0.00 | 0.00 | 0.00 | 0.00 | 0.00 | 0.00 |
| *Methanobrevibacter* | 0.00 | 0.00 | 0.00 | 0.00 | 0.00 | 0.00 | 0.00 | 0.00 | 0.05 | 0.14 | 0.06 | 0.14 |
| *Methanosphaera* | 0.00 | 0.00 | 0.00 | 0.00 | 0.00 | 0.00 | 0.00 | 0.00 | 0.03 | 0.08 | 0.00 | 0.01 |
| *Terriglobus* | 0.00 | 0.00 | 0.00 | 0.00 | 0.00 | 0.00 | 0.00 | 0.00 | 0.00 | 0.00 | 0.00 | 0.01 |
| *Varibaculum* | 0.00 | 0.00 | 0.03 | 0.07 | 0.00 | 0.00 | 0.01 | 0.03 | 0.00 | 0.02 | 0.00 | 0.01 |
| *Janibacter* | 0.00 | 0.00 | 0.01 | 0.05 | 0.00 | 0.00 | 0.00 | 0.00 | 0.00 | 0.00 | 0.00 | 0.00 |
| *Kocuria* | 0.00 | 0.00 | 0.01 | 0.05 | 0.00 | 0.00 | 0.00 | 0.00 | 0.00 | 0.00 | 0.00 | 0.00 |
| *Propionimicrobium* | 0.00 | 0.00 | 0.00 | 0.00 | 0.00 | 0.00 | 0.00 | 0.00 | 0.00 | 0.00 | 0.00 | 0.00 |
| *Atopobium* | 0.00 | 0.00 | 0.00 | 0.00 | 0.00 | 0.00 | 0.00 | 0.00 | 0.00 | 0.02 | 0.00 | 0.01 |
| *Eggerthella* | 0.00 | 0.00 | 0.00 | 0.00 | 0.00 | 0.00 | 0.00 | 0.00 | 0.00 | 0.00 | 0.03 | 0.07 |
| *Slackia* | 0.00 | 0.00 | 0.00 | 0.00 | 0.00 | 0.00 | 0.00 | 0.00 | 0.02 | 0.08 | 0.01 | 0.04 |
| *Dysgonomonas* | 0.00 | 0.00 | 0.00 | 0.00 | 0.00 | 0.00 | 0.00 | 0.00 | 0.00 | 0.00 | 0.00 | 0.02 |
| *Paludibacter* | 0.00 | 0.00 | 0.00 | 0.00 | 0.00 | 0.00 | 0.00 | 0.00 | 0.00 | 0.00 | 0.00 | 0.02 |
| *Alistipes* | 0.00 | 0.00 | 0.00 | 0.00 | 0.00 | 0.00 | 0.10 | 0.27 | 0.00 | 0.00 | 0.01 | 0.06 |
| *Odoribacter* | 0.00 | 0.00 | 0.00 | 0.00 | 0.01 | 0.03 | 0.09 | 0.11 | 0.01 | 0.03 | 0.10 | 0.16 |
| *Paraprevotella* | 0.00 | 0.00 | 0.00 | 0.00 | 0.00 | 0.00 | 0.06 | 0.15 | 0.00 | 0.02 | 0.10 | 0.36 |
| *Chryseobacterium* | 0.00 | 0.00 | 0.00 | 0.00 | 0.00 | 0.00 | 0.00 | 0.00 | 0.00 | 0.00 | 0.00 | 0.00 |
| *Flavisolibacter* | 0.00 | 0.00 | 0.00 | 0.00 | 0.01 | 0.02 | 0.00 | 0.00 | 0.00 | 0.00 | 0.00 | 0.00 |
| SHD-231 | 0.00 | 0.00 | 0.01 | 0.03 | 0.00 | 0.00 | 0.00 | 0.00 | 0.00 | 0.00 | 0.00 | 0.00 |
| *Elusimicrobium* | 0.00 | 0.00 | 0.00 | 0.00 | 0.01 | 0.05 | 0.00 | 0.00 | 0.00 | 0.00 | 0.00 | 0.00 |
| *Fibrobacter* | 0.00 | 0.00 | 0.02 | 0.06 | 0.00 | 0.00 | 0.00 | 0.00 | 0.00 | 0.00 | 0.00 | 0.00 |
| *Geobacillus* | 0.00 | 0.00 | 0.00 | 0.00 | 0.00 | 0.00 | 0.00 | 0.00 | 0.00 | 0.02 | 0.00 | 0.00 |
| *Brochothrix* | 0.00 | 0.00 | 0.01 | 0.05 | 0.00 | 0.00 | 0.00 | 0.00 | 0.00 | 0.00 | 0.00 | 0.00 |
| *Brevibacillus* | 0.00 | 0.00 | 0.00 | 0.00 | 0.00 | 0.00 | 0.00 | 0.00 | 0.00 | 0.01 | 0.00 | 0.01 |
| *Rummeliibacillus* | 0.00 | 0.00 | 0.01 | 0.03 | 0.00 | 0.00 | 0.00 | 0.00 | 0.00 | 0.00 | 0.00 | 0.00 |
| *Gemella* | 0.00 | 0.00 | 0.00 | 0.00 | 0.00 | 0.00 | 0.00 | 0.00 | 0.00 | 0.00 | 0.00 | 0.01 |
| *Granulicatella* | 0.00 | 0.00 | 0.00 | 0.00 | 0.00 | 0.00 | 0.00 | 0.00 | 0.01 | 0.03 | 0.01 | 0.02 |
| *Trichococcus* | 0.00 | 0.00 | 0.00 | 0.00 | 0.00 | 0.00 | 0.00 | 0.00 | 0.00 | 0.01 | 0.00 | 0.00 |
| *Tetragenococcus* | 0.00 | 0.00 | 0.00 | 0.00 | 0.00 | 0.00 | 0.00 | 0.00 | 0.00 | 0.01 | 0.00 | 0.00 |
| *Vagococcus* | 0.00 | 0.00 | 0.00 | 0.00 | 0.00 | 0.00 | 0.00 | 0.00 | 0.00 | 0.02 | 0.00 | 0.00 |
| *Pediococcus* | 0.00 | 0.00 | 0.01 | 0.04 | 0.00 | 0.00 | 0.00 | 0.00 | 0.00 | 0.00 | 0.00 | 0.01 |
| *Weissella* | 0.00 | 0.00 | 0.04 | 0.11 | 0.00 | 0.00 | 0.01 | 0.03 | 0.00 | 0.01 | 0.00 | 0.00 |
| *Christensenella* | 0.00 | 0.00 | 0.00 | 0.00 | 0.00 | 0.00 | 0.00 | 0.00 | 0.00 | 0.00 | 0.01 | 0.02 |
| *Pseudoramibacter_Eubacterium* | 0.00 | 0.00 | 0.00 | 0.00 | 0.00 | 0.00 | 0.00 | 0.00 | 0.00 | 0.00 | 0.00 | 0.02 |
| *Clostridium* | 0.00 | 0.00 | 0.00 | 0.00 | 0.00 | 0.00 | 0.00 | 0.00 | 0.00 | 0.02 | 0.00 | 0.00 |
| *Epulopiscium* | 0.00 | 0.00 | 0.00 | 0.00 | 0.00 | 0.00 | 0.00 | 0.00 | 0.00 | 0.00 | 0.01 | 0.06 |
| *Moryella* | 0.00 | 0.00 | 0.00 | 0.00 | 0.00 | 0.00 | 0.00 | 0.00 | 0.00 | 0.01 | 0.00 | 0.00 |
| *Oribacterium* | 0.00 | 0.00 | 0.00 | 0.00 | 0.00 | 0.00 | 0.00 | 0.00 | 0.00 | 0.01 | 0.00 | 0.01 |
| *Desulfosporosinus* | 0.00 | 0.00 | 0.00 | 0.00 | 0.00 | 0.00 | 0.00 | 0.00 | 0.00 | 0.00 | 0.00 | 0.01 |
| *Peptococcus* | 0.00 | 0.00 | 0.03 | 0.06 | 0.01 | 0.02 | 0.02 | 0.06 | 0.04 | 0.12 | 0.03 | 0.13 |
| rc4-4 | 0.00 | 0.00 | 0.00 | 0.00 | 0.00 | 0.00 | 0.00 | 0.00 | 0.00 | 0.00 | 0.01 | 0.03 |
| *Clostridium* | 0.00 | 0.00 | 0.00 | 0.00 | 0.00 | 0.00 | 0.00 | 0.00 | 0.00 | 0.02 | 0.00 | 0.01 |
| *Anaerotruncus* | 0.00 | 0.00 | 0.00 | 0.00 | 0.00 | 0.00 | 0.00 | 0.00 | 0.00 | 0.00 | 0.02 | 0.05 |
| *Butyricicoccus* | 0.00 | 0.00 | 0.00 | 0.00 | 0.00 | 0.00 | 0.00 | 0.00 | 0.00 | 0.01 | 0.00 | 0.00 |
| *Clostridium* | 0.00 | 0.00 | 0.00 | 0.00 | 0.00 | 0.00 | 0.00 | 0.00 | 0.00 | 0.00 | 0.01 | 0.03 |
| *Acidaminococcus* | 0.00 | 0.00 | 0.00 | 0.00 | 0.01 | 0.02 | 0.03 | 0.09 | 0.00 | 0.00 | 0.20 | 0.80 |
| *Megamonas* | 0.00 | 0.00 | 0.00 | 0.00 | 0.00 | 0.00 | 0.00 | 0.00 | 0.00 | 0.01 | 0.04 | 0.25 |
| *Mitsuokella* | 0.00 | 0.00 | 0.00 | 0.00 | 0.00 | 0.00 | 0.08 | 0.27 | 0.00 | 0.00 | 0.00 | 0.00 |
| *Selenomonas* | 0.00 | 0.00 | 0.00 | 0.00 | 0.00 | 0.00 | 0.00 | 0.00 | 0.00 | 0.01 | 0.00 | 0.01 |
| *Succiniclasticum* | 0.00 | 0.00 | 0.00 | 0.00 | 0.00 | 0.00 | 0.00 | 0.00 | 0.00 | 0.00 | 0.04 | 0.22 |
| *Veillonella* | 0.00 | 0.00 | 0.01 | 0.03 | 0.00 | 0.00 | 0.00 | 0.00 | 0.23 | 0.74 | 0.04 | 0.20 |
| *Finegoldia* | 0.00 | 0.00 | 0.00 | 0.00 | 0.00 | 0.00 | 0.00 | 0.00 | 0.00 | 0.00 | 0.00 | 0.00 |
| *Helcococcus* | 0.00 | 0.00 | 0.00 | 0.00 | 0.00 | 0.00 | 0.00 | 0.00 | 0.00 | 0.00 | 0.00 | 0.00 |
| *Parvimonas* | 0.00 | 0.00 | 0.00 | 0.00 | 0.00 | 0.00 | 0.00 | 0.00 | 0.00 | 0.00 | 0.00 | 0.02 |
| *Catenibacterium* | 0.00 | 0.00 | 1.26 | 1.31 | 0.39 | 0.52 | 0.00 | 0.00 | 0.61 | 0.99 | 0.15 | 0.73 |
| *Clostridium* | 0.00 | 0.00 | 0.00 | 0.00 | 0.00 | 0.00 | 0.00 | 0.00 | 0.00 | 0.00 | 0.01 | 0.03 |
| *Sharpea* | 0.00 | 0.00 | 0.01 | 0.03 | 0.00 | 0.00 | 0.00 | 0.00 | 0.00 | 0.00 | 0.00 | 0.00 |
| cc_115 | 0.00 | 0.00 | 0.00 | 0.00 | 0.00 | 0.00 | 0.04 | 0.07 | 0.00 | 0.00 | 0.03 | 0.10 |
| *Cetobacterium* | 0.00 | 0.00 | 0.00 | 0.00 | 0.00 | 0.00 | 0.00 | 0.00 | 0.01 | 0.03 | 0.14 | 1.54 |
| *Leptotrichia* | 0.00 | 0.00 | 0.00 | 0.00 | 0.00 | 0.00 | 0.00 | 0.00 | 0.00 | 0.01 | 0.00 | 0.02 |
| *Brevundimonas* | 0.00 | 0.00 | 0.01 | 0.03 | 0.00 | 0.00 | 0.00 | 0.00 | 0.00 | 0.00 | 0.00 | 0.00 |
| *Azospirillum* | 0.00 | 0.00 | 0.00 | 0.00 | 0.00 | 0.00 | 0.00 | 0.00 | 0.00 | 0.00 | 0.00 | 0.00 |
| *Lautropia* | 0.00 | 0.00 | 0.01 | 0.03 | 0.00 | 0.00 | 0.00 | 0.00 | 0.00 | 0.00 | 0.00 | 0.00 |
| *Acidovorax* | 0.00 | 0.00 | 0.00 | 0.00 | 0.00 | 0.00 | 0.00 | 0.00 | 0.00 | 0.00 | 0.00 | 0.00 |
| *Aquabacterium* | 0.00 | 0.00 | 0.00 | 0.00 | 0.00 | 0.00 | 0.00 | 0.00 | 0.00 | 0.00 | 0.00 | 0.00 |
| *Methylibium* | 0.00 | 0.00 | 0.02 | 0.08 | 0.00 | 0.00 | 0.00 | 0.00 | 0.00 | 0.00 | 0.00 | 0.00 |
| *Rhodoferax* | 0.00 | 0.00 | 0.00 | 0.00 | 0.00 | 0.00 | 0.00 | 0.00 | 0.01 | 0.05 | 0.00 | 0.01 |
| *Variovorax* | 0.00 | 0.00 | 0.00 | 0.00 | 0.00 | 0.00 | 0.00 | 0.00 | 0.00 | 0.00 | 0.00 | 0.00 |
| *Gallionella* | 0.00 | 0.00 | 0.00 | 0.00 | 0.00 | 0.00 | 0.00 | 0.00 | 0.00 | 0.01 | 0.00 | 0.01 |
| *Microvirgula* | 0.00 | 0.00 | 0.00 | 0.00 | 0.00 | 0.00 | 0.00 | 0.00 | 0.00 | 0.00 | 0.00 | 0.00 |
| *Neisseria* | 0.00 | 0.00 | 0.00 | 0.00 | 0.00 | 0.00 | 0.00 | 0.00 | 0.00 | 0.01 | 0.00 | 0.00 |
| *Vogesella* | 0.00 | 0.00 | 0.01 | 0.03 | 0.00 | 0.00 | 0.00 | 0.00 | 0.00 | 0.00 | 0.00 | 0.00 |
| *Azoarcus* | 0.00 | 0.00 | 0.00 | 0.00 | 0.00 | 0.00 | 0.00 | 0.00 | 0.00 | 0.00 | 0.00 | 0.01 |
| *Zoogloea* | 0.00 | 0.00 | 0.00 | 0.00 | 0.00 | 0.00 | 0.00 | 0.00 | 0.00 | 0.00 | 0.00 | 0.01 |
| *Bilophila* | 0.00 | 0.00 | 0.00 | 0.00 | 0.00 | 0.00 | 0.02 | 0.04 | 0.03 | 0.06 | 0.18 | 0.30 |
| *Desulfovibrio* | 0.00 | 0.00 | 0.77 | 1.02 | 0.02 | 0.07 | 0.00 | 0.00 | 0.16 | 0.25 | 0.18 | 0.41 |
| *Wolinella* | 0.00 | 0.00 | 0.01 | 0.05 | 0.00 | 0.00 | 0.00 | 0.00 | 0.00 | 0.00 | 0.00 | 0.00 |
| *Aeromonas* | 0.00 | 0.00 | 0.01 | 0.05 | 0.00 | 0.00 | 0.00 | 0.00 | 0.00 | 0.00 | 0.00 | 0.00 |
| *Citrobacter* | 0.00 | 0.00 | 0.01 | 0.05 | 0.00 | 0.00 | 0.00 | 0.00 | 0.00 | 0.01 | 0.00 | 0.00 |
| *Enterobacter* | 0.00 | 0.00 | 0.00 | 0.00 | 0.01 | 0.03 | 0.00 | 0.00 | 0.00 | 0.02 | 0.00 | 0.00 |
| *Gluconacetobacter* | 0.00 | 0.00 | 0.04 | 0.12 | 0.00 | 0.00 | 0.00 | 0.00 | 0.00 | 0.00 | 0.00 | 0.00 |
| *Klebsiella* | 0.00 | 0.00 | 0.00 | 0.00 | 0.00 | 0.00 | 0.00 | 0.00 | 0.01 | 0.03 | 0.01 | 0.04 |
| *Pantoea* | 0.00 | 0.00 | 0.00 | 0.00 | 0.00 | 0.00 | 0.01 | 0.03 | 0.00 | 0.01 | 0.00 | 0.00 |
| *Providencia* | 0.00 | 0.00 | 0.01 | 0.03 | 0.00 | 0.00 | 0.00 | 0.00 | 0.00 | 0.01 | 0.00 | 0.00 |
| *Serratia* | 0.00 | 0.00 | 0.01 | 0.03 | 0.00 | 0.00 | 0.00 | 0.00 | 0.01 | 0.03 | 0.01 | 0.07 |
| *Amphritea* | 0.00 | 0.00 | 0.00 | 0.00 | 0.00 | 0.00 | 0.00 | 0.00 | 0.00 | 0.01 | 0.00 | 0.00 |
| *Aggregatibacter* | 0.00 | 0.00 | 0.00 | 0.00 | 0.00 | 0.00 | 0.00 | 0.00 | 0.01 | 0.03 | 0.00 | 0.01 |
| *Haemophilus* | 0.00 | 0.00 | 0.00 | 0.00 | 0.15 | 0.29 | 0.00 | 0.00 | 0.29 | 0.57 | 0.06 | 0.20 |
| *Ignatzschineria* | 0.00 | 0.00 | 0.00 | 0.00 | 0.00 | 0.00 | 0.00 | 0.00 | 0.00 | 0.01 | 0.00 | 0.00 |
| *Luteimonas* | 0.00 | 0.00 | 0.01 | 0.03 | 0.00 | 0.00 | 0.00 | 0.00 | 0.00 | 0.00 | 0.00 | 0.00 |
| *Cloacibacillus* | 0.00 | 0.00 | 0.00 | 0.00 | 0.00 | 0.00 | 0.00 | 0.00 | 0.00 | 0.00 | 0.00 | 0.01 |
| *Synergistes* | 0.00 | 0.00 | 0.01 | 0.05 | 0.00 | 0.00 | 0.00 | 0.00 | 0.00 | 0.01 | 0.00 | 0.01 |
| *Anaeroplasma* | 0.00 | 0.00 | 0.00 | 0.00 | 0.00 | 0.00 | 0.00 | 0.00 | 0.05 | 0.16 | 0.00 | 0.00 |

Table S6. Average (± SD) relative abundance (%) of genes from predicted metagenomes of samples from vervets and humans consuming non-Western and Western diets. Western humans are from Italy (Schnorr et al. 2014) and the U.S. (Yatsunenko et al. 2012). Non-western humans are from Tanzania (Hadza, Schnorr et al. 2014), Venezuela (Guahibo, Yatsunenko et al. 2012), and Malawi (Yatsunenko et al. 2012). Bold text indicates significant differences between diet treatments (p < 0.05).

|  | Amato et al. vervets | | | | Schnorr et al. humans | | | | Yatsunenko et al. humans | | | |
| --- | --- | --- | --- | --- | --- | --- | --- | --- | --- | --- | --- | --- |
|  | non-Western | | Western | | non-Western | | Western | | non-Western | | Western | |
|  | Avg | SD | Avg | SD | Avg | SD | Avg | SD | Avg | SD | Avg | SD |
| K00001 | 0.038 | 0.025 | 0.030 | 0.020 | 0.011 | 0.009 | 0.027 | 0.016 | **0.015** | **0.009** | **0.028** | **0.016** |
| K00003 | 0.076 | 0.013 | 0.075 | 0.018 | 0.084 | 0.022 | 0.099 | 0.025 | **0.075** | **0.020** | **0.089** | **0.019** |
| K00008 | 0.077 | 0.043 | 0.042 | 0.023 | 0.080 | 0.024 | 0.096 | 0.019 | **0.071** | **0.019** | **0.101** | **0.027** |
| K00010 | 0.026 | 0.018 | 0.023 | 0.017 | 0.012 | 0.008 | 0.016 | 0.009 | **0.007** | **0.005** | **0.014** | **0.010** |
| K00013 | 0.066 | 0.013 | 0.065 | 0.011 | 0.066 | 0.013 | 0.074 | 0.011 | **0.060** | **0.016** | **0.077** | **0.015** |
| K00024 | 0.016 | 0.012 | 0.027 | 0.013 | 0.019 | 0.015 | 0.010 | 0.012 | **0.025** | **0.012** | **0.018** | **0.012** |
| K00029 | 0.020 | 0.017 | 0.038 | 0.019 | 0.008 | 0.009 | 0.006 | 0.008 | **0.021** | **0.011** | **0.008** | **0.008** |
| K00048 | 0.026 | 0.017 | 0.018 | 0.012 | 0.035 | 0.013 | 0.057 | 0.014 | **0.034** | **0.013** | **0.051** | **0.013** |
| K00068 | **0.009** | **0.009** | **0.001** | **0.002** | 0.004 | 0.004 | 0.010 | 0.006 | 0.004 | 0.004 | 0.008 | 0.007 |
| K00097 | 0.024 | 0.013 | 0.033 | 0.017 | 0.028 | 0.012 | 0.014 | 0.009 | **0.033** | **0.013** | **0.017** | **0.010** |
| K00098 | 0.001 | 0.004 | 0.001 | 0.002 | 0.000 | 0.001 | 0.000 | 0.000 | **0.002** | **0.003** | **0.000** | **0.001** |
| K00100 | 0.109 | 0.022 | 0.099 | 0.020 | 0.102 | 0.022 | 0.116 | 0.031 | **0.102** | **0.021** | **0.123** | **0.021** |
| K00145 | 0.139 | 0.023 | 0.126 | 0.022 | 0.144 | 0.024 | 0.141 | 0.015 | **0.130** | **0.019** | **0.147** | **0.022** |
| K00147 | 0.070 | 0.019 | 0.061 | 0.014 | 0.068 | 0.018 | 0.074 | 0.012 | **0.063** | **0.013** | **0.075** | **0.014** |
| K00163 | 0.005 | 0.008 | 0.007 | 0.013 | 0.000 | 0.001 | 0.000 | 0.000 | **0.002** | **0.003** | **0.000** | **0.001** |
| K00164 | 0.007 | 0.007 | 0.008 | 0.014 | 0.001 | 0.001 | 0.000 | 0.001 | **0.003** | **0.004** | **0.001** | **0.002** |
| K00174 | 0.039 | 0.018 | 0.043 | 0.024 | 0.042 | 0.024 | 0.018 | 0.016 | **0.048** | **0.018** | **0.035** | **0.018** |
| K00179 | 0.040 | 0.020 | 0.022 | 0.018 | 0.026 | 0.013 | 0.026 | 0.013 | **0.023** | **0.014** | **0.035** | **0.019** |
| K00180 | 0.043 | 0.023 | 0.025 | 0.019 | 0.033 | 0.016 | 0.022 | 0.009 | **0.024** | **0.015** | **0.036** | **0.019** |
| K00208 | 0.018 | 0.016 | 0.025 | 0.014 | 0.012 | 0.012 | 0.003 | 0.003 | **0.023** | **0.011** | **0.008** | **0.006** |
| K00231 | 0.006 | 0.006 | 0.015 | 0.011 | 0.006 | 0.009 | 0.001 | 0.001 | **0.016** | **0.010** | **0.001** | **0.003** |
| K00241 | 0.014 | 0.015 | 0.029 | 0.016 | 0.013 | 0.014 | 0.004 | 0.006 | **0.019** | **0.009** | **0.010** | **0.008** |
| K00245 | 0.007 | 0.013 | 0.004 | 0.009 | 0.000 | 0.001 | 0.000 | 0.000 | **0.003** | **0.004** | **0.001** | **0.002** |
| K00246 | 0.006 | 0.009 | 0.002 | 0.003 | 0.000 | 0.001 | 0.000 | 0.000 | **0.003** | **0.003** | **0.001** | **0.002** |
| K00247 | 0.003 | 0.004 | 0.001 | 0.002 | 0.000 | 0.001 | 0.000 | 0.000 | **0.002** | **0.003** | **0.000** | **0.000** |
| K00249 | 0.009 | 0.020 | 0.024 | 0.042 | 0.000 | 0.001 | 0.000 | 0.000 | **0.001** | **0.002** | **0.000** | **0.000** |
| K00257 | 0.021 | 0.028 | 0.049 | 0.049 | 0.020 | 0.013 | 0.006 | 0.006 | **0.024** | **0.011** | **0.011** | **0.009** |
| K00259 | 0.004 | 0.004 | 0.006 | 0.006 | 0.001 | 0.002 | 0.005 | 0.007 | **0.003** | **0.003** | **0.009** | **0.008** |
| K00278 | 0.058 | 0.028 | 0.045 | 0.016 | 0.057 | 0.014 | 0.067 | 0.016 | **0.049** | **0.014** | **0.065** | **0.016** |
| K00281 | 0.006 | 0.009 | 0.008 | 0.011 | 0.005 | 0.007 | 0.004 | 0.006 | **0.003** | **0.004** | **0.008** | **0.007** |
| K00282 | 0.010 | 0.008 | 0.020 | 0.014 | 0.020 | 0.011 | 0.009 | 0.007 | **0.026** | **0.013** | **0.006** | **0.006** |
| K00283 | 0.009 | 0.007 | 0.018 | 0.012 | 0.021 | 0.010 | 0.008 | 0.007 | **0.024** | **0.011** | **0.007** | **0.007** |
| K00325 | 0.007 | 0.006 | 0.007 | 0.012 | 0.000 | 0.001 | 0.003 | 0.004 | **0.004** | **0.004** | **0.001** | **0.003** |
| K00330 | 0.013 | 0.013 | 0.027 | 0.013 | 0.012 | 0.009 | 0.004 | 0.006 | **0.020** | **0.009** | **0.009** | **0.008** |
| K00337 | 0.013 | 0.011 | 0.025 | 0.013 | 0.012 | 0.008 | 0.004 | 0.004 | **0.021** | **0.012** | **0.010** | **0.008** |
| K00338 | 0.015 | 0.014 | 0.025 | 0.013 | 0.012 | 0.014 | 0.003 | 0.006 | **0.021** | **0.011** | **0.009** | **0.008** |
| K00339 | 0.015 | 0.014 | 0.026 | 0.014 | 0.012 | 0.011 | 0.005 | 0.009 | **0.021** | **0.013** | **0.009** | **0.008** |
| K00340 | 0.015 | 0.015 | 0.027 | 0.014 | 0.013 | 0.012 | 0.005 | 0.005 | **0.019** | **0.010** | **0.009** | **0.008** |
| K00341 | 0.014 | 0.012 | 0.024 | 0.013 | 0.013 | 0.012 | 0.004 | 0.006 | **0.022** | **0.010** | **0.009** | **0.007** |
| K00342 | 0.011 | 0.008 | 0.025 | 0.013 | 0.011 | 0.011 | 0.004 | 0.006 | **0.021** | **0.010** | **0.009** | **0.007** |
| K00343 | 0.015 | 0.012 | 0.026 | 0.013 | 0.013 | 0.011 | 0.004 | 0.007 | **0.023** | **0.010** | **0.008** | **0.007** |
| K00344 | 0.010 | 0.017 | 0.016 | 0.027 | 0.001 | 0.001 | 0.000 | 0.000 | **0.004** | **0.004** | **0.000** | **0.002** |
| K00346 | 0.006 | 0.006 | 0.015 | 0.011 | 0.015 | 0.012 | 0.005 | 0.007 | **0.021** | **0.013** | **0.008** | **0.007** |
| K00347 | 0.015 | 0.017 | 0.029 | 0.017 | 0.013 | 0.012 | 0.005 | 0.007 | **0.020** | **0.010** | **0.008** | **0.007** |
| K00348 | 0.015 | 0.013 | 0.030 | 0.016 | 0.013 | 0.013 | 0.003 | 0.005 | **0.021** | **0.012** | **0.008** | **0.007** |
| K00349 | 0.016 | 0.016 | 0.026 | 0.014 | 0.013 | 0.011 | 0.004 | 0.005 | **0.018** | **0.010** | **0.008** | **0.008** |
| K00350 | 0.017 | 0.016 | 0.032 | 0.020 | 0.016 | 0.011 | 0.005 | 0.004 | **0.021** | **0.012** | **0.009** | **0.008** |
| K00351 | 0.018 | 0.016 | 0.028 | 0.017 | 0.014 | 0.012 | 0.005 | 0.007 | **0.018** | **0.009** | **0.008** | **0.007** |
| K00366 | 0.003 | 0.005 | 0.003 | 0.004 | 0.007 | 0.007 | 0.007 | 0.007 | **0.005** | **0.004** | **0.011** | **0.007** |
| K00382 | 0.050 | 0.018 | 0.058 | 0.027 | 0.031 | 0.015 | 0.031 | 0.013 | **0.040** | **0.014** | **0.025** | **0.014** |
| K00383 | 0.008 | 0.015 | 0.006 | 0.010 | 0.001 | 0.002 | 0.000 | 0.000 | **0.004** | **0.004** | **0.000** | **0.001** |
| K00425 | 0.018 | 0.021 | 0.026 | 0.014 | 0.015 | 0.014 | 0.004 | 0.006 | **0.021** | **0.010** | **0.009** | **0.007** |
| K00426 | 0.014 | 0.012 | 0.029 | 0.015 | 0.013 | 0.012 | 0.004 | 0.006 | **0.023** | **0.012** | **0.009** | **0.007** |
| K00432 | **0.033** | **0.013** | **0.063** | **0.020** | **0.030** | **0.029** | **0.003** | **0.003** | **0.053** | **0.024** | **0.013** | **0.010** |
| K00450 | 0.001 | 0.003 | 0.002 | 0.004 | 0.002 | 0.004 | 0.001 | 0.002 | **0.001** | **0.002** | **0.003** | **0.003** |
| K00459 | 0.009 | 0.016 | 0.011 | 0.018 | 0.000 | 0.001 | 0.000 | 0.002 | **0.001** | **0.002** | **0.000** | **0.001** |
| K00517 | 0.003 | 0.003 | 0.006 | 0.009 | 0.000 | 0.001 | 0.000 | 0.000 | **0.001** | **0.002** | **0.000** | **0.000** |
| K00525 | 0.055 | 0.016 | 0.067 | 0.018 | 0.054 | 0.018 | 0.036 | 0.020 | **0.064** | **0.019** | **0.037** | **0.017** |
| K00526 | 0.034 | 0.019 | 0.039 | 0.017 | 0.017 | 0.010 | 0.015 | 0.010 | **0.030** | **0.014** | **0.015** | **0.009** |
| K00533 | 0.013 | 0.010 | 0.009 | 0.008 | 0.016 | 0.010 | 0.032 | 0.013 | **0.017** | **0.009** | **0.024** | **0.010** |
| K00557 | 0.006 | 0.009 | 0.003 | 0.006 | 0.001 | 0.001 | 0.000 | 0.000 | **0.003** | **0.004** | **0.000** | **0.001** |
| K00558 | 0.114 | 0.038 | 0.098 | 0.032 | 0.123 | 0.024 | 0.123 | 0.036 | **0.123** | **0.027** | **0.101** | **0.022** |
| K00564 | 0.008 | 0.006 | 0.011 | 0.009 | 0.008 | 0.007 | 0.004 | 0.006 | **0.008** | **0.006** | **0.003** | **0.003** |
| K00568 | 0.007 | 0.017 | 0.015 | 0.029 | 0.000 | 0.001 | 0.000 | 0.000 | **0.005** | **0.005** | **0.000** | **0.001** |
| K00595 | 0.044 | 0.027 | 0.049 | 0.019 | 0.083 | 0.021 | 0.100 | 0.021 | **0.065** | **0.019** | **0.084** | **0.020** |
| K00602 | 0.135 | 0.030 | 0.180 | 0.043 | 0.172 | 0.027 | 0.145 | 0.024 | **0.188** | **0.034** | **0.142** | **0.026** |
| K00605 | 0.015 | 0.013 | 0.029 | 0.016 | 0.015 | 0.012 | 0.008 | 0.008 | **0.023** | **0.012** | **0.011** | **0.009** |
| K00606 | 0.025 | 0.010 | 0.035 | 0.016 | 0.038 | 0.016 | 0.030 | 0.018 | **0.042** | **0.015** | **0.031** | **0.013** |
| K00616 | 0.060 | 0.020 | 0.052 | 0.015 | 0.048 | 0.014 | 0.063 | 0.015 | **0.049** | **0.015** | **0.064** | **0.015** |
| K00631 | 0.002 | 0.005 | 0.002 | 0.004 | 0.001 | 0.001 | 0.000 | 0.000 | **0.003** | **0.004** | **0.000** | **0.000** |
| K00634 | 0.010 | 0.008 | 0.013 | 0.010 | 0.016 | 0.012 | 0.006 | 0.005 | **0.016** | **0.009** | **0.010** | **0.007** |
| K00638 | 0.030 | 0.014 | 0.030 | 0.015 | 0.049 | 0.017 | 0.053 | 0.019 | **0.038** | **0.012** | **0.050** | **0.013** |
| K00645 | 0.075 | 0.021 | 0.080 | 0.019 | 0.085 | 0.016 | 0.072 | 0.009 | **0.086** | **0.016** | **0.073** | **0.017** |
| K00652 | 0.016 | 0.014 | 0.028 | 0.014 | 0.013 | 0.009 | 0.010 | 0.010 | **0.025** | **0.012** | **0.016** | **0.013** |
| K00656 | 0.110 | 0.036 | 0.094 | 0.037 | 0.116 | 0.025 | 0.123 | 0.025 | **0.106** | **0.021** | **0.133** | **0.028** |
| K00657 | 0.011 | 0.008 | 0.024 | 0.014 | 0.021 | 0.013 | 0.019 | 0.018 | **0.028** | **0.013** | **0.020** | **0.009** |
| K00661 | 0.041 | 0.015 | 0.037 | 0.018 | 0.052 | 0.015 | 0.063 | 0.016 | **0.047** | **0.013** | **0.063** | **0.018** |
| K00674 | 0.015 | 0.015 | 0.014 | 0.017 | 0.006 | 0.005 | 0.005 | 0.007 | **0.008** | **0.007** | **0.003** | **0.004** |
| K00676 | 0.028 | 0.016 | 0.013 | 0.007 | 0.020 | 0.011 | 0.022 | 0.018 | **0.019** | **0.010** | **0.028** | **0.012** |
| K00677 | 0.023 | 0.015 | 0.043 | 0.018 | 0.023 | 0.018 | 0.009 | 0.012 | **0.041** | **0.019** | **0.019** | **0.015** |
| K00697 | 0.002 | 0.006 | 0.003 | 0.006 | 0.000 | 0.001 | 0.003 | 0.004 | **0.000** | **0.001** | **0.004** | **0.005** |
| K00748 | 0.019 | 0.012 | 0.027 | 0.011 | 0.015 | 0.011 | 0.005 | 0.007 | **0.024** | **0.009** | **0.010** | **0.008** |
| K00754 | 0.044 | 0.018 | 0.035 | 0.014 | 0.050 | 0.012 | 0.067 | 0.023 | **0.047** | **0.017** | **0.063** | **0.019** |
| K00759 | 0.071 | 0.016 | 0.071 | 0.016 | 0.068 | 0.017 | 0.072 | 0.017 | **0.065** | **0.016** | **0.052** | **0.016** |
| K00765 | 0.068 | 0.012 | 0.059 | 0.015 | 0.063 | 0.017 | 0.063 | 0.018 | **0.056** | **0.017** | **0.070** | **0.016** |
| K00766 | 0.045 | 0.015 | 0.038 | 0.017 | 0.047 | 0.016 | 0.057 | 0.014 | **0.045** | **0.014** | **0.055** | **0.014** |
| K00767 | 0.045 | 0.017 | 0.046 | 0.015 | 0.058 | 0.014 | 0.059 | 0.011 | **0.048** | **0.013** | **0.064** | **0.015** |
| K00782 | 0.009 | 0.013 | 0.014 | 0.011 | 0.007 | 0.009 | 0.003 | 0.006 | **0.013** | **0.008** | **0.006** | **0.006** |
| K00791 | 0.079 | 0.017 | 0.092 | 0.024 | 0.093 | 0.025 | 0.076 | 0.023 | **0.099** | **0.019** | **0.081** | **0.016** |
| K00793 | 0.050 | 0.016 | 0.063 | 0.014 | 0.059 | 0.016 | 0.044 | 0.012 | **0.059** | **0.013** | **0.048** | **0.013** |
| K00794 | 0.055 | 0.014 | 0.072 | 0.014 | 0.063 | 0.014 | 0.050 | 0.015 | **0.062** | **0.013** | **0.053** | **0.015** |
| K00795 | 0.019 | 0.026 | 0.014 | 0.019 | 0.001 | 0.002 | 0.000 | 0.000 | **0.007** | **0.007** | **0.001** | **0.002** |
| K00799 | 0.021 | 0.044 | 0.051 | 0.097 | 0.001 | 0.002 | 0.000 | 0.000 | **0.006** | **0.007** | **0.000** | **0.002** |
| K00811 | 0.015 | 0.015 | 0.031 | 0.018 | 0.012 | 0.011 | 0.004 | 0.006 | **0.017** | **0.009** | **0.009** | **0.008** |
| K00812 | 0.020 | 0.014 | 0.030 | 0.014 | 0.019 | 0.012 | 0.013 | 0.008 | **0.027** | **0.010** | **0.014** | **0.008** |
| K00813 | 0.003 | 0.004 | 0.000 | 0.001 | 0.000 | 0.001 | 0.000 | 0.000 | **0.002** | **0.004** | **0.000** | **0.001** |
| K00819 | 0.000 | 0.001 | 0.001 | 0.003 | 0.000 | 0.000 | 0.001 | 0.003 | **0.000** | **0.001** | **0.002** | **0.003** |
| K00824 | 0.004 | 0.004 | 0.006 | 0.006 | 0.001 | 0.003 | 0.001 | 0.004 | **0.005** | **0.005** | **0.002** | **0.003** |
| K00836 | 0.000 | 0.002 | 0.003 | 0.006 | 0.003 | 0.006 | 0.000 | 0.000 | **0.001** | **0.001** | **0.000** | **0.000** |
| K00839 | **0.007** | **0.005** | **0.003** | **0.006** | 0.003 | 0.004 | 0.005 | 0.005 | 0.004 | 0.004 | 0.005 | 0.005 |
| K00844 | **0.008** | **0.009** | **0.000** | **0.001** | 0.004 | 0.005 | 0.006 | 0.005 | 0.003 | 0.005 | 0.004 | 0.005 |
| K00848 | 0.029 | 0.017 | 0.036 | 0.017 | 0.021 | 0.012 | 0.032 | 0.012 | **0.026** | **0.013** | **0.041** | **0.013** |
| K00850 | 0.146 | 0.044 | 0.145 | 0.051 | 0.216 | 0.033 | 0.237 | 0.027 | **0.191** | **0.028** | **0.219** | **0.030** |
| K00851 | 0.006 | 0.007 | 0.006 | 0.010 | 0.000 | 0.001 | 0.001 | 0.002 | **0.003** | **0.005** | **0.001** | **0.002** |
| K00852 | 0.063 | 0.024 | 0.047 | 0.014 | 0.045 | 0.018 | 0.051 | 0.026 | **0.045** | **0.013** | **0.062** | **0.019** |
| K00854 | 0.113 | 0.036 | 0.092 | 0.034 | 0.097 | 0.023 | 0.117 | 0.022 | **0.090** | **0.022** | **0.120** | **0.024** |
| K00857 | 0.031 | 0.017 | 0.042 | 0.018 | 0.024 | 0.012 | 0.020 | 0.008 | **0.030** | **0.012** | **0.021** | **0.011** |
| K00867 | 0.003 | 0.005 | 0.001 | 0.002 | 0.000 | 0.001 | 0.000 | 0.000 | **0.004** | **0.004** | **0.000** | **0.002** |
| K00874 | 0.088 | 0.022 | 0.079 | 0.028 | 0.084 | 0.017 | 0.096 | 0.020 | **0.078** | **0.019** | **0.093** | **0.019** |
| K00876 | 0.086 | 0.030 | 0.085 | 0.035 | 0.104 | 0.019 | 0.084 | 0.021 | **0.107** | **0.020** | **0.091** | **0.018** |
| K00879 | 0.013 | 0.012 | 0.002 | 0.003 | 0.007 | 0.007 | 0.011 | 0.010 | **0.007** | **0.006** | **0.014** | **0.011** |
| K00880 | 0.011 | 0.010 | 0.004 | 0.004 | 0.006 | 0.007 | 0.009 | 0.008 | **0.008** | **0.006** | **0.015** | **0.009** |
| K00895 | 0.015 | 0.016 | 0.029 | 0.017 | 0.010 | 0.013 | 0.003 | 0.006 | **0.018** | **0.010** | **0.008** | **0.008** |
| K00904 | 0.005 | 0.007 | 0.001 | 0.002 | 0.001 | 0.002 | 0.000 | 0.001 | **0.002** | **0.003** | **0.000** | **0.001** |
| K00912 | 0.017 | 0.013 | 0.028 | 0.012 | 0.017 | 0.014 | 0.005 | 0.006 | **0.027** | **0.013** | **0.010** | **0.008** |
| K00930 | 0.142 | 0.017 | 0.123 | 0.027 | 0.141 | 0.024 | 0.152 | 0.025 | **0.125** | **0.023** | **0.144** | **0.020** |
| K00931 | 0.076 | 0.019 | 0.060 | 0.019 | 0.065 | 0.016 | 0.067 | 0.015 | **0.061** | **0.014** | **0.077** | **0.017** |
| K00936 | 0.191 | 0.056 | 0.151 | 0.040 | 0.171 | 0.023 | 0.185 | 0.028 | **0.156** | **0.028** | **0.181** | **0.029** |
| K00937 | 0.057 | 0.016 | 0.052 | 0.018 | 0.069 | 0.021 | 0.079 | 0.030 | **0.068** | **0.016** | **0.086** | **0.019** |
| K00940 | 0.018 | 0.014 | 0.017 | 0.015 | 0.015 | 0.010 | 0.005 | 0.007 | **0.016** | **0.010** | **0.009** | **0.007** |
| K00946 | 0.020 | 0.016 | 0.028 | 0.015 | 0.020 | 0.016 | 0.005 | 0.006 | **0.025** | **0.012** | **0.012** | **0.010** |
| K00950 | 0.020 | 0.014 | 0.042 | 0.017 | 0.031 | 0.023 | 0.009 | 0.011 | **0.046** | **0.020** | **0.020** | **0.013** |
| K00951 | 0.075 | 0.018 | 0.091 | 0.024 | 0.083 | 0.022 | 0.073 | 0.024 | **0.096** | **0.020** | **0.080** | **0.019** |
| K00956 | 0.017 | 0.013 | 0.014 | 0.010 | 0.021 | 0.010 | 0.037 | 0.011 | **0.023** | **0.012** | **0.037** | **0.013** |
| K00957 | 0.015 | 0.012 | 0.018 | 0.009 | 0.022 | 0.010 | 0.039 | 0.018 | **0.025** | **0.012** | **0.039** | **0.015** |
| K00962 | 0.072 | 0.008 | 0.079 | 0.019 | 0.079 | 0.014 | 0.072 | 0.014 | **0.085** | **0.015** | **0.073** | **0.016** |
| K00971 | 0.026 | 0.012 | 0.044 | 0.021 | 0.028 | 0.018 | 0.018 | 0.019 | **0.041** | **0.018** | **0.019** | **0.013** |
| K00974 | 0.172 | 0.043 | 0.126 | 0.045 | 0.197 | 0.025 | 0.203 | 0.040 | **0.172** | **0.028** | **0.192** | **0.033** |
| K00975 | 0.123 | 0.034 | 0.103 | 0.040 | 0.158 | 0.032 | 0.187 | 0.028 | **0.147** | **0.033** | **0.176** | **0.036** |
| K00978 | 0.014 | 0.012 | 0.009 | 0.007 | 0.007 | 0.006 | 0.006 | 0.006 | **0.008** | **0.006** | **0.016** | **0.011** |
| K00980 | 0.019 | 0.012 | 0.017 | 0.010 | 0.025 | 0.013 | 0.033 | 0.016 | **0.022** | **0.010** | **0.033** | **0.014** |
| K00983 | 0.014 | 0.022 | 0.009 | 0.013 | 0.007 | 0.007 | 0.008 | 0.009 | **0.008** | **0.006** | **0.013** | **0.009** |
| K00989 | 0.021 | 0.011 | 0.015 | 0.011 | 0.014 | 0.008 | 0.012 | 0.009 | **0.014** | **0.007** | **0.008** | **0.007** |
| K00991 | 0.081 | 0.025 | 0.085 | 0.029 | 0.083 | 0.019 | 0.072 | 0.015 | **0.092** | **0.020** | **0.077** | **0.016** |
| K00995 | 0.106 | 0.025 | 0.083 | 0.016 | 0.104 | 0.024 | 0.112 | 0.023 | **0.093** | **0.018** | **0.108** | **0.021** |
| K01005 | 0.008 | 0.006 | 0.020 | 0.013 | 0.010 | 0.008 | 0.013 | 0.009 | **0.021** | **0.011** | **0.013** | **0.008** |
| K01041 | 0.001 | 0.002 | 0.003 | 0.004 | 0.000 | 0.001 | 0.004 | 0.007 | **0.001** | **0.002** | **0.006** | **0.006** |
| K01051 | 0.004 | 0.005 | 0.005 | 0.005 | 0.004 | 0.005 | 0.015 | 0.014 | **0.005** | **0.005** | **0.015** | **0.012** |
| K01057 | 0.005 | 0.006 | 0.004 | 0.007 | 0.000 | 0.001 | 0.006 | 0.006 | **0.003** | **0.005** | **0.008** | **0.007** |
| K01081 | **0.010** | **0.010** | **0.025** | **0.012** | 0.012 | 0.012 | 0.005 | 0.004 | **0.018** | **0.010** | **0.009** | **0.007** |
| K01112 | 0.003 | 0.006 | 0.001 | 0.003 | 0.000 | 0.001 | 0.000 | 0.000 | **0.002** | **0.004** | **0.000** | **0.000** |
| K01119 | 0.022 | 0.016 | 0.035 | 0.016 | 0.019 | 0.007 | 0.017 | 0.010 | **0.031** | **0.014** | **0.016** | **0.008** |
| K01126 | 0.060 | 0.015 | 0.057 | 0.019 | 0.058 | 0.015 | 0.072 | 0.024 | **0.055** | **0.015** | **0.069** | **0.019** |
| K01133 | 0.005 | 0.005 | 0.004 | 0.006 | 0.001 | 0.002 | 0.002 | 0.002 | **0.002** | **0.003** | **0.005** | **0.006** |
| K01135 | 0.000 | 0.000 | 0.000 | 0.000 | 0.000 | 0.000 | 0.001 | 0.002 | **0.000** | **0.000** | **0.001** | **0.002** |
| K01138 | 0.015 | 0.018 | 0.008 | 0.006 | 0.005 | 0.004 | 0.006 | 0.007 | **0.007** | **0.008** | **0.013** | **0.013** |
| K01141 | 0.003 | 0.006 | 0.005 | 0.008 | 0.000 | 0.001 | 0.000 | 0.001 | **0.003** | **0.005** | **0.001** | **0.001** |
| K01144 | 0.013 | 0.006 | 0.021 | 0.012 | 0.029 | 0.017 | 0.013 | 0.006 | **0.029** | **0.012** | **0.020** | **0.011** |
| K01147 | 0.004 | 0.004 | 0.003 | 0.005 | 0.001 | 0.003 | 0.000 | 0.001 | **0.003** | **0.004** | **0.001** | **0.001** |
| K01151 | 0.053 | 0.025 | 0.043 | 0.021 | 0.077 | 0.019 | 0.086 | 0.012 | **0.065** | **0.015** | **0.082** | **0.017** |
| K01159 | 0.056 | 0.018 | 0.053 | 0.017 | 0.045 | 0.015 | 0.030 | 0.017 | **0.043** | **0.014** | **0.033** | **0.015** |
| K01160 | 0.002 | 0.004 | 0.000 | 0.001 | 0.000 | 0.001 | 0.000 | 0.000 | **0.003** | **0.004** | **0.000** | **0.001** |
| K01176 | 0.031 | 0.020 | 0.031 | 0.018 | 0.013 | 0.011 | 0.014 | 0.009 | **0.025** | **0.012** | **0.015** | **0.009** |
| K01182 | 0.050 | 0.028 | 0.050 | 0.026 | 0.078 | 0.027 | 0.117 | 0.030 | **0.074** | **0.025** | **0.094** | **0.032** |
| K01185 | 0.014 | 0.013 | 0.025 | 0.015 | **0.018** | **0.014** | **0.003** | **0.004** | **0.038** | **0.017** | **0.006** | **0.008** |
| K01187 | 0.068 | 0.020 | 0.070 | 0.022 | 0.086 | 0.029 | 0.093 | 0.028 | **0.080** | **0.018** | **0.110** | **0.035** |
| K01188 | 0.055 | 0.020 | 0.055 | 0.022 | 0.056 | 0.015 | 0.065 | 0.022 | **0.049** | **0.016** | **0.064** | **0.019** |
| K01190 | 0.125 | 0.050 | 0.133 | 0.054 | 0.189 | 0.034 | 0.208 | 0.055 | **0.164** | **0.031** | **0.228** | **0.048** |
| K01192 | 0.031 | 0.014 | 0.032 | 0.017 | 0.033 | 0.014 | 0.035 | 0.020 | **0.021** | **0.011** | **0.031** | **0.012** |
| K01193 | 0.042 | 0.021 | 0.041 | 0.025 | 0.070 | 0.026 | 0.084 | 0.024 | **0.064** | **0.021** | **0.085** | **0.024** |
| K01201 | 0.009 | 0.009 | 0.003 | 0.003 | 0.011 | 0.007 | 0.023 | 0.018 | **0.008** | **0.007** | **0.020** | **0.015** |
| K01205 | 0.002 | 0.003 | 0.002 | 0.003 | 0.006 | 0.008 | 0.005 | 0.009 | **0.002** | **0.004** | **0.008** | **0.008** |
| K01209 | 0.083 | 0.031 | 0.056 | 0.020 | 0.071 | 0.017 | 0.109 | 0.045 | **0.068** | **0.020** | **0.101** | **0.025** |
| K01218 | 0.011 | 0.007 | 0.003 | 0.004 | 0.003 | 0.003 | 0.004 | 0.005 | **0.006** | **0.005** | **0.012** | **0.009** |
| K01235 | 0.002 | 0.004 | 0.003 | 0.004 | 0.000 | 0.001 | 0.002 | 0.003 | **0.001** | **0.002** | **0.004** | **0.004** |
| K01238 | 0.007 | 0.006 | 0.005 | 0.005 | 0.004 | 0.004 | 0.011 | 0.010 | **0.005** | **0.005** | **0.011** | **0.008** |
| K01241 | 0.004 | 0.006 | 0.007 | 0.009 | 0.001 | 0.002 | 0.004 | 0.006 | **0.002** | **0.002** | **0.007** | **0.007** |
| K01258 | 0.051 | 0.018 | 0.061 | 0.022 | 0.055 | 0.016 | 0.051 | 0.020 | **0.059** | **0.015** | **0.047** | **0.013** |
| K01270 | 0.052 | 0.018 | 0.076 | 0.029 | 0.069 | 0.021 | 0.060 | 0.018 | **0.078** | **0.018** | **0.064** | **0.016** |
| K01274 | 0.002 | 0.004 | 0.005 | 0.005 | 0.005 | 0.009 | 0.000 | 0.001 | **0.013** | **0.008** | **0.001** | **0.004** |
| K01277 | 0.004 | 0.006 | 0.014 | 0.009 | 0.014 | 0.012 | 0.003 | 0.006 | **0.017** | **0.010** | **0.008** | **0.007** |
| K01278 | 0.010 | 0.012 | 0.032 | 0.020 | 0.020 | 0.019 | 0.013 | 0.016 | **0.034** | **0.018** | **0.019** | **0.015** |
| K01281 | 0.002 | 0.006 | 0.000 | 0.001 | 0.000 | 0.001 | 0.000 | 0.000 | **0.001** | **0.003** | **0.000** | **0.001** |
| K01284 | 0.009 | 0.010 | 0.028 | 0.017 | 0.021 | 0.021 | 0.009 | 0.012 | **0.033** | **0.013** | **0.019** | **0.015** |
| K01322 | 0.004 | 0.005 | 0.009 | 0.007 | **0.008** | **0.007** | **0.000** | **0.001** | **0.014** | **0.009** | **0.004** | **0.005** |
| K01362 | 0.065 | 0.029 | 0.067 | 0.021 | 0.058 | 0.017 | 0.028 | 0.016 | **0.054** | **0.020** | **0.038** | **0.017** |
| K01372 | 0.011 | 0.011 | 0.031 | 0.022 | 0.030 | 0.022 | 0.015 | 0.013 | **0.039** | **0.017** | **0.018** | **0.013** |
| K01414 | 0.004 | 0.007 | 0.004 | 0.008 | 0.001 | 0.002 | 0.000 | 0.000 | **0.003** | **0.005** | **0.001** | **0.002** |
| K01417 | 0.033 | 0.039 | 0.023 | 0.022 | 0.024 | 0.010 | 0.015 | 0.008 | **0.021** | **0.009** | **0.012** | **0.007** |
| K01419 | 0.021 | 0.023 | 0.023 | 0.024 | 0.003 | 0.006 | 0.000 | 0.001 | **0.007** | **0.007** | **0.001** | **0.002** |
| K01420 | 0.064 | 0.022 | 0.047 | 0.016 | 0.073 | 0.014 | 0.083 | 0.015 | **0.062** | **0.015** | **0.073** | **0.017** |
| K01421 | 0.048 | 0.020 | 0.038 | 0.022 | 0.069 | 0.021 | 0.099 | 0.030 | **0.068** | **0.021** | **0.089** | **0.024** |
| K01424 | 0.045 | 0.021 | 0.058 | 0.022 | 0.057 | 0.021 | 0.035 | 0.013 | **0.065** | **0.017** | **0.045** | **0.015** |
| K01425 | 0.013 | 0.008 | 0.008 | 0.005 | 0.015 | 0.011 | 0.015 | 0.010 | **0.009** | **0.006** | **0.016** | **0.010** |
| K01442 | 0.040 | 0.023 | 0.025 | 0.014 | 0.043 | 0.014 | 0.067 | 0.017 | **0.044** | **0.015** | **0.069** | **0.014** |
| K01443 | 0.057 | 0.017 | 0.054 | 0.018 | 0.068 | 0.015 | 0.080 | 0.018 | **0.060** | **0.016** | **0.072** | **0.016** |
| K01447 | 0.020 | 0.009 | 0.013 | 0.009 | 0.019 | 0.015 | 0.029 | 0.018 | **0.015** | **0.012** | **0.032** | **0.017** |
| K01464 | 0.040 | 0.014 | 0.041 | 0.017 | 0.039 | 0.014 | 0.038 | 0.015 | **0.035** | **0.012** | **0.045** | **0.013** |
| K01465 | 0.084 | 0.021 | 0.088 | 0.013 | 0.078 | 0.018 | 0.079 | 0.016 | **0.082** | **0.017** | **0.070** | **0.017** |
| K01468 | 0.006 | 0.010 | 0.008 | 0.012 | 0.001 | 0.002 | 0.004 | 0.005 | **0.001** | **0.002** | **0.007** | **0.007** |
| K01492 | 0.007 | 0.008 | 0.001 | 0.001 | 0.002 | 0.003 | 0.010 | 0.009 | **0.002** | **0.004** | **0.008** | **0.008** |
| K01494 | 0.020 | 0.019 | 0.025 | 0.018 | 0.001 | 0.002 | 0.005 | 0.006 | **0.008** | **0.007** | **0.003** | **0.004** |
| K01500 | 0.002 | 0.003 | 0.002 | 0.003 | 0.007 | 0.005 | 0.002 | 0.003 | **0.004** | **0.004** | **0.002** | **0.002** |
| K01525 | 0.004 | 0.006 | 0.004 | 0.008 | 0.000 | 0.001 | 0.000 | 0.000 | **0.003** | **0.004** | **0.001** | **0.001** |
| K01546 | 0.021 | 0.014 | 0.013 | 0.010 | 0.012 | 0.008 | 0.019 | 0.016 | **0.010** | **0.007** | **0.017** | **0.010** |
| K01547 | 0.020 | 0.015 | 0.012 | 0.008 | 0.014 | 0.008 | 0.015 | 0.012 | **0.009** | **0.007** | **0.019** | **0.012** |
| K01548 | 0.010 | 0.009 | 0.012 | 0.008 | 0.011 | 0.009 | 0.010 | 0.008 | **0.007** | **0.006** | **0.015** | **0.009** |
| K01552 | 0.027 | 0.014 | 0.027 | 0.013 | 0.032 | 0.015 | 0.056 | 0.016 | **0.036** | **0.014** | **0.050** | **0.014** |
| K01567 | 0.003 | 0.005 | 0.001 | 0.003 | 0.000 | 0.000 | 0.000 | 0.001 | **0.000** | **0.001** | **0.002** | **0.005** |
| K01572 | 0.068 | 0.024 | 0.054 | 0.022 | 0.063 | 0.014 | 0.074 | 0.019 | **0.055** | **0.015** | **0.073** | **0.020** |
| K01573 | 0.009 | 0.014 | 0.016 | 0.021 | 0.000 | 0.001 | 0.006 | 0.008 | **0.001** | **0.002** | **0.007** | **0.007** |
| K01580 | 0.003 | 0.005 | 0.004 | 0.003 | 0.005 | 0.006 | 0.006 | 0.007 | **0.003** | **0.005** | **0.009** | **0.007** |
| K01581 | 0.006 | 0.005 | 0.007 | 0.006 | 0.005 | 0.007 | 0.001 | 0.002 | **0.011** | **0.010** | **0.004** | **0.006** |
| K01585 | 0.021 | 0.019 | 0.035 | 0.017 | 0.013 | 0.010 | 0.006 | 0.006 | **0.021** | **0.012** | **0.009** | **0.008** |
| K01601 | 0.003 | 0.006 | 0.004 | 0.009 | 0.000 | 0.000 | 0.000 | 0.000 | **0.003** | **0.004** | **0.001** | **0.002** |
| K01607 | 0.042 | 0.016 | 0.042 | 0.017 | 0.055 | 0.015 | 0.060 | 0.022 | **0.040** | **0.013** | **0.055** | **0.015** |
| K01624 | 0.136 | 0.032 | 0.132 | 0.035 | 0.130 | 0.022 | 0.150 | 0.029 | **0.124** | **0.021** | **0.148** | **0.024** |
| K01627 | 0.018 | 0.012 | 0.029 | 0.014 | 0.012 | 0.011 | 0.005 | 0.007 | **0.024** | **0.011** | **0.011** | **0.008** |
| K01629 | 0.008 | 0.008 | 0.013 | 0.010 | 0.014 | 0.008 | 0.026 | 0.016 | **0.016** | **0.011** | **0.023** | **0.011** |
| K01633 | 0.023 | 0.016 | 0.030 | 0.016 | **0.021** | **0.015** | **0.005** | **0.005** | **0.029** | **0.013** | **0.012** | **0.009** |
| K01644 | 0.031 | 0.019 | 0.044 | 0.035 | 0.006 | 0.004 | 0.003 | 0.004 | **0.012** | **0.007** | **0.006** | **0.005** |
| K01646 | 0.001 | 0.001 | 0.004 | 0.004 | 0.002 | 0.003 | 0.001 | 0.001 | **0.003** | **0.003** | **0.001** | **0.002** |
| K01647 | 0.102 | 0.017 | 0.079 | 0.015 | 0.101 | 0.022 | 0.109 | 0.025 | **0.089** | **0.021** | **0.106** | **0.021** |
| K01649 | 0.099 | 0.030 | 0.081 | 0.027 | 0.083 | 0.018 | 0.081 | 0.014 | **0.070** | **0.015** | **0.082** | **0.016** |
| K01652 | 0.130 | 0.026 | 0.102 | 0.020 | 0.132 | 0.027 | 0.152 | 0.013 | **0.119** | **0.024** | **0.140** | **0.024** |
| K01653 | 0.087 | 0.019 | 0.064 | 0.017 | 0.071 | 0.017 | 0.079 | 0.015 | **0.067** | **0.016** | **0.080** | **0.018** |
| K01654 | 0.023 | 0.026 | 0.023 | 0.018 | 0.007 | 0.007 | 0.014 | 0.011 | **0.009** | **0.007** | **0.015** | **0.009** |
| K01657 | 0.061 | 0.014 | 0.052 | 0.013 | 0.050 | 0.013 | 0.058 | 0.016 | **0.045** | **0.012** | **0.056** | **0.014** |
| K01661 | 0.009 | 0.009 | 0.016 | 0.009 | 0.008 | 0.009 | 0.004 | 0.006 | **0.018** | **0.010** | **0.007** | **0.006** |
| K01665 | 0.008 | 0.008 | 0.022 | 0.011 | 0.015 | 0.012 | 0.008 | 0.010 | **0.023** | **0.013** | **0.012** | **0.008** |
| K01666 | 0.016 | 0.013 | 0.010 | 0.007 | 0.016 | 0.011 | 0.021 | 0.013 | **0.018** | **0.008** | **0.030** | **0.013** |
| K01676 | 0.008 | 0.007 | 0.020 | 0.012 | 0.012 | 0.010 | 0.003 | 0.004 | **0.018** | **0.009** | **0.009** | **0.009** |
| K01679 | 0.015 | 0.014 | 0.016 | 0.015 | 0.006 | 0.005 | 0.003 | 0.005 | **0.010** | **0.007** | **0.005** | **0.004** |
| K01682 | 0.009 | 0.018 | 0.007 | 0.012 | 0.001 | 0.002 | 0.000 | 0.000 | **0.002** | **0.004** | **0.000** | **0.000** |
| K01687 | 0.093 | 0.016 | 0.085 | 0.014 | 0.096 | 0.021 | 0.108 | 0.025 | **0.090** | **0.022** | **0.104** | **0.017** |
| K01703 | 0.156 | 0.022 | 0.148 | 0.028 | 0.170 | 0.025 | 0.175 | 0.032 | **0.148** | **0.024** | **0.174** | **0.027** |
| K01704 | 0.154 | 0.020 | 0.140 | 0.027 | 0.163 | 0.030 | 0.180 | 0.029 | **0.151** | **0.027** | **0.169** | **0.024** |
| K01709 | 0.011 | 0.007 | 0.008 | 0.009 | 0.007 | 0.007 | 0.007 | 0.006 | **0.008** | **0.007** | **0.016** | **0.011** |
| K01712 | 0.003 | 0.004 | 0.009 | 0.012 | 0.002 | 0.004 | 0.004 | 0.005 | **0.002** | **0.003** | **0.007** | **0.007** |
| K01716 | 0.003 | 0.004 | 0.003 | 0.006 | 0.000 | 0.001 | 0.000 | 0.000 | **0.003** | **0.004** | **0.000** | **0.000** |
| K01719 | 0.013 | 0.014 | 0.025 | 0.013 | 0.014 | 0.011 | 0.004 | 0.007 | **0.020** | **0.011** | **0.009** | **0.008** |
| K01730 | 0.002 | 0.003 | 0.001 | 0.002 | 0.001 | 0.001 | 0.001 | 0.003 | **0.001** | **0.001** | **0.002** | **0.003** |
| K01734 | 0.057 | 0.026 | 0.046 | 0.024 | 0.081 | 0.020 | 0.094 | 0.016 | **0.074** | **0.020** | **0.098** | **0.021** |
| K01744 | 0.019 | 0.012 | 0.029 | 0.016 | 0.028 | 0.014 | 0.018 | 0.010 | **0.034** | **0.011** | **0.022** | **0.010** |
| K01745 | 0.005 | 0.007 | 0.017 | 0.014 | 0.004 | 0.004 | 0.005 | 0.006 | **0.005** | **0.004** | **0.012** | **0.011** |
| K01750 | 0.008 | 0.013 | 0.013 | 0.019 | 0.001 | 0.003 | 0.003 | 0.003 | **0.001** | **0.002** | **0.004** | **0.005** |
| K01771 | 0.000 | 0.000 | 0.000 | 0.002 | 0.000 | 0.000 | 0.001 | 0.002 | **0.000** | **0.000** | **0.001** | **0.003** |
| K01772 | 0.020 | 0.024 | 0.014 | 0.018 | 0.001 | 0.001 | 0.000 | 0.001 | **0.005** | **0.005** | **0.001** | **0.002** |
| K01782 | 0.007 | 0.009 | 0.012 | 0.018 | 0.002 | 0.003 | 0.000 | 0.002 | **0.004** | **0.005** | **0.002** | **0.005** |
| K01785 | 0.080 | 0.026 | 0.073 | 0.023 | 0.085 | 0.014 | 0.087 | 0.021 | **0.083** | **0.019** | **0.100** | **0.023** |
| K01786 | 0.046 | 0.020 | 0.035 | 0.018 | 0.036 | 0.010 | 0.051 | 0.013 | **0.039** | **0.012** | **0.056** | **0.016** |
| K01795 | 0.019 | 0.015 | 0.022 | 0.017 | 0.008 | 0.006 | 0.009 | 0.006 | **0.007** | **0.005** | **0.014** | **0.008** |
| K01802 | **0.024** | **0.019** | **0.053** | **0.015** | 0.026 | 0.018 | 0.015 | 0.011 | **0.037** | **0.018** | **0.020** | **0.014** |
| K01804 | 0.027 | 0.011 | 0.037 | 0.017 | 0.025 | 0.015 | 0.046 | 0.021 | **0.030** | **0.013** | **0.043** | **0.014** |
| K01805 | 0.022 | 0.013 | 0.021 | 0.018 | 0.010 | 0.007 | 0.010 | 0.011 | **0.008** | **0.007** | **0.014** | **0.009** |
| K01811 | 0.065 | 0.025 | 0.058 | 0.023 | 0.054 | 0.014 | 0.066 | 0.017 | **0.049** | **0.016** | **0.080** | **0.025** |
| K01813 | 0.013 | 0.009 | 0.008 | 0.007 | 0.008 | 0.005 | 0.019 | 0.012 | **0.010** | **0.007** | **0.018** | **0.011** |
| K01818 | 0.018 | 0.013 | 0.010 | 0.008 | 0.018 | 0.012 | 0.025 | 0.015 | **0.018** | **0.010** | **0.028** | **0.012** |
| K01821 | 0.011 | 0.024 | 0.009 | 0.014 | 0.001 | 0.002 | 0.000 | 0.002 | **0.003** | **0.004** | **0.001** | **0.001** |
| K01825 | 0.005 | 0.009 | 0.006 | 0.012 | 0.000 | 0.001 | 0.000 | 0.001 | **0.003** | **0.005** | **0.000** | **0.001** |
| K01840 | 0.105 | 0.025 | 0.111 | 0.031 | 0.108 | 0.021 | 0.098 | 0.019 | **0.113** | **0.018** | **0.097** | **0.017** |
| K01843 | 0.013 | 0.012 | 0.017 | 0.017 | 0.002 | 0.002 | 0.007 | 0.007 | **0.003** | **0.004** | **0.008** | **0.007** |
| K01847 | 0.010 | 0.009 | 0.012 | 0.009 | 0.013 | 0.015 | 0.009 | 0.010 | **0.009** | **0.010** | **0.017** | **0.013** |
| K01850 | 0.002 | 0.003 | 0.004 | 0.005 | 0.002 | 0.002 | 0.004 | 0.009 | **0.002** | **0.003** | **0.007** | **0.006** |
| K01858 | **0.003** | **0.005** | **0.016** | **0.011** | 0.008 | 0.009 | 0.006 | 0.005 | **0.016** | **0.011** | **0.009** | **0.006** |
| K01878 | 0.011 | 0.015 | 0.010 | 0.011 | 0.002 | 0.003 | 0.000 | 0.001 | **0.010** | **0.006** | **0.002** | **0.003** |
| K01879 | 0.012 | 0.017 | 0.012 | 0.014 | 0.002 | 0.002 | 0.001 | 0.002 | **0.009** | **0.006** | **0.002** | **0.003** |
| K01902 | 0.013 | 0.015 | 0.022 | 0.014 | 0.008 | 0.013 | 0.004 | 0.005 | **0.019** | **0.010** | **0.006** | **0.005** |
| K01903 | 0.013 | 0.010 | 0.021 | 0.014 | 0.008 | 0.010 | 0.005 | 0.005 | **0.018** | **0.010** | **0.006** | **0.006** |
| K01911 | 0.008 | 0.006 | 0.016 | 0.008 | 0.009 | 0.010 | 0.005 | 0.008 | **0.020** | **0.012** | **0.010** | **0.008** |
| K01912 | 0.068 | 0.033 | 0.055 | 0.028 | 0.064 | 0.020 | 0.062 | 0.027 | **0.054** | **0.020** | **0.077** | **0.025** |
| K01917 | 0.004 | 0.013 | 0.003 | 0.007 | 0.000 | 0.001 | 0.002 | 0.005 | **0.000** | **0.001** | **0.002** | **0.003** |
| K01920 | 0.005 | 0.008 | 0.005 | 0.010 | 0.000 | 0.001 | 0.000 | 0.001 | **0.003** | **0.005** | **0.000** | **0.001** |
| K01923 | 0.071 | 0.012 | 0.076 | 0.021 | 0.081 | 0.019 | 0.075 | 0.013 | **0.080** | **0.015** | **0.069** | **0.015** |
| K01926 | 0.065 | 0.026 | 0.059 | 0.028 | 0.060 | 0.021 | 0.054 | 0.017 | **0.043** | **0.016** | **0.053** | **0.014** |
| K01953 | 0.090 | 0.029 | 0.065 | 0.029 | 0.091 | 0.022 | 0.102 | 0.027 | **0.078** | **0.017** | **0.099** | **0.018** |
| K01961 | 0.122 | 0.025 | 0.097 | 0.023 | 0.101 | 0.029 | 0.121 | 0.028 | **0.103** | **0.026** | **0.129** | **0.028** |
| K01966 | 0.006 | 0.007 | 0.016 | 0.012 | 0.007 | 0.009 | 0.001 | 0.002 | **0.016** | **0.010** | **0.004** | **0.005** |
| K01993 | 0.019 | 0.011 | 0.042 | 0.020 | 0.024 | 0.019 | 0.012 | 0.016 | **0.043** | **0.019** | **0.024** | **0.019** |
| K02001 | 0.005 | 0.006 | 0.007 | 0.011 | 0.001 | 0.002 | 0.002 | 0.003 | **0.002** | **0.003** | **0.005** | **0.006** |
| K02003 | 0.481 | 0.144 | 0.404 | 0.124 | 0.575 | 0.072 | 0.665 | 0.058 | **0.509** | **0.067** | **0.635** | **0.069** |
| K02004 | 0.379 | 0.079 | 0.322 | 0.083 | 0.421 | 0.054 | 0.468 | 0.031 | **0.381** | **0.053** | **0.502** | **0.050** |
| K02005 | 0.043 | 0.018 | 0.049 | 0.016 | 0.058 | 0.020 | 0.066 | 0.020 | **0.052** | **0.016** | **0.069** | **0.026** |
| K02006 | 0.240 | 0.056 | 0.191 | 0.064 | 0.258 | 0.042 | 0.269 | 0.035 | **0.226** | **0.032** | **0.253** | **0.038** |
| K02010 | 0.046 | 0.018 | 0.041 | 0.020 | 0.021 | 0.008 | 0.012 | 0.008 | **0.021** | **0.010** | **0.013** | **0.008** |
| K02011 | 0.052 | 0.020 | 0.061 | 0.029 | 0.035 | 0.011 | 0.019 | 0.009 | **0.031** | **0.013** | **0.019** | **0.010** |
| K02012 | 0.057 | 0.024 | 0.067 | 0.035 | 0.035 | 0.013 | 0.024 | 0.013 | **0.036** | **0.013** | **0.022** | **0.011** |
| K02014 | 0.100 | 0.078 | 0.221 | 0.145 | 0.072 | 0.063 | 0.032 | 0.044 | **0.128** | **0.049** | **0.068** | **0.042** |
| K02025 | 0.647 | 0.206 | 0.521 | 0.206 | 0.596 | 0.127 | 0.723 | 0.119 | **0.514** | **0.091** | **0.634** | **0.091** |
| K02026 | 0.641 | 0.204 | 0.506 | 0.200 | 0.560 | 0.110 | 0.697 | 0.124 | **0.487** | **0.083** | **0.615** | **0.090** |
| K02027 | 0.387 | 0.115 | 0.365 | 0.153 | 0.373 | 0.080 | 0.437 | 0.068 | **0.309** | **0.059** | **0.381** | **0.064** |
| K02039 | 0.073 | 0.024 | 0.063 | 0.015 | 0.069 | 0.016 | 0.070 | 0.020 | **0.062** | **0.014** | **0.076** | **0.017** |
| K02062 | 0.002 | 0.004 | 0.001 | 0.002 | 0.001 | 0.002 | 0.000 | 0.000 | **0.003** | **0.004** | **0.000** | **0.000** |
| K02063 | 0.002 | 0.004 | 0.001 | 0.002 | 0.002 | 0.004 | 0.000 | 0.000 | **0.003** | **0.004** | **0.000** | **0.001** |
| K02064 | 0.003 | 0.005 | 0.001 | 0.002 | 0.002 | 0.004 | 0.000 | 0.000 | **0.003** | **0.004** | **0.000** | **0.000** |
| K02065 | 0.027 | 0.022 | 0.046 | 0.017 | 0.013 | 0.013 | 0.006 | 0.007 | **0.024** | **0.011** | **0.013** | **0.009** |
| K02066 | 0.024 | 0.019 | 0.050 | 0.017 | 0.017 | 0.012 | 0.005 | 0.007 | **0.025** | **0.012** | **0.011** | **0.010** |
| K02067 | 0.019 | 0.019 | 0.027 | 0.021 | 0.002 | 0.003 | 0.000 | 0.000 | **0.007** | **0.007** | **0.002** | **0.003** |
| K02069 | 0.005 | 0.004 | 0.005 | 0.004 | 0.001 | 0.003 | 0.006 | 0.006 | **0.003** | **0.004** | **0.008** | **0.006** |
| K02074 | 0.002 | 0.003 | 0.002 | 0.004 | **0.000** | **0.000** | **0.004** | **0.004** | 0.001 | 0.003 | 0.002 | 0.003 |
| K02075 | 0.002 | 0.003 | 0.002 | 0.005 | **0.000** | **0.000** | **0.005** | **0.007** | 0.001 | 0.004 | 0.002 | 0.003 |
| K02077 | 0.006 | 0.007 | 0.004 | 0.005 | 0.004 | 0.007 | 0.015 | 0.014 | **0.005** | **0.007** | **0.011** | **0.007** |
| K02081 | 0.015 | 0.011 | 0.027 | 0.015 | 0.010 | 0.008 | 0.004 | 0.003 | **0.017** | **0.008** | **0.007** | **0.007** |
| K02103 | 0.031 | 0.020 | 0.025 | 0.017 | 0.020 | 0.011 | 0.036 | 0.014 | **0.020** | **0.009** | **0.031** | **0.012** |
| K02117 | 0.058 | 0.018 | 0.049 | 0.024 | 0.068 | 0.016 | 0.056 | 0.016 | **0.045** | **0.015** | **0.062** | **0.015** |
| K02118 | 0.058 | 0.021 | 0.050 | 0.022 | 0.067 | 0.019 | 0.065 | 0.015 | **0.051** | **0.016** | **0.071** | **0.017** |
| K02120 | 0.060 | 0.020 | 0.045 | 0.019 | 0.062 | 0.014 | 0.062 | 0.014 | **0.050** | **0.015** | **0.068** | **0.016** |
| K02121 | 0.057 | 0.019 | 0.048 | 0.021 | 0.058 | 0.015 | 0.059 | 0.019 | **0.046** | **0.016** | **0.060** | **0.016** |
| K02122 | 0.051 | 0.023 | 0.034 | 0.018 | 0.062 | 0.017 | 0.066 | 0.023 | **0.049** | **0.014** | **0.061** | **0.017** |
| K02123 | 0.053 | 0.018 | 0.049 | 0.022 | 0.059 | 0.016 | 0.063 | 0.017 | **0.048** | **0.015** | **0.061** | **0.015** |
| K02124 | 0.058 | 0.021 | 0.050 | 0.022 | 0.067 | 0.021 | 0.066 | 0.021 | **0.054** | **0.016** | **0.072** | **0.015** |
| K02168 | 0.003 | 0.006 | 0.004 | 0.007 | 0.001 | 0.001 | 0.000 | 0.000 | **0.002** | **0.004** | **0.000** | **0.001** |
| K02170 | 0.002 | 0.006 | 0.002 | 0.004 | 0.000 | 0.001 | 0.000 | 0.000 | **0.003** | **0.004** | **0.000** | **0.000** |
| K02190 | 0.021 | 0.012 | 0.019 | 0.012 | 0.025 | 0.012 | 0.034 | 0.019 | **0.027** | **0.011** | **0.036** | **0.014** |
| K02199 | 0.003 | 0.005 | 0.004 | 0.008 | 0.000 | 0.001 | 0.000 | 0.000 | **0.003** | **0.004** | **0.000** | **0.001** |
| K02200 | 0.006 | 0.009 | 0.010 | 0.018 | 0.001 | 0.002 | 0.000 | 0.002 | **0.004** | **0.004** | **0.001** | **0.003** |
| K02217 | 0.050 | 0.021 | 0.039 | 0.020 | 0.039 | 0.013 | 0.028 | 0.014 | **0.039** | **0.012** | **0.031** | **0.014** |
| K02221 | 0.021 | 0.021 | 0.024 | 0.016 | 0.010 | 0.007 | 0.006 | 0.006 | **0.009** | **0.007** | **0.004** | **0.005** |
| K02224 | 0.062 | 0.022 | 0.059 | 0.020 | 0.086 | 0.022 | 0.111 | 0.023 | **0.078** | **0.020** | **0.098** | **0.022** |
| K02242 | 0.006 | 0.011 | 0.003 | 0.007 | 0.001 | 0.002 | 0.000 | 0.001 | **0.002** | **0.004** | **0.000** | **0.002** |
| K02276 | 0.004 | 0.006 | 0.007 | 0.015 | 0.000 | 0.001 | 0.000 | 0.001 | **0.002** | **0.002** | **0.000** | **0.001** |
| K02297 | 0.002 | 0.003 | 0.003 | 0.005 | 0.000 | 0.001 | 0.000 | 0.000 | **0.002** | **0.004** | **0.000** | **0.000** |
| K02298 | 0.003 | 0.004 | 0.002 | 0.004 | 0.001 | 0.002 | 0.000 | 0.000 | **0.003** | **0.005** | **0.000** | **0.000** |
| K02299 | 0.004 | 0.007 | 0.002 | 0.004 | 0.000 | 0.001 | 0.000 | 0.001 | **0.002** | **0.003** | **0.000** | **0.001** |
| K02300 | 0.004 | 0.007 | 0.003 | 0.005 | 0.000 | 0.001 | 0.000 | 0.000 | **0.003** | **0.004** | **0.000** | **0.000** |
| K02301 | 0.006 | 0.007 | 0.007 | 0.012 | 0.000 | 0.001 | 0.000 | 0.002 | **0.004** | **0.004** | **0.001** | **0.002** |
| K02315 | 0.081 | 0.025 | 0.068 | 0.028 | 0.104 | 0.032 | 0.126 | 0.021 | **0.093** | **0.021** | **0.109** | **0.022** |
| K02339 | 0.004 | 0.006 | 0.006 | 0.011 | 0.001 | 0.002 | 0.000 | 0.000 | **0.003** | **0.004** | **0.000** | **0.001** |
| K02342 | 0.058 | 0.023 | 0.073 | 0.020 | 0.037 | 0.024 | 0.027 | 0.013 | **0.059** | **0.020** | **0.036** | **0.016** |
| K02343 | 0.081 | 0.026 | 0.085 | 0.025 | 0.074 | 0.022 | 0.068 | 0.019 | **0.074** | **0.015** | **0.063** | **0.014** |
| K02344 | 0.004 | 0.006 | 0.001 | 0.001 | 0.000 | 0.001 | 0.000 | 0.000 | **0.003** | **0.004** | **0.000** | **0.000** |
| K02361 | 0.006 | 0.010 | 0.015 | 0.010 | 0.007 | 0.010 | 0.004 | 0.007 | **0.015** | **0.010** | **0.005** | **0.005** |
| K02371 | 0.039 | 0.016 | 0.027 | 0.013 | 0.046 | 0.012 | 0.048 | 0.021 | **0.044** | **0.013** | **0.054** | **0.014** |
| K02383 | 0.011 | 0.016 | 0.012 | 0.016 | 0.002 | 0.004 | 0.000 | 0.000 | **0.001** | **0.002** | **0.000** | **0.000** |
| K02394 | 0.007 | 0.013 | 0.009 | 0.014 | 0.001 | 0.001 | 0.000 | 0.000 | **0.002** | **0.003** | **0.000** | **0.001** |
| K02411 | 0.023 | 0.018 | 0.024 | 0.015 | 0.012 | 0.009 | 0.002 | 0.002 | **0.007** | **0.005** | **0.004** | **0.005** |
| K02415 | 0.018 | 0.018 | 0.026 | 0.021 | 0.003 | 0.006 | 0.000 | 0.001 | **0.003** | **0.004** | **0.001** | **0.002** |
| K02426 | 0.008 | 0.006 | 0.020 | 0.011 | 0.014 | 0.013 | 0.004 | 0.005 | **0.021** | **0.011** | **0.010** | **0.008** |
| K02427 | 0.016 | 0.016 | 0.019 | 0.017 | 0.002 | 0.004 | 0.000 | 0.001 | **0.004** | **0.005** | **0.001** | **0.002** |
| K02428 | 0.021 | 0.015 | 0.034 | 0.016 | 0.017 | 0.014 | 0.008 | 0.010 | **0.022** | **0.010** | **0.010** | **0.008** |
| K02429 | **0.009** | **0.007** | **0.031** | **0.019** | 0.022 | 0.018 | 0.012 | 0.017 | 0.023 | 0.012 | 0.023 | 0.017 |
| K02431 | 0.025 | 0.014 | 0.030 | 0.021 | 0.017 | 0.008 | 0.021 | 0.018 | **0.019** | **0.010** | **0.028** | **0.013** |
| K02437 | 0.023 | 0.016 | 0.042 | 0.019 | 0.016 | 0.011 | 0.010 | 0.010 | **0.023** | **0.010** | **0.014** | **0.010** |
| K02441 | 0.003 | 0.005 | 0.002 | 0.003 | 0.002 | 0.003 | 0.003 | 0.007 | **0.006** | **0.006** | **0.002** | **0.004** |
| K02471 | 0.004 | 0.006 | 0.005 | 0.006 | 0.000 | 0.001 | 0.000 | 0.000 | **0.005** | **0.005** | **0.002** | **0.004** |
| K02472 | 0.002 | 0.004 | 0.002 | 0.003 | 0.001 | 0.003 | 0.003 | 0.004 | **0.001** | **0.002** | **0.007** | **0.007** |
| K02481 | 0.009 | 0.012 | 0.010 | 0.009 | 0.007 | 0.012 | 0.009 | 0.014 | **0.004** | **0.005** | **0.014** | **0.013** |
| K02484 | 0.020 | 0.037 | 0.020 | 0.026 | 0.007 | 0.008 | 0.010 | 0.009 | **0.007** | **0.008** | **0.017** | **0.012** |
| K02494 | 0.004 | 0.007 | 0.004 | 0.009 | 0.001 | 0.001 | 0.000 | 0.001 | **0.003** | **0.004** | **0.000** | **0.001** |
| K02496 | 0.004 | 0.006 | 0.004 | 0.009 | 0.000 | 0.001 | 0.000 | 0.000 | **0.003** | **0.005** | **0.000** | **0.000** |
| K02498 | 0.003 | 0.004 | 0.005 | 0.011 | 0.000 | 0.001 | 0.000 | 0.000 | **0.003** | **0.004** | **0.000** | **0.000** |
| K02501 | 0.077 | 0.019 | 0.064 | 0.017 | 0.073 | 0.015 | 0.071 | 0.013 | **0.063** | **0.016** | **0.075** | **0.016** |
| K02504 | 0.003 | 0.004 | 0.000 | 0.001 | 0.001 | 0.002 | 0.000 | 0.000 | **0.002** | **0.004** | **0.000** | **0.000** |
| K02505 | 0.003 | 0.004 | 0.001 | 0.001 | 0.001 | 0.002 | 0.000 | 0.000 | **0.003** | **0.004** | **0.000** | **0.001** |
| K02507 | 0.003 | 0.005 | 0.001 | 0.001 | 0.001 | 0.001 | 0.000 | 0.000 | **0.003** | **0.004** | **0.000** | **0.001** |
| K02517 | 0.019 | 0.018 | 0.033 | 0.019 | 0.015 | 0.014 | 0.006 | 0.008 | **0.029** | **0.014** | **0.011** | **0.009** |
| K02519 | 0.072 | 0.013 | 0.077 | 0.019 | 0.075 | 0.017 | 0.074 | 0.018 | **0.077** | **0.016** | **0.065** | **0.016** |
| K02521 | 0.003 | 0.004 | 0.000 | 0.001 | 0.000 | 0.001 | 0.000 | 0.000 | **0.003** | **0.004** | **0.000** | **0.000** |
| K02523 | 0.016 | 0.014 | 0.026 | 0.016 | 0.014 | 0.010 | 0.004 | 0.007 | **0.023** | **0.013** | **0.011** | **0.009** |
| K02527 | 0.018 | 0.014 | 0.028 | 0.016 | 0.014 | 0.011 | 0.005 | 0.007 | **0.024** | **0.011** | **0.011** | **0.008** |
| K02529 | 0.384 | 0.094 | 0.336 | 0.118 | 0.332 | 0.057 | 0.392 | 0.054 | **0.334** | **0.042** | **0.375** | **0.043** |
| K02533 | 0.007 | 0.007 | 0.008 | 0.011 | 0.004 | 0.006 | 0.000 | 0.001 | **0.006** | **0.005** | **0.001** | **0.002** |
| K02535 | 0.016 | 0.015 | 0.029 | 0.015 | 0.013 | 0.011 | 0.004 | 0.005 | **0.024** | **0.012** | **0.011** | **0.009** |
| K02536 | 0.020 | 0.019 | 0.031 | 0.015 | 0.014 | 0.014 | 0.004 | 0.008 | **0.024** | **0.011** | **0.011** | **0.009** |
| K02547 | 0.008 | 0.007 | 0.010 | 0.010 | 0.020 | 0.008 | 0.027 | 0.011 | **0.019** | **0.012** | **0.027** | **0.013** |
| K02548 | **0.026** | **0.016** | **0.052** | **0.018** | 0.025 | 0.021 | 0.012 | 0.010 | **0.043** | **0.016** | **0.028** | **0.017** |
| K02549 | 0.008 | 0.008 | 0.015 | 0.011 | 0.007 | 0.011 | 0.000 | 0.000 | **0.018** | **0.010** | **0.002** | **0.004** |
| K02551 | 0.010 | 0.009 | 0.020 | 0.011 | 0.016 | 0.014 | 0.006 | 0.007 | **0.022** | **0.010** | **0.010** | **0.007** |
| K02556 | 0.035 | 0.018 | 0.033 | 0.019 | 0.019 | 0.010 | 0.010 | 0.009 | **0.016** | **0.010** | **0.009** | **0.006** |
| K02557 | 0.049 | 0.025 | 0.062 | 0.017 | 0.044 | 0.013 | 0.026 | 0.012 | **0.045** | **0.015** | **0.031** | **0.012** |
| K02560 | 0.002 | 0.004 | 0.000 | 0.001 | 0.000 | 0.001 | 0.000 | 0.000 | **0.003** | **0.004** | **0.000** | **0.000** |
| K02570 | 0.005 | 0.010 | 0.003 | 0.007 | 0.000 | 0.001 | 0.000 | 0.000 | **0.002** | **0.003** | **0.000** | **0.001** |
| K02619 | **0.006** | **0.007** | **0.019** | **0.010** | 0.011 | 0.011 | 0.004 | 0.006 | **0.018** | **0.011** | **0.008** | **0.007** |
| K02621 | 0.026 | 0.015 | 0.036 | 0.016 | 0.018 | 0.009 | 0.007 | 0.005 | **0.027** | **0.011** | **0.011** | **0.008** |
| K02622 | 0.023 | 0.015 | 0.040 | 0.016 | 0.018 | 0.012 | 0.008 | 0.010 | **0.025** | **0.015** | **0.012** | **0.009** |
| K02639 | 0.001 | 0.005 | 0.001 | 0.002 | 0.000 | 0.001 | 0.000 | 0.000 | **0.001** | **0.002** | **0.000** | **0.000** |
| K02647 | 0.063 | 0.031 | 0.060 | 0.031 | 0.094 | 0.021 | 0.111 | 0.022 | **0.084** | **0.021** | **0.104** | **0.022** |
| K02651 | 0.003 | 0.006 | 0.006 | 0.010 | 0.000 | 0.001 | 0.002 | 0.003 | **0.001** | **0.002** | **0.004** | **0.005** |
| K02655 | 0.001 | 0.004 | 0.006 | 0.013 | 0.000 | 0.001 | 0.000 | 0.001 | **0.004** | **0.010** | **0.000** | **0.000** |
| K02656 | 0.004 | 0.007 | 0.005 | 0.009 | 0.000 | 0.001 | 0.000 | 0.000 | **0.002** | **0.003** | **0.000** | **0.000** |
| K02663 | 0.004 | 0.006 | 0.004 | 0.008 | 0.001 | 0.001 | 0.001 | 0.001 | **0.001** | **0.002** | **0.000** | **0.001** |
| K02664 | 0.002 | 0.006 | 0.004 | 0.007 | 0.000 | 0.001 | 0.000 | 0.000 | **0.001** | **0.002** | **0.000** | **0.000** |
| K02679 | 0.003 | 0.007 | 0.000 | 0.001 | 0.000 | 0.001 | 0.000 | 0.000 | **0.003** | **0.004** | **0.000** | **0.000** |
| K02680 | 0.003 | 0.005 | 0.000 | 0.001 | 0.000 | 0.001 | 0.000 | 0.000 | **0.003** | **0.004** | **0.000** | **0.000** |
| K02682 | 0.002 | 0.005 | 0.000 | 0.001 | 0.000 | 0.001 | 0.000 | 0.000 | **0.003** | **0.004** | **0.000** | **0.001** |
| K02686 | 0.003 | 0.005 | 0.004 | 0.007 | 0.000 | 0.001 | 0.000 | 0.000 | **0.003** | **0.004** | **0.001** | **0.001** |
| K02703 | 0.002 | 0.008 | 0.001 | 0.001 | 0.000 | 0.001 | 0.000 | 0.001 | **0.001** | **0.001** | **0.000** | **0.000** |
| K02770 | 0.054 | 0.025 | 0.049 | 0.020 | 0.054 | 0.021 | 0.060 | 0.019 | **0.053** | **0.013** | **0.064** | **0.017** |
| K02775 | 0.012 | 0.011 | 0.009 | 0.006 | 0.009 | 0.010 | 0.018 | 0.011 | **0.010** | **0.007** | **0.020** | **0.010** |
| K02779 | 0.006 | 0.005 | 0.003 | 0.003 | 0.002 | 0.003 | 0.001 | 0.002 | **0.007** | **0.007** | **0.003** | **0.003** |
| K02784 | 0.003 | 0.005 | 0.000 | 0.001 | 0.000 | 0.001 | 0.000 | 0.001 | **0.002** | **0.003** | **0.000** | **0.001** |
| K02799 | 0.000 | 0.001 | 0.000 | 0.000 | 0.000 | 0.000 | 0.000 | 0.000 | **0.001** | **0.003** | **0.000** | **0.000** |
| K02806 | 0.015 | 0.013 | 0.020 | 0.020 | 0.005 | 0.007 | 0.001 | 0.002 | **0.005** | **0.006** | **0.001** | **0.002** |
| K02824 | 0.055 | 0.019 | 0.054 | 0.018 | 0.054 | 0.019 | 0.051 | 0.017 | **0.058** | **0.016** | **0.045** | **0.013** |
| K02843 | 0.010 | 0.014 | 0.009 | 0.013 | 0.003 | 0.003 | 0.000 | 0.001 | **0.008** | **0.007** | **0.003** | **0.005** |
| K02851 | 0.002 | 0.005 | 0.000 | 0.001 | 0.001 | 0.002 | 0.000 | 0.000 | **0.003** | **0.004** | **0.000** | **0.001** |
| K02852 | 0.002 | 0.006 | 0.001 | 0.002 | 0.000 | 0.001 | 0.003 | 0.006 | **0.001** | **0.002** | **0.006** | **0.007** |
| K02856 | 0.001 | 0.002 | 0.002 | 0.003 | 0.001 | 0.002 | 0.003 | 0.005 | **0.001** | **0.002** | **0.007** | **0.006** |
| K02897 | **0.028** | **0.015** | **0.053** | **0.017** | 0.025 | 0.012 | 0.027 | 0.014 | 0.036 | 0.014 | 0.029 | 0.009 |
| K02913 | 0.073 | 0.023 | 0.077 | 0.015 | 0.067 | 0.014 | 0.060 | 0.022 | **0.074** | **0.017** | **0.056** | **0.014** |
| K02919 | 0.066 | 0.018 | 0.061 | 0.014 | 0.053 | 0.014 | 0.041 | 0.020 | **0.058** | **0.013** | **0.040** | **0.014** |
| K02961 | 0.064 | 0.011 | 0.069 | 0.018 | 0.065 | 0.015 | 0.062 | 0.016 | **0.068** | **0.015** | **0.054** | **0.016** |
| K02968 | 0.071 | 0.016 | 0.074 | 0.019 | 0.072 | 0.014 | 0.065 | 0.020 | **0.072** | **0.017** | **0.060** | **0.014** |
| K03046 | 0.075 | 0.012 | 0.074 | 0.015 | 0.082 | 0.017 | 0.072 | 0.011 | **0.078** | **0.015** | **0.067** | **0.015** |
| K03071 | 0.004 | 0.006 | 0.007 | 0.011 | 0.001 | 0.001 | 0.001 | 0.002 | **0.003** | **0.004** | **0.001** | **0.002** |
| K03072 | 0.030 | 0.019 | 0.030 | 0.018 | 0.018 | 0.012 | 0.013 | 0.011 | **0.017** | **0.010** | **0.011** | **0.008** |
| K03075 | 0.064 | 0.022 | 0.074 | 0.019 | 0.068 | 0.019 | 0.064 | 0.018 | **0.068** | **0.015** | **0.055** | **0.015** |
| K03076 | 0.072 | 0.014 | 0.079 | 0.021 | 0.080 | 0.022 | 0.079 | 0.014 | **0.085** | **0.018** | **0.069** | **0.015** |
| K03077 | 0.003 | 0.003 | 0.003 | 0.003 | 0.006 | 0.004 | 0.011 | 0.010 | **0.004** | **0.004** | **0.013** | **0.009** |
| K03086 | 0.112 | 0.022 | 0.131 | 0.033 | 0.109 | 0.026 | 0.083 | 0.018 | **0.124** | **0.029** | **0.092** | **0.017** |
| K03087 | 0.004 | 0.006 | 0.003 | 0.005 | 0.000 | 0.001 | 0.000 | 0.000 | **0.003** | **0.004** | **0.000** | **0.001** |
| K03088 | 0.572 | 0.175 | 0.512 | 0.142 | 0.660 | 0.078 | 0.639 | 0.095 | **0.596** | **0.067** | **0.669** | **0.097** |
| K03089 | 0.005 | 0.006 | 0.007 | 0.012 | 0.001 | 0.002 | 0.000 | 0.000 | **0.005** | **0.005** | **0.001** | **0.002** |
| K03091 | 0.186 | 0.077 | 0.126 | 0.068 | 0.248 | 0.046 | 0.264 | 0.051 | **0.207** | **0.034** | **0.250** | **0.043** |
| K03092 | 0.037 | 0.013 | 0.053 | 0.020 | 0.031 | 0.017 | 0.018 | 0.008 | **0.038** | **0.015** | **0.025** | **0.012** |
| K03093 | 0.010 | 0.008 | 0.007 | 0.007 | **0.021** | **0.014** | **0.003** | **0.003** | 0.008 | 0.009 | 0.005 | 0.006 |
| K03113 | 0.008 | 0.009 | 0.007 | 0.010 | 0.006 | 0.007 | 0.004 | 0.005 | **0.004** | **0.004** | **0.008** | **0.007** |
| K03149 | 0.027 | 0.013 | 0.025 | 0.012 | 0.033 | 0.013 | 0.044 | 0.014 | **0.031** | **0.014** | **0.042** | **0.014** |
| K03150 | 0.077 | 0.021 | 0.061 | 0.022 | 0.083 | 0.020 | 0.092 | 0.014 | **0.072** | **0.019** | **0.092** | **0.019** |
| K03154 | 0.039 | 0.016 | 0.035 | 0.016 | 0.050 | 0.019 | 0.069 | 0.022 | **0.051** | **0.016** | **0.066** | **0.021** |
| K03181 | 0.002 | 0.004 | 0.002 | 0.004 | 0.000 | 0.001 | 0.000 | 0.000 | **0.002** | **0.004** | **0.000** | **0.001** |
| K03183 | 0.042 | 0.031 | 0.062 | 0.029 | 0.030 | 0.021 | 0.015 | 0.014 | **0.052** | **0.018** | **0.034** | **0.017** |
| K03184 | 0.003 | 0.005 | 0.001 | 0.001 | 0.000 | 0.000 | 0.000 | 0.000 | **0.002** | **0.004** | **0.000** | **0.001** |
| K03185 | 0.004 | 0.006 | 0.005 | 0.009 | 0.000 | 0.001 | 0.000 | 0.001 | **0.003** | **0.004** | **0.000** | **0.001** |
| K03195 | 0.009 | 0.019 | 0.006 | 0.011 | 0.000 | 0.001 | 0.000 | 0.000 | **0.003** | **0.004** | **0.000** | **0.001** |
| K03200 | 0.005 | 0.010 | 0.005 | 0.008 | 0.000 | 0.001 | 0.000 | 0.001 | **0.003** | **0.004** | **0.001** | **0.002** |
| K03204 | 0.012 | 0.022 | 0.006 | 0.009 | 0.000 | 0.000 | 0.000 | 0.000 | **0.003** | **0.004** | **0.001** | **0.002** |
| K03215 | 0.015 | 0.016 | 0.020 | 0.022 | 0.002 | 0.004 | 0.000 | 0.000 | **0.005** | **0.005** | **0.000** | **0.001** |
| K03269 | 0.011 | 0.011 | 0.023 | 0.012 | 0.012 | 0.012 | 0.004 | 0.005 | **0.020** | **0.008** | **0.010** | **0.008** |
| K03270 | 0.020 | 0.017 | 0.028 | 0.011 | 0.015 | 0.012 | 0.012 | 0.009 | **0.026** | **0.014** | **0.014** | **0.011** |
| K03272 | 0.017 | 0.021 | 0.010 | 0.016 | 0.003 | 0.004 | 0.002 | 0.003 | **0.012** | **0.008** | **0.006** | **0.007** |
| K03273 | 0.013 | 0.015 | 0.011 | 0.011 | 0.002 | 0.002 | 0.001 | 0.001 | **0.010** | **0.008** | **0.006** | **0.006** |
| K03281 | 0.009 | 0.007 | 0.020 | 0.013 | 0.013 | 0.010 | 0.004 | 0.005 | **0.020** | **0.012** | **0.009** | **0.007** |
| K03287 | 0.012 | 0.022 | 0.015 | 0.021 | 0.006 | 0.009 | 0.009 | 0.011 | **0.006** | **0.006** | **0.019** | **0.014** |
| K03289 | 0.003 | 0.005 | 0.014 | 0.012 | 0.009 | 0.010 | 0.000 | 0.001 | **0.017** | **0.010** | **0.001** | **0.003** |
| K03292 | 0.076 | 0.028 | 0.075 | 0.033 | 0.108 | 0.029 | 0.133 | 0.035 | **0.096** | **0.030** | **0.126** | **0.032** |
| K03293 | 0.015 | 0.030 | 0.016 | 0.023 | 0.002 | 0.003 | 0.008 | 0.008 | **0.012** | **0.012** | **0.006** | **0.009** |
| K03303 | 0.008 | 0.013 | 0.011 | 0.011 | 0.003 | 0.004 | 0.005 | 0.006 | **0.003** | **0.003** | **0.009** | **0.006** |
| K03305 | 0.017 | 0.017 | 0.020 | 0.011 | 0.014 | 0.014 | 0.007 | 0.008 | **0.023** | **0.011** | **0.013** | **0.011** |
| K03307 | 0.066 | 0.028 | 0.047 | 0.018 | 0.047 | 0.013 | 0.054 | 0.016 | **0.048** | **0.016** | **0.064** | **0.019** |
| K03311 | 0.024 | 0.016 | 0.019 | 0.009 | 0.017 | 0.008 | 0.014 | 0.007 | **0.020** | **0.009** | **0.014** | **0.009** |
| K03314 | 0.003 | 0.005 | 0.001 | 0.003 | 0.000 | 0.000 | 0.000 | 0.000 | **0.003** | **0.004** | **0.000** | **0.001** |
| K03316 | 0.005 | 0.006 | 0.007 | 0.011 | 0.000 | 0.001 | 0.003 | 0.003 | **0.005** | **0.005** | **0.002** | **0.003** |
| K03322 | 0.010 | 0.009 | 0.013 | 0.009 | 0.015 | 0.010 | 0.012 | 0.011 | **0.021** | **0.013** | **0.013** | **0.009** |
| K03327 | 0.019 | 0.009 | 0.037 | 0.017 | 0.025 | 0.021 | 0.009 | 0.012 | **0.040** | **0.018** | **0.021** | **0.016** |
| K03328 | **0.007** | **0.007** | **0.021** | **0.011** | 0.015 | 0.012 | 0.009 | 0.008 | **0.019** | **0.011** | **0.009** | **0.009** |
| K03332 | 0.000 | 0.001 | 0.000 | 0.001 | 0.001 | 0.002 | 0.003 | 0.004 | **0.001** | **0.002** | **0.003** | **0.004** |
| K03335 | 0.005 | 0.006 | 0.005 | 0.005 | 0.003 | 0.004 | 0.003 | 0.004 | **0.002** | **0.002** | **0.005** | **0.005** |
| K03337 | 0.003 | 0.004 | 0.005 | 0.005 | 0.002 | 0.003 | 0.003 | 0.004 | **0.002** | **0.003** | **0.004** | **0.004** |
| K03342 | 0.008 | 0.018 | 0.005 | 0.009 | 0.001 | 0.001 | 0.000 | 0.001 | **0.002** | **0.003** | **0.000** | **0.001** |
| K03385 | 0.008 | 0.013 | 0.016 | 0.014 | 0.008 | 0.013 | 0.001 | 0.002 | **0.016** | **0.010** | **0.004** | **0.005** |
| K03386 | 0.035 | 0.019 | 0.052 | 0.015 | 0.028 | 0.014 | 0.014 | 0.011 | **0.034** | **0.014** | **0.018** | **0.011** |
| K03394 | 0.055 | 0.022 | 0.054 | 0.020 | 0.082 | 0.019 | 0.090 | 0.022 | **0.068** | **0.020** | **0.085** | **0.019** |
| K03408 | 0.068 | 0.040 | 0.079 | 0.048 | 0.056 | 0.027 | 0.034 | 0.017 | **0.038** | **0.015** | **0.029** | **0.013** |
| K03409 | 0.022 | 0.027 | 0.033 | 0.037 | **0.012** | **0.006** | **0.001** | **0.003** | 0.006 | 0.008 | 0.002 | 0.003 |
| K03435 | 0.001 | 0.002 | 0.002 | 0.005 | 0.000 | 0.001 | 0.000 | 0.000 | **0.001** | **0.002** | **0.000** | **0.000** |
| K03442 | 0.057 | 0.023 | 0.034 | 0.014 | 0.056 | 0.013 | 0.063 | 0.018 | **0.053** | **0.012** | **0.063** | **0.015** |
| K03444 | 0.001 | 0.002 | 0.000 | 0.001 | 0.001 | 0.001 | 0.002 | 0.003 | **0.000** | **0.001** | **0.003** | **0.003** |
| K03496 | 0.197 | 0.043 | 0.176 | 0.040 | 0.240 | 0.031 | 0.272 | 0.033 | **0.230** | **0.031** | **0.254** | **0.033** |
| K03517 | 0.040 | 0.015 | 0.043 | 0.017 | 0.044 | 0.014 | 0.060 | 0.012 | **0.046** | **0.012** | **0.057** | **0.015** |
| K03528 | 0.002 | 0.003 | 0.002 | 0.004 | 0.000 | 0.001 | 0.000 | 0.000 | **0.003** | **0.005** | **0.000** | **0.000** |
| K03530 | 0.097 | 0.032 | 0.113 | 0.045 | 0.139 | 0.033 | 0.107 | 0.021 | **0.143** | **0.026** | **0.120** | **0.022** |
| K03534 | 0.015 | 0.015 | 0.011 | 0.007 | 0.011 | 0.010 | 0.014 | 0.013 | **0.009** | **0.007** | **0.019** | **0.013** |
| K03548 | 0.002 | 0.005 | 0.001 | 0.003 | 0.000 | 0.001 | 0.000 | 0.000 | **0.003** | **0.004** | **0.000** | **0.000** |
| K03549 | 0.006 | 0.010 | 0.014 | 0.016 | 0.005 | 0.007 | 0.005 | 0.006 | **0.013** | **0.008** | **0.003** | **0.005** |
| K03554 | 0.003 | 0.004 | 0.004 | 0.008 | 0.001 | 0.001 | 0.000 | 0.001 | **0.002** | **0.004** | **0.000** | **0.000** |
| K03556 | 0.012 | 0.008 | 0.011 | 0.009 | 0.016 | 0.010 | 0.007 | 0.004 | **0.011** | **0.006** | **0.007** | **0.006** |
| K03557 | 0.004 | 0.006 | 0.005 | 0.010 | 0.000 | 0.001 | 0.000 | 0.000 | **0.003** | **0.005** | **0.000** | **0.001** |
| K03560 | 0.003 | 0.005 | 0.005 | 0.008 | 0.000 | 0.001 | 0.000 | 0.000 | **0.003** | **0.004** | **0.000** | **0.001** |
| K03561 | 0.053 | 0.031 | 0.068 | 0.023 | 0.031 | 0.024 | 0.015 | 0.020 | **0.043** | **0.019** | **0.029** | **0.022** |
| K03562 | 0.005 | 0.006 | 0.009 | 0.012 | 0.001 | 0.002 | 0.000 | 0.000 | **0.005** | **0.004** | **0.001** | **0.002** |
| K03573 | 0.004 | 0.005 | 0.000 | 0.001 | 0.001 | 0.002 | 0.001 | 0.002 | **0.003** | **0.003** | **0.000** | **0.001** |
| K03577 | 0.004 | 0.006 | 0.004 | 0.008 | 0.001 | 0.001 | 0.001 | 0.002 | **0.003** | **0.004** | **0.000** | **0.001** |
| K03580 | 0.002 | 0.003 | 0.002 | 0.004 | 0.000 | 0.001 | 0.000 | 0.001 | **0.003** | **0.004** | **0.001** | **0.002** |
| K03582 | 0.005 | 0.006 | 0.005 | 0.007 | 0.001 | 0.002 | 0.000 | 0.000 | **0.003** | **0.004** | **0.000** | **0.001** |
| K03583 | 0.004 | 0.006 | 0.003 | 0.006 | 0.001 | 0.002 | 0.000 | 0.001 | **0.003** | **0.004** | **0.000** | **0.001** |
| K03585 | 0.023 | 0.019 | 0.042 | 0.022 | 0.024 | 0.016 | 0.011 | 0.013 | **0.040** | **0.017** | **0.025** | **0.018** |
| K03586 | 0.003 | 0.007 | 0.004 | 0.009 | 0.000 | 0.001 | 0.000 | 0.000 | **0.003** | **0.004** | **0.000** | **0.000** |
| K03587 | 0.027 | 0.021 | 0.042 | 0.016 | 0.015 | 0.011 | 0.007 | 0.007 | **0.027** | **0.011** | **0.013** | **0.010** |
| K03590 | 0.026 | 0.018 | 0.040 | 0.016 | 0.022 | 0.016 | 0.006 | 0.008 | **0.026** | **0.012** | **0.013** | **0.008** |
| K03591 | 0.002 | 0.004 | 0.000 | 0.001 | 0.000 | 0.001 | 0.000 | 0.000 | **0.002** | **0.004** | **0.000** | **0.000** |
| K03593 | 0.018 | 0.016 | 0.025 | 0.015 | 0.013 | 0.012 | 0.009 | 0.009 | **0.023** | **0.010** | **0.011** | **0.009** |
| K03597 | 0.003 | 0.005 | 0.003 | 0.007 | 0.000 | 0.001 | 0.000 | 0.000 | **0.002** | **0.003** | **0.000** | **0.001** |
| K03598 | 0.004 | 0.006 | 0.003 | 0.007 | 0.001 | 0.001 | 0.001 | 0.003 | **0.003** | **0.004** | **0.000** | **0.001** |
| K03599 | 0.004 | 0.008 | 0.005 | 0.009 | 0.000 | 0.001 | 0.000 | 0.000 | **0.003** | **0.004** | **0.000** | **0.001** |
| K03600 | 0.003 | 0.005 | 0.006 | 0.010 | 0.001 | 0.002 | 0.000 | 0.000 | **0.003** | **0.005** | **0.001** | **0.002** |
| K03604 | **0.010** | **0.009** | **0.002** | **0.002** | 0.005 | 0.005 | 0.010 | 0.008 | 0.005 | 0.005 | 0.006 | 0.006 |
| K03606 | 0.003 | 0.004 | 0.005 | 0.006 | 0.000 | 0.001 | 0.006 | 0.009 | **0.001** | **0.003** | **0.010** | **0.010** |
| K03607 | 0.002 | 0.003 | 0.000 | 0.001 | 0.001 | 0.001 | 0.000 | 0.000 | **0.002** | **0.003** | **0.000** | **0.001** |
| K03611 | 0.007 | 0.011 | 0.006 | 0.010 | 0.001 | 0.002 | 0.000 | 0.001 | **0.004** | **0.005** | **0.001** | **0.002** |
| K03613 | 0.065 | 0.019 | 0.059 | 0.026 | 0.073 | 0.015 | 0.072 | 0.011 | **0.059** | **0.016** | **0.072** | **0.016** |
| K03615 | 0.067 | 0.018 | 0.060 | 0.026 | 0.081 | 0.016 | 0.083 | 0.013 | **0.068** | **0.018** | **0.082** | **0.017** |
| K03617 | 0.069 | 0.020 | 0.054 | 0.022 | 0.066 | 0.016 | 0.069 | 0.016 | **0.060** | **0.013** | **0.070** | **0.016** |
| K03632 | 0.003 | 0.006 | 0.000 | 0.001 | 0.000 | 0.001 | 0.000 | 0.000 | **0.002** | **0.003** | **0.000** | **0.000** |
| K03633 | 0.002 | 0.005 | 0.000 | 0.001 | 0.000 | 0.001 | 0.000 | 0.000 | **0.003** | **0.004** | **0.000** | **0.001** |
| K03634 | 0.009 | 0.017 | 0.008 | 0.011 | 0.001 | 0.002 | 0.000 | 0.001 | **0.004** | **0.005** | **0.001** | **0.002** |
| K03640 | 0.016 | 0.014 | 0.028 | 0.016 | 0.008 | 0.009 | 0.001 | 0.001 | **0.021** | **0.012** | **0.004** | **0.005** |
| K03641 | 0.013 | 0.014 | 0.010 | 0.012 | 0.000 | 0.001 | 0.000 | 0.000 | **0.006** | **0.006** | **0.001** | **0.002** |
| K03642 | 0.019 | 0.019 | 0.039 | 0.018 | 0.008 | 0.008 | 0.000 | 0.001 | **0.019** | **0.011** | **0.002** | **0.004** |
| K03643 | 0.004 | 0.007 | 0.005 | 0.009 | 0.000 | 0.001 | 0.000 | 0.000 | **0.002** | **0.003** | **0.000** | **0.001** |
| K03645 | 0.002 | 0.003 | 0.000 | 0.001 | 0.000 | 0.001 | 0.000 | 0.000 | **0.002** | **0.004** | **0.000** | **0.000** |
| K03651 | 0.003 | 0.004 | 0.002 | 0.004 | 0.000 | 0.001 | 0.000 | 0.000 | **0.002** | **0.004** | **0.000** | **0.001** |
| K03656 | 0.002 | 0.003 | 0.005 | 0.012 | 0.001 | 0.001 | 0.000 | 0.001 | **0.003** | **0.004** | **0.000** | **0.001** |
| K03667 | 0.019 | 0.020 | 0.024 | 0.019 | 0.003 | 0.006 | 0.000 | 0.001 | **0.008** | **0.007** | **0.001** | **0.002** |
| K03673 | 0.012 | 0.028 | 0.007 | 0.011 | 0.001 | 0.002 | 0.001 | 0.002 | **0.004** | **0.004** | **0.001** | **0.002** |
| K03674 | 0.002 | 0.003 | 0.000 | 0.001 | 0.000 | 0.001 | 0.000 | 0.000 | **0.003** | **0.004** | **0.000** | **0.000** |
| K03683 | 0.002 | 0.005 | 0.002 | 0.005 | 0.001 | 0.002 | 0.000 | 0.000 | **0.002** | **0.004** | **0.000** | **0.001** |
| K03690 | 0.005 | 0.010 | 0.004 | 0.009 | 0.001 | 0.003 | 0.000 | 0.000 | **0.003** | **0.004** | **0.000** | **0.000** |
| K03703 | 0.076 | 0.020 | 0.073 | 0.017 | 0.077 | 0.018 | 0.071 | 0.015 | **0.081** | **0.015** | **0.065** | **0.015** |
| K03709 | 0.016 | 0.015 | 0.019 | 0.013 | 0.002 | 0.003 | 0.001 | 0.001 | **0.005** | **0.005** | **0.002** | **0.003** |
| K03711 | 0.082 | 0.024 | 0.091 | 0.030 | 0.113 | 0.023 | 0.102 | 0.023 | **0.118** | **0.016** | **0.103** | **0.018** |
| K03712 | 0.060 | 0.029 | 0.046 | 0.020 | 0.064 | 0.019 | 0.072 | 0.017 | **0.061** | **0.016** | **0.073** | **0.017** |
| K03718 | 0.010 | 0.011 | 0.030 | 0.018 | 0.020 | 0.021 | 0.009 | 0.012 | **0.034** | **0.016** | **0.015** | **0.012** |
| K03720 | 0.009 | 0.014 | 0.014 | 0.020 | 0.002 | 0.005 | 0.000 | 0.000 | **0.001** | **0.003** | **0.000** | **0.000** |
| K03732 | 0.012 | 0.012 | 0.014 | 0.016 | 0.002 | 0.004 | 0.000 | 0.001 | **0.003** | **0.004** | **0.000** | **0.001** |
| K03733 | 0.026 | 0.014 | 0.045 | 0.017 | 0.018 | 0.012 | 0.012 | 0.007 | **0.027** | **0.010** | **0.013** | **0.009** |
| K03742 | 0.043 | 0.017 | 0.038 | 0.020 | 0.046 | 0.012 | 0.030 | 0.011 | **0.044** | **0.015** | **0.032** | **0.014** |
| K03744 | 0.033 | 0.012 | 0.050 | 0.015 | 0.029 | 0.016 | 0.023 | 0.012 | **0.041** | **0.015** | **0.028** | **0.011** |
| K03745 | 0.005 | 0.009 | 0.004 | 0.008 | 0.001 | 0.002 | 0.000 | 0.000 | **0.002** | **0.003** | **0.000** | **0.001** |
| K03746 | 0.006 | 0.012 | 0.013 | 0.021 | 0.000 | 0.001 | 0.000 | 0.001 | **0.004** | **0.005** | **0.001** | **0.002** |
| K03749 | 0.005 | 0.010 | 0.005 | 0.011 | 0.001 | 0.001 | 0.000 | 0.000 | **0.003** | **0.004** | **0.000** | **0.001** |
| K03764 | 0.003 | 0.004 | 0.000 | 0.001 | 0.001 | 0.002 | 0.000 | 0.000 | **0.003** | **0.003** | **0.000** | **0.000** |
| K03770 | **0.013** | **0.021** | **0.039** | **0.020** | 0.022 | 0.022 | 0.005 | 0.006 | **0.036** | **0.016** | **0.010** | **0.009** |
| K03771 | 0.013 | 0.010 | 0.025 | 0.012 | 0.011 | 0.011 | 0.006 | 0.009 | **0.021** | **0.011** | **0.013** | **0.010** |
| K03773 | **0.007** | **0.007** | **0.028** | **0.016** | 0.019 | 0.018 | 0.006 | 0.007 | **0.032** | **0.016** | **0.011** | **0.010** |
| K03775 | 0.015 | 0.016 | 0.026 | 0.016 | 0.015 | 0.012 | 0.005 | 0.007 | **0.023** | **0.011** | **0.008** | **0.007** |
| K03778 | 0.032 | 0.017 | 0.035 | 0.016 | 0.033 | 0.015 | 0.040 | 0.015 | **0.028** | **0.011** | **0.039** | **0.013** |
| K03782 | 0.007 | 0.007 | 0.010 | 0.018 | 0.001 | 0.001 | 0.000 | 0.001 | **0.003** | **0.004** | **0.001** | **0.002** |
| K03786 | 0.055 | 0.020 | 0.045 | 0.019 | 0.046 | 0.015 | 0.036 | 0.015 | **0.048** | **0.015** | **0.033** | **0.016** |
| K03787 | 0.028 | 0.021 | 0.045 | 0.018 | 0.019 | 0.014 | 0.006 | 0.007 | **0.026** | **0.010** | **0.013** | **0.009** |
| K03797 | 0.082 | 0.023 | 0.111 | 0.033 | 0.095 | 0.024 | 0.076 | 0.023 | **0.110** | **0.024** | **0.083** | **0.022** |
| K03804 | 0.002 | 0.003 | 0.001 | 0.002 | 0.000 | 0.001 | 0.000 | 0.000 | **0.003** | **0.003** | **0.000** | **0.000** |
| K03806 | 0.003 | 0.008 | 0.004 | 0.008 | 0.000 | 0.002 | 0.000 | 0.000 | **0.003** | **0.004** | **0.000** | **0.001** |
| K03814 | **0.005** | **0.008** | **0.021** | **0.012** | 0.008 | 0.011 | 0.003 | 0.003 | **0.017** | **0.012** | **0.007** | **0.006** |
| K03818 | 0.000 | 0.001 | 0.001 | 0.002 | 0.000 | 0.000 | 0.003 | 0.004 | **0.000** | **0.001** | **0.002** | **0.003** |
| K03820 | 0.027 | 0.029 | 0.041 | 0.037 | 0.008 | 0.008 | 0.001 | 0.001 | **0.008** | **0.007** | **0.003** | **0.003** |
| K03826 | 0.024 | 0.017 | 0.019 | 0.011 | 0.028 | 0.013 | 0.030 | 0.017 | **0.024** | **0.011** | **0.034** | **0.013** |
| K03827 | 0.005 | 0.004 | 0.007 | 0.005 | 0.009 | 0.009 | 0.007 | 0.008 | **0.005** | **0.006** | **0.009** | **0.007** |
| K03832 | 0.039 | 0.030 | 0.064 | 0.026 | 0.021 | 0.021 | 0.010 | 0.013 | **0.038** | **0.018** | **0.020** | **0.015** |
| K03834 | 0.003 | 0.005 | 0.000 | 0.001 | 0.001 | 0.002 | 0.000 | 0.000 | **0.002** | **0.003** | **0.000** | **0.000** |
| K03835 | 0.004 | 0.006 | 0.002 | 0.006 | 0.001 | 0.001 | 0.000 | 0.000 | **0.003** | **0.004** | **0.000** | **0.001** |
| K03837 | 0.004 | 0.008 | 0.003 | 0.004 | 0.000 | 0.001 | 0.000 | 0.000 | **0.003** | **0.004** | **0.000** | **0.002** |
| K03839 | 0.016 | 0.015 | 0.018 | 0.013 | 0.013 | 0.011 | 0.006 | 0.007 | **0.021** | **0.010** | **0.011** | **0.009** |
| K03885 | 0.023 | 0.016 | 0.034 | 0.014 | 0.012 | 0.011 | 0.003 | 0.004 | **0.023** | **0.011** | **0.007** | **0.007** |
| K03931 | 0.001 | 0.002 | 0.001 | 0.003 | 0.000 | 0.000 | 0.004 | 0.007 | **0.000** | **0.001** | **0.005** | **0.005** |
| K03980 | 0.036 | 0.024 | 0.031 | 0.019 | 0.012 | 0.009 | 0.005 | 0.003 | **0.013** | **0.008** | **0.007** | **0.006** |
| K04042 | 0.051 | 0.017 | 0.050 | 0.024 | 0.044 | 0.015 | 0.029 | 0.012 | **0.045** | **0.017** | **0.027** | **0.014** |
| K04047 | 0.017 | 0.014 | 0.022 | 0.015 | 0.008 | 0.011 | 0.003 | 0.005 | **0.021** | **0.010** | **0.008** | **0.006** |
| K04069 | 0.155 | 0.051 | 0.142 | 0.052 | 0.168 | 0.032 | 0.158 | 0.034 | **0.145** | **0.022** | **0.167** | **0.027** |
| K04072 | 0.084 | 0.028 | 0.063 | 0.027 | 0.075 | 0.023 | 0.090 | 0.035 | **0.069** | **0.022** | **0.083** | **0.023** |
| K04075 | 0.076 | 0.020 | 0.075 | 0.017 | 0.078 | 0.019 | 0.064 | 0.016 | **0.077** | **0.018** | **0.064** | **0.015** |
| K04084 | 0.006 | 0.012 | 0.005 | 0.008 | 0.000 | 0.001 | 0.000 | 0.000 | **0.003** | **0.005** | **0.000** | **0.001** |
| K04092 | 0.002 | 0.003 | 0.002 | 0.004 | **0.000** | **0.000** | **0.004** | **0.004** | 0.001 | 0.002 | 0.001 | 0.002 |
| K04095 | 0.016 | 0.015 | 0.021 | 0.014 | 0.014 | 0.008 | 0.010 | 0.008 | **0.025** | **0.012** | **0.008** | **0.006** |
| K04477 | 0.045 | 0.024 | 0.032 | 0.020 | 0.050 | 0.016 | 0.057 | 0.020 | **0.045** | **0.016** | **0.063** | **0.019** |
| K04486 | 0.079 | 0.022 | 0.067 | 0.027 | 0.082 | 0.019 | 0.094 | 0.022 | **0.075** | **0.015** | **0.090** | **0.019** |
| K04516 | 0.007 | 0.007 | 0.016 | 0.011 | 0.013 | 0.013 | 0.003 | 0.006 | **0.017** | **0.010** | **0.009** | **0.009** |
| K04568 | 0.013 | 0.015 | 0.016 | 0.017 | 0.003 | 0.007 | 0.000 | 0.000 | **0.004** | **0.004** | **0.000** | **0.000** |
| K04653 | 0.010 | 0.008 | 0.011 | 0.009 | 0.010 | 0.009 | 0.004 | 0.003 | **0.009** | **0.005** | **0.006** | **0.005** |
| K04691 | 0.002 | 0.004 | 0.002 | 0.005 | 0.001 | 0.001 | 0.000 | 0.000 | **0.003** | **0.004** | **0.000** | **0.000** |
| K04720 | 0.043 | 0.025 | 0.039 | 0.026 | 0.071 | 0.019 | 0.095 | 0.024 | **0.067** | **0.020** | **0.089** | **0.026** |
| K04744 | 0.008 | 0.011 | 0.007 | 0.012 | 0.001 | 0.002 | 0.000 | 0.001 | **0.005** | **0.005** | **0.001** | **0.002** |
| K04754 | 0.009 | 0.014 | 0.009 | 0.014 | 0.000 | 0.001 | 0.000 | 0.000 | **0.004** | **0.005** | **0.001** | **0.002** |
| K04758 | 0.163 | 0.051 | 0.119 | 0.041 | 0.166 | 0.030 | 0.179 | 0.034 | **0.151** | **0.026** | **0.173** | **0.029** |
| K04760 | 0.004 | 0.005 | 0.005 | 0.011 | 0.001 | 0.002 | 0.000 | 0.000 | **0.003** | **0.004** | **0.001** | **0.002** |
| K04761 | 0.009 | 0.007 | 0.021 | 0.013 | 0.010 | 0.013 | 0.003 | 0.004 | **0.019** | **0.009** | **0.008** | **0.007** |
| K04762 | 0.013 | 0.010 | 0.024 | 0.013 | 0.012 | 0.011 | 0.004 | 0.007 | **0.021** | **0.011** | **0.010** | **0.007** |
| K04764 | 0.008 | 0.008 | 0.011 | 0.012 | 0.002 | 0.004 | 0.000 | 0.001 | **0.006** | **0.006** | **0.003** | **0.004** |
| K04769 | 0.041 | 0.018 | 0.026 | 0.016 | 0.046 | 0.017 | 0.052 | 0.025 | **0.038** | **0.013** | **0.048** | **0.015** |
| K04770 | 0.002 | 0.004 | 0.000 | 0.001 | 0.001 | 0.001 | 0.000 | 0.000 | **0.003** | **0.005** | **0.000** | **0.000** |
| K04773 | 0.018 | 0.017 | 0.029 | 0.016 | 0.015 | 0.013 | 0.006 | 0.005 | **0.025** | **0.013** | **0.013** | **0.009** |
| K04774 | 0.002 | 0.004 | 0.002 | 0.004 | 0.000 | 0.001 | 0.000 | 0.000 | **0.003** | **0.003** | **0.000** | **0.000** |
| K05340 | 0.003 | 0.005 | 0.001 | 0.002 | 0.001 | 0.001 | 0.005 | 0.005 | **0.001** | **0.003** | **0.007** | **0.008** |
| K05343 | 0.011 | 0.012 | 0.004 | 0.005 | 0.003 | 0.004 | 0.011 | 0.011 | **0.005** | **0.007** | **0.011** | **0.010** |
| K05349 | 0.132 | 0.058 | 0.139 | 0.054 | 0.197 | 0.033 | 0.259 | 0.051 | **0.183** | **0.041** | **0.237** | **0.049** |
| K05364 | 0.030 | 0.019 | 0.021 | 0.014 | 0.035 | 0.011 | 0.050 | 0.011 | **0.033** | **0.012** | **0.044** | **0.014** |
| K05365 | 0.006 | 0.009 | 0.003 | 0.008 | 0.001 | 0.003 | 0.000 | 0.001 | **0.006** | **0.007** | **0.000** | **0.001** |
| K05366 | 0.148 | 0.031 | 0.181 | 0.035 | 0.170 | 0.032 | 0.146 | 0.018 | **0.188** | **0.032** | **0.155** | **0.021** |
| K05368 | 0.002 | 0.005 | 0.001 | 0.002 | 0.000 | 0.001 | 0.000 | 0.001 | **0.003** | **0.004** | **0.000** | **0.000** |
| K05516 | 0.010 | 0.015 | 0.022 | 0.014 | 0.009 | 0.010 | 0.002 | 0.005 | **0.020** | **0.012** | **0.008** | **0.007** |
| K05521 | 0.037 | 0.018 | 0.044 | 0.017 | 0.020 | 0.013 | 0.021 | 0.017 | **0.028** | **0.011** | **0.020** | **0.012** |
| K05527 | 0.005 | 0.006 | 0.005 | 0.008 | 0.000 | 0.001 | 0.000 | 0.000 | **0.003** | **0.003** | **0.000** | **0.001** |
| K05539 | 0.003 | 0.004 | 0.005 | 0.010 | 0.000 | 0.001 | 0.000 | 0.000 | **0.003** | **0.004** | **0.000** | **0.001** |
| K05541 | 0.003 | 0.005 | 0.004 | 0.009 | 0.000 | 0.001 | 0.000 | 0.000 | **0.003** | **0.004** | **0.000** | **0.001** |
| K05575 | 0.001 | 0.004 | 0.000 | 0.001 | 0.000 | 0.001 | 0.000 | 0.000 | **0.001** | **0.002** | **0.000** | **0.000** |
| K05589 | 0.003 | 0.005 | 0.006 | 0.010 | 0.000 | 0.001 | 0.000 | 0.001 | **0.003** | **0.004** | **0.001** | **0.002** |
| K05590 | 0.003 | 0.004 | 0.000 | 0.001 | 0.000 | 0.001 | 0.000 | 0.001 | **0.003** | **0.005** | **0.000** | **0.000** |
| K05592 | 0.039 | 0.015 | 0.040 | 0.018 | 0.042 | 0.015 | 0.031 | 0.015 | **0.047** | **0.014** | **0.035** | **0.013** |
| K05595 | 0.023 | 0.018 | 0.026 | 0.021 | 0.005 | 0.005 | 0.010 | 0.017 | **0.008** | **0.007** | **0.017** | **0.013** |
| K05685 | 0.005 | 0.007 | 0.007 | 0.012 | 0.001 | 0.002 | 0.000 | 0.000 | **0.004** | **0.005** | **0.000** | **0.001** |
| K05785 | 0.001 | 0.002 | 0.001 | 0.002 | 0.000 | 0.001 | 0.003 | 0.005 | **0.001** | **0.002** | **0.008** | **0.009** |
| K05786 | 0.005 | 0.008 | 0.007 | 0.013 | 0.000 | 0.001 | 0.000 | 0.001 | **0.005** | **0.005** | **0.003** | **0.004** |
| K05787 | 0.003 | 0.004 | 0.001 | 0.003 | 0.000 | 0.001 | 0.000 | 0.000 | **0.002** | **0.004** | **0.000** | **0.000** |
| K05788 | 0.013 | 0.013 | 0.022 | 0.019 | 0.001 | 0.001 | 0.000 | 0.000 | **0.004** | **0.005** | **0.000** | **0.001** |
| K05801 | 0.009 | 0.010 | 0.014 | 0.012 | 0.006 | 0.010 | 0.004 | 0.007 | **0.015** | **0.010** | **0.005** | **0.006** |
| K05802 | 0.004 | 0.007 | 0.002 | 0.003 | 0.000 | 0.001 | 0.000 | 0.000 | **0.003** | **0.004** | **0.000** | **0.000** |
| K05803 | 0.002 | 0.004 | 0.000 | 0.001 | 0.001 | 0.002 | 0.000 | 0.000 | **0.002** | **0.004** | **0.000** | **0.000** |
| K05807 | 0.014 | 0.011 | 0.026 | 0.017 | 0.013 | 0.013 | 0.005 | 0.007 | **0.023** | **0.011** | **0.009** | **0.009** |
| K05809 | 0.002 | 0.004 | 0.000 | 0.001 | 0.000 | 0.001 | 0.000 | 0.000 | **0.002** | **0.003** | **0.000** | **0.000** |
| K05846 | 0.027 | 0.018 | 0.031 | 0.016 | 0.016 | 0.008 | 0.010 | 0.007 | **0.015** | **0.007** | **0.011** | **0.008** |
| K05851 | 0.002 | 0.004 | 0.001 | 0.003 | 0.000 | 0.001 | 0.000 | 0.000 | **0.003** | **0.004** | **0.000** | **0.001** |
| K05878 | 0.035 | 0.017 | 0.030 | 0.017 | 0.017 | 0.011 | 0.021 | 0.010 | **0.019** | **0.009** | **0.029** | **0.014** |
| K05884 | **0.006** | **0.008** | **0.000** | **0.001** | 0.001 | 0.002 | 0.006 | 0.006 | 0.001 | 0.003 | 0.004 | 0.006 |
| K05919 | 0.043 | 0.023 | 0.029 | 0.016 | 0.058 | 0.018 | 0.044 | 0.014 | **0.047** | **0.017** | **0.036** | **0.012** |
| K05921 | 0.002 | 0.003 | 0.006 | 0.014 | 0.000 | 0.001 | 0.000 | 0.001 | **0.002** | **0.004** | **0.000** | **0.001** |
| K05939 | 0.013 | 0.017 | 0.005 | 0.010 | 0.001 | 0.002 | 0.000 | 0.001 | **0.004** | **0.004** | **0.002** | **0.004** |
| K05945 | 0.001 | 0.002 | 0.000 | 0.000 | 0.000 | 0.000 | 0.001 | 0.003 | **0.000** | **0.001** | **0.002** | **0.003** |
| K05964 | 0.000 | 0.001 | 0.002 | 0.003 | 0.001 | 0.002 | 0.000 | 0.000 | **0.002** | **0.004** | **0.000** | **0.001** |
| K05989 | 0.038 | 0.021 | 0.022 | 0.013 | 0.040 | 0.011 | 0.058 | 0.019 | **0.037** | **0.015** | **0.053** | **0.016** |
| K06001 | 0.033 | 0.017 | 0.031 | 0.018 | 0.018 | 0.011 | 0.017 | 0.013 | **0.014** | **0.008** | **0.023** | **0.012** |
| K06012 | 0.047 | 0.020 | 0.030 | 0.019 | 0.057 | 0.016 | 0.057 | 0.020 | **0.042** | **0.012** | **0.055** | **0.018** |
| K06016 | 0.010 | 0.011 | 0.017 | 0.019 | 0.005 | 0.004 | 0.002 | 0.003 | **0.006** | **0.005** | **0.013** | **0.010** |
| K06023 | 0.123 | 0.036 | 0.105 | 0.033 | 0.129 | 0.025 | 0.134 | 0.026 | **0.111** | **0.024** | **0.130** | **0.025** |
| K06041 | 0.028 | 0.023 | 0.041 | 0.013 | 0.015 | 0.012 | 0.007 | 0.008 | **0.027** | **0.013** | **0.012** | **0.009** |
| K06075 | 0.002 | 0.005 | 0.008 | 0.011 | 0.001 | 0.002 | 0.000 | 0.001 | **0.001** | **0.003** | **0.000** | **0.001** |
| K06077 | 0.005 | 0.009 | 0.002 | 0.005 | 0.000 | 0.001 | 0.000 | 0.000 | **0.003** | **0.003** | **0.000** | **0.001** |
| K06079 | 0.001 | 0.002 | 0.002 | 0.003 | 0.000 | 0.001 | 0.002 | 0.003 | **0.000** | **0.001** | **0.004** | **0.005** |
| K06113 | 0.001 | 0.002 | 0.004 | 0.004 | 0.001 | 0.001 | 0.005 | 0.010 | **0.001** | **0.003** | **0.008** | **0.008** |
| K06133 | 0.036 | 0.018 | 0.026 | 0.012 | 0.039 | 0.013 | 0.050 | 0.013 | **0.038** | **0.015** | **0.049** | **0.016** |
| K06138 | 0.002 | 0.003 | 0.001 | 0.004 | 0.000 | 0.001 | 0.002 | 0.003 | **0.000** | **0.001** | **0.003** | **0.004** |
| K06142 | **0.034** | **0.023** | **0.085** | **0.032** | 0.054 | 0.049 | 0.014 | 0.013 | **0.085** | **0.036** | **0.031** | **0.022** |
| K06145 | 0.004 | 0.006 | 0.007 | 0.014 | 0.000 | 0.001 | 0.000 | 0.000 | **0.002** | **0.004** | **0.000** | **0.000** |
| K06147 | 0.595 | 0.153 | 0.493 | 0.137 | 0.710 | 0.087 | 0.722 | 0.071 | **0.634** | **0.064** | **0.679** | **0.079** |
| K06149 | 0.003 | 0.005 | 0.002 | 0.004 | 0.001 | 0.002 | 0.000 | 0.000 | **0.003** | **0.004** | **0.000** | **0.000** |
| K06167 | 0.021 | 0.017 | 0.036 | 0.018 | 0.017 | 0.014 | 0.005 | 0.009 | **0.022** | **0.009** | **0.011** | **0.009** |
| K06177 | 0.017 | 0.013 | 0.038 | 0.020 | 0.024 | 0.021 | 0.007 | 0.009 | **0.038** | **0.016** | **0.013** | **0.012** |
| K06178 | 0.061 | 0.017 | 0.056 | 0.017 | 0.044 | 0.016 | 0.027 | 0.010 | **0.049** | **0.016** | **0.035** | **0.017** |
| K06180 | 0.171 | 0.036 | 0.187 | 0.054 | 0.212 | 0.026 | 0.213 | 0.024 | **0.217** | **0.030** | **0.194** | **0.026** |
| K06186 | 0.003 | 0.005 | 0.004 | 0.009 | 0.000 | 0.001 | 0.000 | 0.001 | **0.003** | **0.004** | **0.000** | **0.001** |
| K06189 | 0.004 | 0.007 | 0.005 | 0.009 | 0.001 | 0.002 | 0.000 | 0.000 | **0.003** | **0.005** | **0.001** | **0.002** |
| K06190 | 0.005 | 0.008 | 0.005 | 0.009 | 0.001 | 0.001 | 0.000 | 0.001 | **0.002** | **0.003** | **0.000** | **0.001** |
| K06203 | 0.003 | 0.005 | 0.002 | 0.003 | 0.001 | 0.002 | 0.000 | 0.000 | **0.003** | **0.004** | **0.000** | **0.001** |
| K06204 | 0.010 | 0.012 | 0.011 | 0.017 | 0.000 | 0.001 | 0.000 | 0.001 | **0.005** | **0.005** | **0.001** | **0.002** |
| K06205 | 0.003 | 0.004 | 0.001 | 0.004 | 0.000 | 0.001 | 0.000 | 0.000 | **0.003** | **0.004** | **0.000** | **0.001** |
| K06213 | 0.060 | 0.016 | 0.057 | 0.018 | 0.063 | 0.017 | 0.073 | 0.015 | **0.053** | **0.012** | **0.066** | **0.015** |
| K06218 | 0.005 | 0.006 | 0.004 | 0.004 | 0.004 | 0.007 | 0.001 | 0.002 | **0.004** | **0.005** | **0.001** | **0.002** |
| K06297 | 0.003 | 0.004 | 0.003 | 0.003 | **0.009** | **0.008** | **0.001** | **0.001** | 0.003 | 0.004 | 0.002 | 0.003 |
| K06298 | 0.017 | 0.013 | 0.017 | 0.013 | 0.026 | 0.011 | 0.027 | 0.017 | **0.025** | **0.012** | **0.033** | **0.013** |
| K06310 | 0.009 | 0.008 | 0.010 | 0.009 | 0.022 | 0.011 | 0.034 | 0.018 | **0.022** | **0.010** | **0.030** | **0.012** |
| K06333 | 0.035 | 0.020 | 0.024 | 0.014 | 0.040 | 0.014 | 0.043 | 0.016 | **0.033** | **0.012** | **0.044** | **0.015** |
| K06392 | 0.052 | 0.027 | 0.027 | 0.017 | 0.062 | 0.015 | 0.077 | 0.019 | **0.049** | **0.015** | **0.061** | **0.016** |
| K06393 | 0.045 | 0.023 | 0.025 | 0.015 | 0.052 | 0.014 | 0.061 | 0.014 | **0.047** | **0.012** | **0.058** | **0.016** |
| K06394 | 0.028 | 0.016 | 0.019 | 0.013 | 0.034 | 0.014 | 0.033 | 0.016 | **0.023** | **0.010** | **0.033** | **0.011** |
| K06400 | 0.033 | 0.018 | 0.027 | 0.016 | 0.051 | 0.013 | 0.054 | 0.017 | **0.041** | **0.014** | **0.060** | **0.015** |
| K06404 | 0.016 | 0.013 | 0.013 | 0.011 | 0.026 | 0.013 | 0.043 | 0.011 | **0.028** | **0.012** | **0.039** | **0.016** |
| K06406 | 0.052 | 0.023 | 0.031 | 0.020 | 0.060 | 0.016 | 0.069 | 0.014 | **0.053** | **0.014** | **0.063** | **0.016** |
| K06407 | 0.051 | 0.024 | 0.032 | 0.019 | 0.058 | 0.017 | 0.063 | 0.016 | **0.051** | **0.015** | **0.063** | **0.015** |
| K06409 | 0.065 | 0.033 | 0.043 | 0.025 | 0.089 | 0.020 | 0.111 | 0.019 | **0.083** | **0.020** | **0.104** | **0.023** |
| K06412 | 0.056 | 0.020 | 0.041 | 0.018 | 0.067 | 0.022 | 0.073 | 0.017 | **0.054** | **0.017** | **0.069** | **0.018** |
| K06605 | 0.002 | 0.003 | 0.001 | 0.003 | 0.002 | 0.003 | 0.002 | 0.002 | **0.001** | **0.002** | **0.003** | **0.004** |
| K06606 | 0.006 | 0.007 | 0.002 | 0.004 | 0.003 | 0.004 | 0.007 | 0.010 | **0.003** | **0.005** | **0.006** | **0.006** |
| K06859 | 0.001 | 0.002 | 0.003 | 0.004 | 0.003 | 0.004 | 0.005 | 0.004 | **0.003** | **0.003** | **0.007** | **0.007** |
| K06861 | 0.029 | 0.023 | 0.042 | 0.014 | 0.017 | 0.013 | 0.005 | 0.006 | **0.027** | **0.012** | **0.012** | **0.010** |
| K06879 | 0.002 | 0.003 | 0.004 | 0.008 | 0.000 | 0.001 | 0.001 | 0.001 | **0.003** | **0.005** | **0.000** | **0.001** |
| K06887 | 0.002 | 0.004 | 0.000 | 0.001 | 0.000 | 0.001 | 0.000 | 0.000 | **0.003** | **0.006** | **0.000** | **0.000** |
| K06890 | 0.032 | 0.019 | 0.044 | 0.017 | 0.026 | 0.013 | 0.017 | 0.009 | **0.036** | **0.016** | **0.012** | **0.008** |
| K06894 | 0.012 | 0.015 | 0.021 | 0.020 | 0.001 | 0.001 | 0.002 | 0.004 | **0.001** | **0.002** | **0.004** | **0.006** |
| K06895 | 0.012 | 0.012 | 0.022 | 0.014 | 0.016 | 0.014 | 0.009 | 0.005 | **0.020** | **0.012** | **0.011** | **0.009** |
| K06899 | 0.003 | 0.005 | 0.001 | 0.004 | 0.000 | 0.001 | 0.000 | 0.000 | **0.002** | **0.003** | **0.000** | **0.000** |
| K06902 | 0.021 | 0.014 | 0.012 | 0.008 | 0.018 | 0.012 | 0.024 | 0.014 | **0.017** | **0.009** | **0.025** | **0.010** |
| K06903 | 0.011 | 0.008 | 0.007 | 0.007 | 0.014 | 0.010 | 0.008 | 0.005 | **0.011** | **0.007** | **0.005** | **0.005** |
| K06905 | 0.008 | 0.006 | 0.006 | 0.005 | 0.010 | 0.008 | 0.008 | 0.005 | **0.011** | **0.007** | **0.007** | **0.005** |
| K06908 | 0.009 | 0.007 | 0.008 | 0.006 | 0.014 | 0.009 | 0.008 | 0.008 | **0.011** | **0.006** | **0.007** | **0.006** |
| K06915 | 0.006 | 0.007 | 0.011 | 0.013 | 0.003 | 0.004 | 0.003 | 0.003 | **0.003** | **0.003** | **0.007** | **0.005** |
| K06920 | 0.018 | 0.017 | 0.027 | 0.012 | 0.013 | 0.010 | 0.010 | 0.009 | **0.024** | **0.012** | **0.015** | **0.009** |
| K06923 | 0.045 | 0.013 | 0.039 | 0.016 | 0.063 | 0.016 | 0.058 | 0.016 | **0.051** | **0.013** | **0.063** | **0.016** |
| K06927 | 0.003 | 0.005 | 0.003 | 0.003 | 0.001 | 0.001 | 0.001 | 0.002 | **0.001** | **0.001** | **0.002** | **0.003** |
| K06929 | 0.010 | 0.013 | 0.009 | 0.008 | 0.006 | 0.006 | 0.003 | 0.004 | **0.007** | **0.007** | **0.003** | **0.003** |
| K06950 | 0.123 | 0.028 | 0.134 | 0.042 | 0.146 | 0.024 | 0.121 | 0.019 | **0.151** | **0.026** | **0.132** | **0.021** |
| K06957 | 0.002 | 0.004 | 0.000 | 0.001 | 0.000 | 0.001 | 0.000 | 0.000 | **0.003** | **0.004** | **0.000** | **0.000** |
| K06966 | 0.047 | 0.024 | 0.055 | 0.016 | 0.027 | 0.010 | 0.028 | 0.018 | **0.039** | **0.013** | **0.028** | **0.012** |
| K06973 | 0.046 | 0.021 | 0.033 | 0.020 | 0.053 | 0.015 | 0.059 | 0.013 | **0.047** | **0.016** | **0.062** | **0.015** |
| K06975 | 0.015 | 0.015 | 0.013 | 0.012 | 0.013 | 0.010 | 0.012 | 0.007 | **0.010** | **0.008** | **0.016** | **0.011** |
| K06978 | 0.009 | 0.012 | 0.009 | 0.009 | 0.005 | 0.006 | 0.009 | 0.011 | **0.005** | **0.004** | **0.010** | **0.007** |
| K06990 | 0.012 | 0.017 | 0.015 | 0.017 | 0.000 | 0.001 | 0.000 | 0.000 | **0.002** | **0.003** | **0.000** | **0.001** |
| K06996 | 0.004 | 0.006 | 0.006 | 0.009 | 0.000 | 0.001 | 0.001 | 0.001 | **0.000** | **0.001** | **0.003** | **0.004** |
| K07012 | 0.041 | 0.012 | 0.040 | 0.018 | 0.048 | 0.015 | 0.045 | 0.015 | **0.039** | **0.013** | **0.050** | **0.014** |
| K07037 | 0.023 | 0.015 | 0.037 | 0.019 | 0.021 | 0.012 | 0.009 | 0.010 | **0.025** | **0.012** | **0.013** | **0.009** |
| K07043 | 0.068 | 0.021 | 0.064 | 0.013 | 0.063 | 0.018 | 0.067 | 0.018 | **0.046** | **0.012** | **0.057** | **0.015** |
| K07052 | 0.097 | 0.024 | 0.092 | 0.027 | 0.124 | 0.027 | 0.104 | 0.044 | **0.114** | **0.024** | **0.096** | **0.023** |
| K07075 | 0.015 | 0.014 | 0.017 | 0.015 | 0.006 | 0.007 | 0.013 | 0.016 | **0.009** | **0.006** | **0.018** | **0.012** |
| K07078 | 0.009 | 0.010 | 0.003 | 0.003 | 0.005 | 0.005 | 0.014 | 0.011 | **0.004** | **0.004** | **0.012** | **0.010** |
| K07079 | 0.069 | 0.028 | 0.046 | 0.020 | 0.061 | 0.020 | 0.068 | 0.017 | **0.049** | **0.016** | **0.064** | **0.019** |
| K07091 | 0.009 | 0.017 | 0.008 | 0.013 | 0.000 | 0.001 | 0.000 | 0.000 | **0.003** | **0.005** | **0.001** | **0.001** |
| K07107 | 0.078 | 0.016 | 0.096 | 0.020 | 0.074 | 0.023 | 0.058 | 0.017 | **0.084** | **0.021** | **0.068** | **0.015** |
| K07113 | 0.002 | 0.003 | 0.003 | 0.006 | 0.000 | 0.001 | 0.000 | 0.000 | **0.002** | **0.003** | **0.000** | **0.000** |
| K07114 | **0.050** | **0.023** | **0.083** | **0.030** | 0.043 | 0.024 | 0.021 | 0.012 | **0.050** | **0.019** | **0.030** | **0.016** |
| K07121 | 0.002 | 0.003 | 0.002 | 0.004 | 0.000 | 0.001 | 0.000 | 0.000 | **0.003** | **0.004** | **0.000** | **0.001** |
| K07122 | 0.003 | 0.005 | 0.003 | 0.007 | 0.000 | 0.001 | 0.000 | 0.000 | **0.002** | **0.004** | **0.000** | **0.000** |
| K07148 | 0.004 | 0.007 | 0.003 | 0.004 | 0.006 | 0.009 | 0.004 | 0.006 | **0.002** | **0.004** | **0.011** | **0.011** |
| K07149 | 0.017 | 0.012 | 0.018 | 0.012 | 0.031 | 0.012 | 0.043 | 0.012 | **0.028** | **0.012** | **0.038** | **0.014** |
| K07152 | 0.007 | 0.011 | 0.013 | 0.021 | 0.000 | 0.001 | 0.000 | 0.000 | **0.003** | **0.005** | **0.000** | **0.001** |
| K07153 | 0.002 | 0.004 | 0.002 | 0.004 | 0.000 | 0.001 | 0.000 | 0.000 | **0.003** | **0.004** | **0.000** | **0.000** |
| K07164 | 0.016 | 0.017 | 0.032 | 0.018 | 0.014 | 0.011 | 0.006 | 0.008 | **0.019** | **0.010** | **0.010** | **0.009** |
| K07170 | **0.005** | **0.005** | **0.023** | **0.013** | 0.013 | 0.010 | 0.008 | 0.008 | **0.020** | **0.012** | **0.008** | **0.007** |
| K07175 | 0.015 | 0.013 | 0.024 | 0.023 | 0.003 | 0.003 | 0.004 | 0.004 | **0.005** | **0.005** | **0.009** | **0.008** |
| K07184 | 0.004 | 0.007 | 0.000 | 0.001 | 0.000 | 0.001 | 0.000 | 0.000 | **0.002** | **0.003** | **0.000** | **0.000** |
| K07185 | 0.025 | 0.013 | 0.016 | 0.009 | 0.018 | 0.009 | 0.027 | 0.015 | **0.021** | **0.010** | **0.029** | **0.013** |
| K07192 | 0.011 | 0.006 | 0.007 | 0.006 | 0.011 | 0.010 | 0.017 | 0.014 | **0.017** | **0.009** | **0.026** | **0.011** |
| K07213 | 0.009 | 0.008 | 0.016 | 0.012 | **0.014** | **0.011** | **0.001** | **0.002** | **0.017** | **0.011** | **0.004** | **0.005** |
| K07214 | 0.009 | 0.008 | 0.015 | 0.010 | 0.013 | 0.010 | 0.012 | 0.017 | **0.021** | **0.011** | **0.013** | **0.009** |
| K07221 | 0.003 | 0.005 | 0.010 | 0.008 | 0.007 | 0.010 | 0.002 | 0.004 | **0.014** | **0.009** | **0.006** | **0.006** |
| K07235 | 0.003 | 0.005 | 0.002 | 0.004 | 0.001 | 0.001 | 0.000 | 0.000 | **0.003** | **0.004** | **0.000** | **0.000** |
| K07236 | 0.003 | 0.006 | 0.002 | 0.004 | 0.000 | 0.001 | 0.000 | 0.000 | **0.002** | **0.004** | **0.000** | **0.000** |
| K07237 | 0.003 | 0.004 | 0.001 | 0.003 | 0.000 | 0.001 | 0.000 | 0.000 | **0.002** | **0.004** | **0.000** | **0.000** |
| K07248 | 0.007 | 0.005 | 0.011 | 0.009 | 0.011 | 0.011 | 0.017 | 0.008 | **0.012** | **0.007** | **0.019** | **0.011** |
| K07258 | 0.159 | 0.055 | 0.121 | 0.043 | 0.182 | 0.035 | 0.221 | 0.033 | **0.170** | **0.036** | **0.197** | **0.038** |
| K07259 | 0.020 | 0.015 | 0.046 | 0.026 | 0.021 | 0.020 | 0.015 | 0.014 | **0.042** | **0.018** | **0.017** | **0.012** |
| K07261 | 0.002 | 0.003 | 0.001 | 0.003 | 0.000 | 0.001 | 0.000 | 0.000 | **0.002** | **0.003** | **0.000** | **0.000** |
| K07263 | 0.013 | 0.012 | 0.030 | 0.018 | 0.025 | 0.019 | 0.006 | 0.009 | **0.034** | **0.018** | **0.012** | **0.010** |
| K07264 | 0.004 | 0.004 | 0.001 | 0.003 | 0.000 | 0.001 | 0.000 | 0.000 | **0.003** | **0.004** | **0.000** | **0.000** |
| K07270 | 0.008 | 0.018 | 0.002 | 0.004 | 0.002 | 0.005 | 0.000 | 0.000 | **0.003** | **0.004** | **0.000** | **0.000** |
| K07273 | 0.011 | 0.009 | 0.028 | 0.017 | 0.017 | 0.015 | 0.007 | 0.006 | **0.034** | **0.015** | **0.012** | **0.009** |
| K07277 | 0.026 | 0.021 | 0.040 | 0.014 | 0.017 | 0.013 | 0.004 | 0.009 | **0.026** | **0.012** | **0.012** | **0.009** |
| K07278 | 0.004 | 0.006 | 0.004 | 0.006 | 0.000 | 0.001 | 0.000 | 0.001 | **0.003** | **0.004** | **0.001** | **0.002** |
| K07287 | 0.003 | 0.003 | 0.004 | 0.007 | 0.000 | 0.001 | 0.000 | 0.000 | **0.003** | **0.004** | **0.001** | **0.001** |
| K07305 | 0.022 | 0.022 | 0.025 | 0.020 | 0.001 | 0.001 | 0.000 | 0.001 | **0.004** | **0.005** | **0.001** | **0.003** |
| K07306 | 0.006 | 0.010 | 0.002 | 0.005 | 0.001 | 0.001 | 0.000 | 0.001 | **0.005** | **0.006** | **0.001** | **0.002** |
| K07320 | 0.004 | 0.007 | 0.004 | 0.008 | 0.000 | 0.001 | 0.000 | 0.000 | **0.003** | **0.005** | **0.000** | **0.001** |
| K07323 | 0.009 | 0.013 | 0.009 | 0.014 | 0.000 | 0.001 | 0.000 | 0.001 | **0.004** | **0.005** | **0.001** | **0.002** |
| K07337 | 0.006 | 0.012 | 0.002 | 0.006 | 0.003 | 0.006 | 0.000 | 0.001 | **0.002** | **0.004** | **0.000** | **0.001** |
| K07345 | 0.004 | 0.006 | 0.003 | 0.006 | 0.001 | 0.003 | 0.000 | 0.000 | **0.003** | **0.004** | **0.000** | **0.001** |
| K07346 | 0.005 | 0.008 | 0.003 | 0.007 | 0.000 | 0.001 | 0.000 | 0.000 | **0.004** | **0.005** | **0.000** | **0.001** |
| K07347 | 0.008 | 0.010 | 0.006 | 0.013 | 0.002 | 0.004 | 0.000 | 0.000 | **0.005** | **0.006** | **0.000** | **0.001** |
| K07386 | 0.011 | 0.013 | 0.024 | 0.017 | 0.018 | 0.017 | 0.005 | 0.005 | **0.033** | **0.018** | **0.011** | **0.009** |
| K07387 | 0.004 | 0.003 | 0.009 | 0.008 | 0.011 | 0.011 | 0.002 | 0.004 | **0.015** | **0.008** | **0.007** | **0.007** |
| K07390 | 0.007 | 0.007 | 0.006 | 0.011 | 0.001 | 0.003 | 0.000 | 0.000 | **0.004** | **0.005** | **0.001** | **0.002** |
| K07400 | 0.003 | 0.005 | 0.002 | 0.004 | 0.000 | 0.001 | 0.000 | 0.000 | **0.003** | **0.004** | **0.000** | **0.000** |
| K07405 | 0.005 | 0.006 | 0.018 | 0.014 | 0.012 | 0.011 | 0.003 | 0.004 | **0.017** | **0.010** | **0.009** | **0.008** |
| K07407 | 0.084 | 0.030 | 0.069 | 0.029 | 0.094 | 0.023 | 0.107 | 0.025 | **0.095** | **0.019** | **0.110** | **0.020** |
| K07445 | 0.000 | 0.000 | 0.000 | 0.000 | 0.000 | 0.000 | 0.001 | 0.003 | **0.002** | **0.003** | **0.000** | **0.001** |
| K07464 | 0.038 | 0.018 | 0.030 | 0.020 | 0.032 | 0.009 | 0.029 | 0.016 | **0.027** | **0.010** | **0.035** | **0.011** |
| K07479 | 0.003 | 0.006 | 0.000 | 0.001 | 0.000 | 0.001 | 0.000 | 0.000 | **0.002** | **0.003** | **0.000** | **0.000** |
| K07480 | 0.007 | 0.015 | 0.001 | 0.002 | 0.004 | 0.010 | 0.000 | 0.001 | **0.005** | **0.008** | **0.000** | **0.001** |
| K07483 | 0.110 | 0.046 | 0.087 | 0.034 | 0.174 | 0.070 | 0.145 | 0.047 | **0.132** | **0.031** | **0.106** | **0.033** |
| K07485 | **0.006** | **0.010** | **0.000** | **0.001** | 0.000 | 0.001 | 0.000 | 0.000 | 0.001 | 0.003 | 0.000 | 0.001 |
| K07487 | 0.000 | 0.001 | 0.002 | 0.003 | 0.000 | 0.001 | 0.000 | 0.000 | **0.001** | **0.002** | **0.000** | **0.000** |
| K07495 | 0.002 | 0.007 | 0.000 | 0.002 | 0.000 | 0.001 | 0.001 | 0.003 | **0.001** | **0.003** | **0.005** | **0.014** |
| K07496 | 0.032 | 0.020 | 0.026 | 0.017 | 0.037 | 0.014 | 0.046 | 0.015 | **0.033** | **0.016** | **0.042** | **0.015** |
| K07502 | 0.019 | 0.012 | 0.024 | 0.019 | 0.023 | 0.010 | 0.032 | 0.015 | **0.021** | **0.011** | **0.029** | **0.013** |
| K07507 | 0.037 | 0.014 | 0.047 | 0.013 | 0.031 | 0.015 | 0.019 | 0.012 | **0.035** | **0.012** | **0.022** | **0.012** |
| K07552 | 0.013 | 0.013 | 0.022 | 0.030 | 0.005 | 0.006 | 0.002 | 0.003 | **0.007** | **0.006** | **0.003** | **0.004** |
| K07568 | 0.075 | 0.018 | 0.084 | 0.022 | 0.085 | 0.017 | 0.072 | 0.017 | **0.091** | **0.021** | **0.072** | **0.017** |
| K07590 | 0.010 | 0.010 | 0.006 | 0.006 | 0.013 | 0.008 | 0.006 | 0.005 | **0.012** | **0.007** | **0.006** | **0.005** |
| K07646 | 0.049 | 0.019 | 0.037 | 0.014 | 0.052 | 0.015 | 0.057 | 0.016 | **0.038** | **0.014** | **0.053** | **0.017** |
| K07648 | 0.002 | 0.004 | 0.000 | 0.001 | 0.001 | 0.002 | 0.000 | 0.000 | **0.003** | **0.004** | **0.000** | **0.000** |
| K07652 | 0.009 | 0.007 | 0.005 | 0.005 | 0.016 | 0.007 | 0.009 | 0.006 | **0.009** | **0.007** | **0.005** | **0.005** |
| K07662 | 0.002 | 0.004 | 0.000 | 0.002 | 0.000 | 0.001 | 0.000 | 0.001 | **0.003** | **0.004** | **0.000** | **0.001** |
| K07665 | 0.011 | 0.009 | 0.015 | 0.015 | 0.007 | 0.005 | 0.007 | 0.005 | **0.009** | **0.007** | **0.014** | **0.008** |
| K07687 | 0.002 | 0.003 | 0.003 | 0.006 | 0.000 | 0.001 | 0.000 | 0.000 | **0.002** | **0.004** | **0.000** | **0.001** |
| K07699 | 0.048 | 0.022 | 0.036 | 0.018 | 0.059 | 0.013 | 0.077 | 0.019 | **0.052** | **0.013** | **0.068** | **0.017** |
| K07713 | 0.004 | 0.005 | 0.002 | 0.002 | 0.001 | 0.002 | 0.005 | 0.007 | **0.003** | **0.004** | **0.011** | **0.010** |
| K07720 | 0.144 | 0.048 | 0.123 | 0.055 | 0.178 | 0.038 | 0.204 | 0.048 | **0.159** | **0.040** | **0.184** | **0.041** |
| K07724 | 0.002 | 0.004 | 0.001 | 0.002 | 0.000 | 0.001 | 0.000 | 0.000 | **0.002** | **0.003** | **0.000** | **0.001** |
| K07726 | 0.030 | 0.017 | 0.019 | 0.008 | 0.032 | 0.009 | 0.037 | 0.014 | **0.027** | **0.011** | **0.036** | **0.013** |
| K07733 | 0.005 | 0.010 | 0.007 | 0.014 | 0.000 | 0.001 | 0.000 | 0.001 | **0.003** | **0.004** | **0.000** | **0.000** |
| K07751 | 0.002 | 0.003 | 0.000 | 0.001 | 0.001 | 0.002 | 0.000 | 0.000 | **0.002** | **0.004** | **0.000** | **0.000** |
| K07773 | 0.003 | 0.005 | 0.000 | 0.001 | 0.000 | 0.001 | 0.000 | 0.000 | **0.003** | **0.004** | **0.000** | **0.001** |
| K07783 | 0.001 | 0.002 | 0.000 | 0.001 | 0.000 | 0.001 | 0.001 | 0.002 | **0.001** | **0.003** | **0.004** | **0.004** |
| K07791 | 0.007 | 0.013 | 0.009 | 0.008 | 0.006 | 0.008 | 0.001 | 0.002 | **0.014** | **0.009** | **0.005** | **0.006** |
| K08070 | 0.015 | 0.008 | 0.008 | 0.007 | 0.022 | 0.008 | 0.011 | 0.005 | **0.014** | **0.008** | **0.009** | **0.006** |
| K08138 | 0.001 | 0.003 | 0.001 | 0.002 | 0.001 | 0.002 | 0.005 | 0.009 | **0.001** | **0.002** | **0.007** | **0.007** |
| K08156 | 0.005 | 0.010 | 0.009 | 0.013 | 0.004 | 0.005 | 0.007 | 0.006 | **0.004** | **0.005** | **0.009** | **0.008** |
| K08159 | 0.005 | 0.008 | 0.005 | 0.009 | 0.001 | 0.002 | 0.001 | 0.002 | **0.004** | **0.004** | **0.002** | **0.003** |
| K08161 | 0.005 | 0.010 | 0.001 | 0.003 | 0.000 | 0.001 | 0.000 | 0.001 | **0.003** | **0.004** | **0.000** | **0.001** |
| K08169 | 0.001 | 0.003 | 0.003 | 0.007 | 0.000 | 0.001 | 0.002 | 0.004 | **0.001** | **0.002** | **0.005** | **0.005** |
| K08218 | 0.016 | 0.015 | 0.038 | 0.022 | 0.025 | 0.025 | 0.007 | 0.010 | **0.048** | **0.023** | **0.016** | **0.013** |
| K08223 | 0.005 | 0.006 | 0.005 | 0.006 | 0.001 | 0.002 | 0.002 | 0.004 | **0.002** | **0.002** | **0.007** | **0.007** |
| K08234 | 0.027 | 0.013 | 0.025 | 0.013 | 0.033 | 0.014 | 0.057 | 0.020 | **0.038** | **0.014** | **0.052** | **0.018** |
| K08281 | 0.031 | 0.030 | 0.053 | 0.036 | 0.011 | 0.010 | 0.007 | 0.007 | **0.023** | **0.014** | **0.006** | **0.007** |
| K08289 | 0.009 | 0.007 | 0.018 | 0.011 | 0.010 | 0.009 | 0.007 | 0.007 | **0.021** | **0.011** | **0.010** | **0.007** |
| K08296 | 0.013 | 0.016 | 0.009 | 0.014 | 0.001 | 0.002 | 0.003 | 0.004 | **0.006** | **0.005** | **0.002** | **0.003** |
| K08300 | 0.006 | 0.009 | 0.007 | 0.011 | 0.000 | 0.001 | 0.001 | 0.002 | **0.005** | **0.005** | **0.001** | **0.002** |
| K08302 | 0.015 | 0.015 | 0.005 | 0.004 | 0.008 | 0.005 | 0.013 | 0.008 | **0.008** | **0.006** | **0.014** | **0.008** |
| K08304 | 0.003 | 0.004 | 0.005 | 0.008 | 0.000 | 0.001 | 0.000 | 0.001 | **0.003** | **0.004** | **0.001** | **0.002** |
| K08305 | 0.004 | 0.005 | 0.006 | 0.010 | 0.000 | 0.001 | 0.000 | 0.001 | **0.003** | **0.004** | **0.000** | **0.001** |
| K08306 | 0.004 | 0.006 | 0.002 | 0.006 | 0.000 | 0.001 | 0.000 | 0.000 | **0.004** | **0.005** | **0.000** | **0.001** |
| K08307 | 0.020 | 0.019 | 0.034 | 0.018 | 0.014 | 0.011 | 0.005 | 0.006 | **0.022** | **0.010** | **0.009** | **0.007** |
| K08311 | 0.009 | 0.010 | 0.010 | 0.013 | 0.001 | 0.001 | 0.000 | 0.001 | **0.004** | **0.004** | **0.001** | **0.003** |
| K08312 | 0.003 | 0.005 | 0.001 | 0.003 | 0.000 | 0.001 | 0.000 | 0.000 | **0.003** | **0.004** | **0.000** | **0.000** |
| K08325 | 0.001 | 0.002 | 0.001 | 0.003 | 0.000 | 0.001 | 0.002 | 0.003 | **0.001** | **0.003** | **0.006** | **0.007** |
| K08369 | 0.002 | 0.003 | 0.006 | 0.006 | 0.003 | 0.004 | 0.007 | 0.007 | **0.003** | **0.005** | **0.007** | **0.006** |
| K08384 | 0.059 | 0.030 | 0.042 | 0.027 | 0.074 | 0.020 | 0.087 | 0.020 | **0.070** | **0.018** | **0.083** | **0.019** |
| K08591 | 0.078 | 0.031 | 0.058 | 0.023 | 0.087 | 0.017 | 0.097 | 0.021 | **0.086** | **0.018** | **0.099** | **0.020** |
| K08600 | 0.082 | 0.039 | 0.061 | 0.034 | 0.100 | 0.033 | 0.121 | 0.027 | **0.092** | **0.025** | **0.128** | **0.027** |
| K08641 | 0.005 | 0.005 | 0.015 | 0.010 | 0.011 | 0.009 | 0.008 | 0.007 | **0.018** | **0.010** | **0.010** | **0.008** |
| K08676 | 0.001 | 0.003 | 0.001 | 0.003 | 0.000 | 0.000 | 0.003 | 0.005 | **0.001** | **0.002** | **0.007** | **0.007** |
| K08961 | 0.000 | 0.002 | 0.000 | 0.001 | 0.000 | 0.001 | 0.004 | 0.006 | **0.001** | **0.002** | **0.009** | **0.008** |
| K08981 | 0.003 | 0.005 | 0.002 | 0.003 | 0.000 | 0.001 | 0.003 | 0.005 | **0.001** | **0.002** | **0.004** | **0.005** |
| K08986 | 0.001 | 0.003 | 0.001 | 0.002 | 0.002 | 0.005 | 0.000 | 0.001 | **0.002** | **0.003** | **0.000** | **0.001** |
| K08992 | 0.003 | 0.005 | 0.000 | 0.001 | 0.000 | 0.001 | 0.000 | 0.000 | **0.003** | **0.005** | **0.000** | **0.000** |
| K08999 | 0.015 | 0.013 | 0.028 | 0.016 | 0.008 | 0.011 | 0.005 | 0.007 | **0.016** | **0.009** | **0.008** | **0.007** |
| K09005 | 0.012 | 0.012 | 0.019 | 0.017 | 0.003 | 0.005 | 0.000 | 0.000 | **0.003** | **0.004** | **0.001** | **0.001** |
| K09013 | 0.051 | 0.016 | 0.057 | 0.018 | 0.049 | 0.014 | 0.038 | 0.018 | **0.053** | **0.016** | **0.040** | **0.015** |
| K09014 | 0.023 | 0.015 | 0.039 | 0.018 | 0.020 | 0.012 | 0.011 | 0.008 | **0.026** | **0.013** | **0.015** | **0.011** |
| K09015 | 0.010 | 0.007 | 0.020 | 0.011 | 0.013 | 0.014 | 0.007 | 0.005 | **0.020** | **0.011** | **0.011** | **0.008** |
| K09125 | 0.017 | 0.012 | 0.032 | 0.017 | 0.010 | 0.011 | 0.005 | 0.007 | **0.018** | **0.012** | **0.009** | **0.007** |
| K09132 | 0.001 | 0.001 | 0.001 | 0.002 | 0.000 | 0.001 | 0.002 | 0.003 | **0.000** | **0.001** | **0.002** | **0.004** |
| K09136 | 0.003 | 0.005 | 0.003 | 0.005 | 0.001 | 0.001 | 0.000 | 0.001 | **0.003** | **0.004** | **0.000** | **0.001** |
| K09155 | 0.018 | 0.013 | 0.031 | 0.018 | **0.017** | **0.013** | **0.001** | **0.002** | **0.023** | **0.012** | **0.007** | **0.006** |
| K09158 | 0.005 | 0.007 | 0.005 | 0.009 | 0.001 | 0.001 | 0.000 | 0.000 | **0.002** | **0.003** | **0.001** | **0.001** |
| K09159 | 0.006 | 0.012 | 0.005 | 0.010 | 0.000 | 0.001 | 0.000 | 0.000 | **0.004** | **0.005** | **0.000** | **0.001** |
| K09457 | 0.016 | 0.012 | 0.021 | 0.014 | 0.012 | 0.009 | 0.011 | 0.008 | **0.020** | **0.009** | **0.014** | **0.008** |
| K09474 | **0.002** | **0.002** | **0.014** | **0.009** | 0.008 | 0.009 | 0.002 | 0.004 | **0.015** | **0.012** | **0.003** | **0.004** |
| K09516 | 0.004 | 0.008 | 0.016 | 0.010 | 0.008 | 0.014 | 0.001 | 0.002 | **0.014** | **0.009** | **0.004** | **0.005** |
| K09680 | 0.004 | 0.006 | 0.014 | 0.011 | 0.007 | 0.009 | 0.004 | 0.007 | **0.013** | **0.009** | **0.006** | **0.007** |
| K09687 | 0.362 | 0.106 | 0.292 | 0.087 | 0.416 | 0.060 | 0.440 | 0.056 | **0.366** | **0.055** | **0.415** | **0.054** |
| K09690 | 0.037 | 0.016 | 0.028 | 0.012 | 0.040 | 0.017 | 0.055 | 0.016 | **0.037** | **0.013** | **0.052** | **0.015** |
| K09691 | 0.044 | 0.025 | 0.038 | 0.019 | 0.058 | 0.025 | 0.075 | 0.022 | **0.056** | **0.018** | **0.073** | **0.019** |
| K09698 | 0.004 | 0.005 | 0.003 | 0.003 | 0.003 | 0.004 | 0.002 | 0.002 | **0.005** | **0.005** | **0.002** | **0.003** |
| K09702 | 0.009 | 0.007 | 0.004 | 0.004 | 0.006 | 0.006 | 0.018 | 0.012 | **0.007** | **0.005** | **0.013** | **0.009** |
| K09748 | 0.068 | 0.018 | 0.076 | 0.021 | 0.077 | 0.022 | 0.067 | 0.013 | **0.076** | **0.016** | **0.063** | **0.015** |
| K09758 | 0.006 | 0.006 | 0.001 | 0.003 | 0.001 | 0.002 | 0.003 | 0.004 | **0.001** | **0.002** | **0.005** | **0.006** |
| K09760 | 0.024 | 0.012 | 0.033 | 0.016 | 0.023 | 0.015 | 0.014 | 0.011 | **0.032** | **0.013** | **0.022** | **0.013** |
| K09765 | 0.059 | 0.015 | 0.049 | 0.019 | 0.062 | 0.016 | 0.067 | 0.011 | **0.055** | **0.013** | **0.067** | **0.015** |
| K09767 | 0.011 | 0.011 | 0.010 | 0.012 | 0.011 | 0.008 | 0.002 | 0.002 | **0.009** | **0.007** | **0.003** | **0.004** |
| K09772 | 0.054 | 0.023 | 0.036 | 0.020 | 0.064 | 0.013 | 0.076 | 0.018 | **0.057** | **0.016** | **0.069** | **0.016** |
| K09773 | 0.010 | 0.008 | 0.007 | 0.010 | 0.002 | 0.003 | 0.001 | 0.001 | **0.005** | **0.005** | **0.002** | **0.003** |
| K09774 | 0.017 | 0.022 | 0.024 | 0.022 | 0.003 | 0.005 | 0.000 | 0.001 | **0.005** | **0.005** | **0.001** | **0.001** |
| K09789 | 0.007 | 0.010 | 0.004 | 0.006 | 0.006 | 0.010 | 0.003 | 0.006 | **0.002** | **0.005** | **0.007** | **0.007** |
| K09790 | 0.026 | 0.015 | 0.034 | 0.016 | 0.023 | 0.008 | 0.033 | 0.014 | **0.023** | **0.010** | **0.034** | **0.012** |
| K09791 | 0.004 | 0.006 | 0.005 | 0.008 | 0.000 | 0.001 | 0.000 | 0.000 | **0.003** | **0.004** | **0.001** | **0.001** |
| K09793 | 0.003 | 0.003 | 0.003 | 0.004 | 0.005 | 0.008 | 0.002 | 0.004 | **0.003** | **0.004** | **0.007** | **0.007** |
| K09798 | 0.017 | 0.024 | 0.020 | 0.020 | 0.002 | 0.005 | 0.000 | 0.000 | **0.003** | **0.004** | **0.001** | **0.002** |
| K09800 | 0.006 | 0.010 | 0.005 | 0.008 | 0.001 | 0.002 | 0.000 | 0.001 | **0.004** | **0.004** | **0.001** | **0.002** |
| K09802 | 0.002 | 0.002 | 0.005 | 0.004 | 0.005 | 0.006 | 0.000 | 0.001 | **0.014** | **0.010** | **0.001** | **0.003** |
| K09803 | 0.004 | 0.006 | 0.004 | 0.004 | 0.003 | 0.004 | 0.004 | 0.007 | **0.005** | **0.004** | **0.002** | **0.003** |
| K09808 | 0.039 | 0.028 | 0.073 | 0.029 | 0.032 | 0.025 | 0.009 | 0.012 | **0.042** | **0.017** | **0.014** | **0.013** |
| K09809 | 0.000 | 0.001 | 0.000 | 0.001 | 0.001 | 0.002 | 0.002 | 0.003 | **0.001** | **0.002** | **0.003** | **0.004** |
| K09810 | 0.036 | 0.026 | 0.056 | 0.029 | 0.017 | 0.016 | 0.006 | 0.007 | **0.026** | **0.012** | **0.011** | **0.008** |
| K09858 | 0.004 | 0.006 | 0.006 | 0.011 | 0.000 | 0.001 | 0.000 | 0.000 | **0.003** | **0.005** | **0.001** | **0.001** |
| K09859 | 0.002 | 0.006 | 0.001 | 0.002 | 0.002 | 0.006 | 0.000 | 0.001 | **0.001** | **0.002** | **0.000** | **0.000** |
| K09862 | 0.003 | 0.006 | 0.006 | 0.011 | 0.000 | 0.002 | 0.000 | 0.000 | **0.002** | **0.003** | **0.000** | **0.001** |
| K09891 | 0.001 | 0.003 | 0.000 | 0.001 | 0.000 | 0.001 | 0.000 | 0.001 | **0.003** | **0.004** | **0.000** | **0.000** |
| K09892 | 0.001 | 0.002 | 0.000 | 0.001 | 0.000 | 0.001 | 0.000 | 0.000 | **0.003** | **0.004** | **0.000** | **0.001** |
| K09893 | 0.004 | 0.007 | 0.001 | 0.002 | 0.000 | 0.001 | 0.000 | 0.000 | **0.003** | **0.004** | **0.000** | **0.000** |
| K09897 | 0.001 | 0.003 | 0.000 | 0.001 | 0.000 | 0.001 | 0.000 | 0.000 | **0.002** | **0.004** | **0.000** | **0.000** |
| K09899 | 0.002 | 0.005 | 0.000 | 0.001 | 0.000 | 0.000 | 0.000 | 0.000 | **0.003** | **0.003** | **0.000** | **0.000** |
| K09902 | 0.002 | 0.005 | 0.002 | 0.005 | 0.000 | 0.001 | 0.000 | 0.000 | **0.002** | **0.003** | **0.000** | **0.000** |
| K09906 | 0.002 | 0.006 | 0.001 | 0.002 | 0.000 | 0.001 | 0.000 | 0.000 | **0.002** | **0.003** | **0.000** | **0.000** |
| K09908 | 0.003 | 0.006 | 0.002 | 0.005 | 0.000 | 0.001 | 0.000 | 0.000 | **0.002** | **0.004** | **0.000** | **0.000** |
| K09911 | 0.003 | 0.005 | 0.000 | 0.001 | 0.000 | 0.001 | 0.000 | 0.000 | **0.003** | **0.005** | **0.000** | **0.000** |
| K09922 | 0.004 | 0.005 | 0.008 | 0.008 | 0.005 | 0.006 | 0.003 | 0.004 | **0.003** | **0.005** | **0.007** | **0.007** |
| K09939 | 0.001 | 0.002 | 0.004 | 0.004 | 0.005 | 0.007 | 0.002 | 0.003 | **0.002** | **0.003** | **0.005** | **0.006** |
| K09951 | 0.024 | 0.013 | 0.030 | 0.015 | 0.039 | 0.016 | 0.027 | 0.015 | **0.041** | **0.011** | **0.032** | **0.011** |
| K09952 | 0.005 | 0.005 | 0.010 | 0.010 | 0.008 | 0.008 | 0.007 | 0.006 | **0.016** | **0.010** | **0.004** | **0.005** |
| K09955 | 0.054 | 0.025 | 0.035 | 0.016 | 0.027 | 0.016 | 0.055 | 0.029 | **0.032** | **0.014** | **0.059** | **0.021** |
| K09963 | 0.008 | 0.006 | 0.009 | 0.006 | 0.005 | 0.005 | 0.005 | 0.003 | **0.009** | **0.006** | **0.004** | **0.004** |
| K09979 | 0.005 | 0.009 | 0.005 | 0.010 | 0.005 | 0.006 | 0.000 | 0.000 | **0.003** | **0.004** | **0.001** | **0.002** |
| K09997 | 0.001 | 0.002 | 0.000 | 0.001 | 0.000 | 0.001 | 0.000 | 0.000 | **0.003** | **0.004** | **0.000** | **0.001** |
| K09998 | 0.003 | 0.004 | 0.000 | 0.001 | 0.000 | 0.001 | 0.000 | 0.000 | **0.003** | **0.004** | **0.000** | **0.000** |
| K09999 | 0.002 | 0.004 | 0.000 | 0.001 | 0.000 | 0.001 | 0.000 | 0.000 | **0.003** | **0.004** | **0.000** | **0.000** |
| K10000 | 0.003 | 0.004 | 0.000 | 0.001 | 0.000 | 0.001 | 0.000 | 0.000 | **0.003** | **0.005** | **0.000** | **0.000** |
| K10011 | 0.003 | 0.005 | 0.002 | 0.006 | 0.001 | 0.002 | 0.000 | 0.001 | **0.003** | **0.005** | **0.000** | **0.001** |
| K10036 | 0.006 | 0.007 | 0.006 | 0.004 | 0.005 | 0.006 | 0.004 | 0.004 | **0.013** | **0.008** | **0.007** | **0.006** |
| K10117 | 0.034 | 0.020 | 0.022 | 0.015 | 0.033 | 0.015 | 0.049 | 0.015 | **0.038** | **0.011** | **0.051** | **0.015** |
| K10118 | 0.042 | 0.031 | 0.031 | 0.020 | 0.040 | 0.016 | 0.061 | 0.020 | **0.039** | **0.013** | **0.063** | **0.021** |
| K10119 | 0.049 | 0.022 | 0.040 | 0.021 | 0.044 | 0.019 | 0.071 | 0.026 | **0.047** | **0.016** | **0.063** | **0.018** |
| K10189 | 0.031 | 0.018 | 0.026 | 0.015 | 0.024 | 0.009 | 0.033 | 0.011 | **0.023** | **0.010** | **0.031** | **0.012** |
| K10190 | 0.038 | 0.016 | 0.034 | 0.020 | 0.043 | 0.015 | 0.056 | 0.014 | **0.035** | **0.012** | **0.047** | **0.014** |
| K10206 | 0.065 | 0.024 | 0.055 | 0.024 | 0.062 | 0.019 | 0.074 | 0.017 | **0.063** | **0.016** | **0.079** | **0.015** |
| K10212 | 0.005 | 0.010 | 0.001 | 0.002 | 0.004 | 0.004 | 0.012 | 0.010 | **0.002** | **0.004** | **0.005** | **0.006** |
| K10439 | 0.128 | 0.048 | 0.165 | 0.063 | 0.157 | 0.043 | 0.173 | 0.048 | **0.135** | **0.040** | **0.170** | **0.048** |
| K10560 | 0.000 | 0.002 | 0.002 | 0.003 | 0.001 | 0.003 | 0.001 | 0.002 | **0.001** | **0.002** | **0.004** | **0.004** |
| K10563 | 0.044 | 0.021 | 0.052 | 0.019 | 0.031 | 0.011 | 0.023 | 0.017 | **0.032** | **0.013** | **0.021** | **0.010** |
| K10710 | 0.007 | 0.006 | 0.006 | 0.007 | 0.003 | 0.003 | 0.003 | 0.005 | **0.007** | **0.005** | **0.013** | **0.009** |
| K10763 | 0.003 | 0.005 | 0.005 | 0.009 | 0.000 | 0.001 | 0.000 | 0.001 | **0.003** | **0.004** | **0.000** | **0.001** |
| K10783 | 0.009 | 0.013 | 0.014 | 0.018 | 0.001 | 0.002 | 0.002 | 0.004 | **0.001** | **0.002** | **0.003** | **0.004** |
| K10806 | 0.007 | 0.012 | 0.007 | 0.012 | 0.001 | 0.002 | 0.000 | 0.000 | **0.003** | **0.004** | **0.001** | **0.001** |
| K10947 | 0.090 | 0.033 | 0.059 | 0.028 | 0.107 | 0.022 | 0.117 | 0.025 | **0.093** | **0.019** | **0.112** | **0.023** |
| K10984 | 0.001 | 0.003 | 0.001 | 0.003 | 0.000 | 0.001 | 0.000 | 0.001 | **0.001** | **0.002** | **0.000** | **0.001** |
| K11066 | 0.001 | 0.002 | 0.002 | 0.004 | 0.000 | 0.001 | 0.000 | 0.000 | **0.003** | **0.004** | **0.000** | **0.000** |
| K11068 | 0.060 | 0.021 | 0.057 | 0.018 | 0.058 | 0.013 | 0.068 | 0.016 | **0.053** | **0.013** | **0.064** | **0.014** |
| K11071 | 0.057 | 0.018 | 0.054 | 0.023 | 0.073 | 0.020 | 0.073 | 0.016 | **0.059** | **0.014** | **0.072** | **0.018** |
| K11073 | 0.003 | 0.004 | 0.007 | 0.017 | 0.000 | 0.001 | 0.000 | 0.000 | **0.003** | **0.005** | **0.000** | **0.000** |
| K11085 | 0.019 | 0.014 | 0.027 | 0.014 | 0.014 | 0.011 | 0.004 | 0.005 | **0.022** | **0.011** | **0.010** | **0.008** |
| K11105 | 0.021 | 0.016 | 0.011 | 0.010 | 0.015 | 0.009 | 0.020 | 0.016 | **0.019** | **0.010** | **0.027** | **0.011** |
| K11107 | 0.003 | 0.006 | 0.000 | 0.001 | 0.000 | 0.001 | 0.000 | 0.000 | **0.003** | **0.004** | **0.000** | **0.000** |
| K11179 | 0.004 | 0.006 | 0.003 | 0.006 | 0.001 | 0.001 | 0.000 | 0.000 | **0.003** | **0.004** | **0.000** | **0.001** |
| K11381 | 0.012 | 0.019 | 0.015 | 0.017 | 0.000 | 0.001 | 0.002 | 0.003 | **0.000** | **0.001** | **0.003** | **0.003** |
| K11527 | 0.001 | 0.003 | 0.002 | 0.003 | 0.000 | 0.001 | 0.003 | 0.005 | **0.001** | **0.001** | **0.008** | **0.007** |
| K11537 | 0.001 | 0.003 | 0.001 | 0.002 | 0.000 | 0.001 | 0.004 | 0.005 | **0.001** | **0.002** | **0.007** | **0.006** |
| K11604 | 0.002 | 0.003 | 0.001 | 0.002 | 0.001 | 0.002 | 0.000 | 0.000 | **0.003** | **0.005** | **0.001** | **0.003** |
| K11605 | 0.002 | 0.004 | 0.001 | 0.002 | 0.000 | 0.000 | 0.000 | 0.001 | **0.003** | **0.005** | **0.001** | **0.002** |
| K11606 | 0.003 | 0.007 | 0.001 | 0.002 | 0.000 | 0.001 | 0.000 | 0.001 | **0.003** | **0.004** | **0.001** | **0.002** |
| K11607 | 0.002 | 0.004 | 0.001 | 0.002 | 0.000 | 0.001 | 0.000 | 0.001 | **0.002** | **0.003** | **0.001** | **0.002** |
| K11688 | 0.010 | 0.008 | 0.009 | 0.011 | 0.011 | 0.007 | 0.004 | 0.005 | **0.009** | **0.006** | **0.005** | **0.004** |
| K11717 | 0.079 | 0.019 | 0.095 | 0.024 | 0.061 | 0.028 | 0.044 | 0.019 | **0.076** | **0.021** | **0.049** | **0.017** |
| K11719 | 0.004 | 0.009 | 0.006 | 0.013 | 0.001 | 0.001 | 0.000 | 0.000 | **0.002** | **0.003** | **0.000** | **0.001** |
| K11720 | 0.007 | 0.012 | 0.008 | 0.014 | 0.000 | 0.001 | 0.000 | 0.001 | **0.003** | **0.004** | **0.001** | **0.001** |
| K11747 | 0.003 | 0.005 | 0.003 | 0.006 | 0.000 | 0.001 | 0.000 | 0.000 | **0.002** | **0.004** | **0.000** | **0.000** |
| K11749 | 0.070 | 0.017 | 0.076 | 0.016 | 0.070 | 0.014 | 0.062 | 0.022 | **0.071** | **0.016** | **0.061** | **0.015** |
| K11751 | 0.013 | 0.016 | 0.014 | 0.018 | 0.000 | 0.001 | 0.000 | 0.000 | **0.004** | **0.005** | **0.000** | **0.001** |
| K11752 | 0.093 | 0.025 | 0.110 | 0.024 | 0.105 | 0.023 | 0.094 | 0.029 | **0.117** | **0.023** | **0.090** | **0.022** |
| K11896 | 0.005 | 0.007 | 0.005 | 0.010 | 0.000 | 0.001 | 0.000 | 0.000 | **0.003** | **0.005** | **0.000** | **0.001** |
| K11904 | 0.003 | 0.005 | 0.003 | 0.007 | 0.000 | 0.001 | 0.000 | 0.000 | **0.003** | **0.004** | **0.000** | **0.001** |
| K11910 | 0.001 | 0.002 | 0.001 | 0.003 | 0.000 | 0.001 | 0.000 | 0.000 | **0.001** | **0.003** | **0.000** | **0.000** |
| K11934 | 0.001 | 0.003 | 0.001 | 0.003 | 0.000 | 0.001 | 0.003 | 0.006 | **0.001** | **0.002** | **0.006** | **0.006** |
| K11991 | 0.002 | 0.003 | 0.001 | 0.003 | 0.000 | 0.001 | 0.000 | 0.000 | **0.003** | **0.004** | **0.000** | **0.000** |
| K12056 | 0.003 | 0.009 | 0.002 | 0.004 | 0.000 | 0.001 | 0.000 | 0.000 | **0.001** | **0.002** | **0.000** | **0.000** |
| K12150 | 0.002 | 0.004 | 0.000 | 0.001 | 0.000 | 0.001 | 0.000 | 0.000 | **0.003** | **0.004** | **0.000** | **0.001** |
| K12267 | 0.046 | 0.015 | 0.061 | 0.024 | **0.055** | **0.023** | **0.020** | **0.012** | **0.056** | **0.021** | **0.027** | **0.015** |
| K12297 | 0.003 | 0.006 | 0.002 | 0.004 | 0.001 | 0.002 | 0.000 | 0.001 | **0.003** | **0.004** | **0.000** | **0.001** |
| K12343 | 0.000 | 0.000 | 0.000 | 0.001 | 0.000 | 0.000 | 0.003 | 0.007 | **0.000** | **0.001** | **0.004** | **0.005** |
| K12344 | 0.001 | 0.003 | 0.000 | 0.002 | 0.000 | 0.000 | 0.000 | 0.001 | **0.000** | **0.001** | **0.003** | **0.003** |
| K12368 | 0.004 | 0.005 | 0.004 | 0.009 | 0.001 | 0.001 | 0.000 | 0.000 | **0.003** | **0.004** | **0.000** | **0.000** |
| K12524 | 0.022 | 0.021 | 0.034 | 0.013 | 0.020 | 0.018 | 0.008 | 0.013 | **0.028** | **0.014** | **0.014** | **0.012** |
| K12525 | 0.003 | 0.006 | 0.000 | 0.002 | 0.000 | 0.001 | 0.000 | 0.000 | **0.003** | **0.004** | **0.000** | **0.001** |
| K12554 | 0.001 | 0.001 | 0.000 | 0.001 | **0.000** | **0.000** | **0.003** | **0.003** | 0.001 | 0.003 | 0.001 | 0.002 |
| K12573 | 0.064 | 0.019 | 0.063 | 0.024 | 0.077 | 0.016 | 0.067 | 0.021 | **0.076** | **0.014** | **0.064** | **0.015** |
| K12942 | 0.011 | 0.010 | 0.019 | 0.014 | 0.012 | 0.012 | 0.006 | 0.005 | **0.021** | **0.010** | **0.011** | **0.008** |
| K12984 | 0.002 | 0.004 | 0.000 | 0.001 | 0.000 | 0.001 | 0.000 | 0.000 | **0.003** | **0.004** | **0.000** | **0.000** |
| K13002 | 0.000 | 0.001 | 0.000 | 0.002 | 0.000 | 0.000 | 0.001 | 0.004 | **0.000** | **0.001** | **0.003** | **0.004** |
| K13016 | 0.000 | 0.001 | 0.000 | 0.001 | 0.000 | 0.000 | 0.001 | 0.002 | **0.000** | **0.000** | **0.001** | **0.002** |
| K13043 | 0.001 | 0.002 | 0.003 | 0.003 | 0.001 | 0.002 | 0.005 | 0.008 | **0.001** | **0.002** | **0.007** | **0.007** |
| K13051 | 0.003 | 0.006 | 0.004 | 0.007 | 0.001 | 0.001 | 0.004 | 0.004 | **0.001** | **0.002** | **0.004** | **0.004** |
| K13378 | 0.008 | 0.008 | 0.017 | 0.010 | 0.013 | 0.012 | 0.004 | 0.006 | **0.022** | **0.012** | **0.008** | **0.007** |
| K13498 | 0.012 | 0.013 | 0.014 | 0.017 | 0.005 | 0.012 | 0.001 | 0.001 | **0.004** | **0.005** | **0.001** | **0.001** |
| K13570 | 0.000 | 0.001 | 0.000 | 0.001 | **0.000** | **0.000** | **0.003** | **0.002** | 0.001 | 0.003 | 0.001 | 0.002 |
| K13634 | 0.003 | 0.004 | 0.004 | 0.008 | 0.000 | 0.002 | 0.000 | 0.000 | **0.003** | **0.005** | **0.000** | **0.000** |
| K13652 | 0.003 | 0.005 | 0.005 | 0.006 | 0.003 | 0.004 | 0.004 | 0.004 | **0.002** | **0.003** | **0.007** | **0.007** |
| K13678 | 0.000 | 0.000 | 0.003 | 0.004 | 0.001 | 0.002 | 0.001 | 0.002 | **0.001** | **0.002** | **0.000** | **0.001** |
| K13685 | 0.013 | 0.008 | 0.009 | 0.006 | 0.020 | 0.010 | 0.010 | 0.007 | **0.016** | **0.008** | **0.010** | **0.007** |
| K13735 | 0.003 | 0.006 | 0.000 | 0.001 | 0.001 | 0.002 | 0.000 | 0.000 | **0.003** | **0.004** | **0.000** | **0.000** |
| K13888 | 0.007 | 0.013 | 0.006 | 0.010 | 0.001 | 0.002 | 0.000 | 0.001 | **0.005** | **0.004** | **0.001** | **0.002** |
| K13940 | 0.037 | 0.020 | 0.027 | 0.018 | 0.044 | 0.021 | 0.063 | 0.024 | **0.042** | **0.014** | **0.059** | **0.019** |
| K13963 | 0.012 | 0.008 | 0.011 | 0.008 | 0.017 | 0.008 | 0.014 | 0.008 | **0.013** | **0.007** | **0.018** | **0.009** |
| K13990 | 0.004 | 0.006 | 0.016 | 0.012 | **0.012** | **0.011** | **0.001** | **0.002** | **0.017** | **0.010** | **0.003** | **0.004** |
| K14055 | 0.002 | 0.004 | 0.001 | 0.001 | 0.000 | 0.001 | 0.000 | 0.000 | **0.003** | **0.004** | **0.000** | **0.000** |
| K14058 | 0.007 | 0.009 | 0.007 | 0.012 | 0.000 | 0.001 | 0.000 | 0.000 | **0.004** | **0.004** | **0.000** | **0.001** |
| K14061 | 0.003 | 0.004 | 0.000 | 0.001 | 0.000 | 0.001 | 0.000 | 0.000 | **0.002** | **0.003** | **0.000** | **0.001** |
| K14187 | 0.002 | 0.003 | 0.001 | 0.002 | 0.001 | 0.002 | 0.000 | 0.001 | **0.003** | **0.004** | **0.001** | **0.002** |
| K14534 | 0.019 | 0.011 | 0.012 | 0.008 | 0.031 | 0.019 | 0.025 | 0.016 | **0.023** | **0.011** | **0.016** | **0.010** |
| K14623 | 0.007 | 0.006 | 0.004 | 0.005 | 0.004 | 0.004 | 0.007 | 0.005 | **0.007** | **0.005** | **0.016** | **0.011** |
| K14652 | 0.096 | 0.025 | 0.115 | 0.022 | 0.108 | 0.022 | 0.093 | 0.030 | **0.112** | **0.020** | **0.091** | **0.023** |
| K14744 | 0.002 | 0.004 | 0.000 | 0.001 | 0.000 | 0.001 | 0.000 | 0.000 | **0.003** | **0.004** | **0.000** | **0.000** |

Table S7. Average (± SD) relative abundance (%) of metabolic pathways from predicted metagenomes of samples from vervets and humans consuming non-Western and Western diets. Western humans are from Italy (Schnorr et al. 2014) and the U.S. (Yatsunenko et al. 2012). Non-western humans are from Tanzania (Hadza, Schnorr et al. 2014), Venezuela (Guahibo, Yatsunenko et al. 2012), and Malawi (Yatsunenko et al. 2012). Bold text indicates significant differences between diet treatments (p < 0.05).

|  | Amato et al. vervets | | | | Schnorr et al. humans | | | | Yatsunenko et al. humans | | | |
| --- | --- | --- | --- | --- | --- | --- | --- | --- | --- | --- | --- | --- |
|  | non-Western | | Western | | non-Western | | Western | | non-Western | | Western | |
|  | Avg | SD | Avg | SD | Avg | SD | Avg | SD | Avg | SD | Avg | SD |
| Amino Acid Metabolism | 9.552 | 0.301 | 9.678 | 0.367 | 9.757 | 0.216 | 9.874 | 0.165 | **9.548** | **0.177** | **9.810** | **0.153** |
| Biosynthesis of Other Secondary Metabolites | 0.849 | 0.076 | 0.895 | 0.083 | 0.914 | 0.066 | 0.957 | 0.044 | **0.886** | **0.043** | **0.944** | **0.068** |
| Cancers | 0.113 | 0.020 | 0.120 | 0.022 | 0.098 | 0.015 | 0.090 | 0.016 | **0.108** | **0.017** | **0.095** | **0.014** |
| Carbohydrate Metabolism | 10.329 | 0.605 | 10.075 | 0.398 | **10.381** | **0.380** | **10.861** | **0.468** | **10.219** | **0.280** | **10.961** | **0.394** |
| Cell Growth and Death | 0.535 | 0.044 | 0.573 | 0.058 | 0.514 | 0.036 | 0.495 | 0.043 | **0.534** | **0.039** | **0.503** | **0.032** |
| Circulatory System | 0.012 | 0.026 | 0.014 | 0.021 | 0.000 | 0.000 | 0.000 | 0.000 | **0.001** | **0.003** | **0.000** | **0.001** |
| Digestive System | **0.033** | **0.014** | **0.047** | **0.016** | 0.034 | 0.024 | 0.022 | 0.012 | **0.049** | **0.018** | **0.027** | **0.012** |
| Enzyme Families | 2.101 | 0.156 | 2.092 | 0.199 | 2.224 | 0.084 | 2.201 | 0.067 | **2.213** | **0.068** | **2.162** | **0.071** |
| Excretory System | 0.025 | 0.010 | 0.019 | 0.011 | 0.015 | 0.007 | 0.013 | 0.009 | **0.013** | **0.007** | **0.018** | **0.010** |
| Folding, Sorting and Degradation | 2.419 | 0.133 | 2.404 | 0.161 | 2.419 | 0.113 | 2.356 | 0.090 | **2.506** | **0.100** | **2.378** | **0.075** |
| Genetic Information Processing | 2.675 | 0.114 | 2.606 | 0.175 | **2.749** | **0.077** | **2.645** | **0.097** | **2.720** | **0.091** | **2.610** | **0.093** |
| Glycan Biosynthesis and Metabolism | 1.892 | 0.293 | 2.089 | 0.292 | 1.885 | 0.300 | 1.755 | 0.249 | **2.154** | **0.209** | **1.928** | **0.267** |
| Infectious Diseases | 0.361 | 0.036 | 0.382 | 0.035 | 0.348 | 0.031 | 0.338 | 0.024 | **0.371** | **0.033** | **0.348** | **0.028** |
| Lipid Metabolism | 2.953 | 0.270 | 2.885 | 0.344 | 2.743 | 0.097 | 2.831 | 0.098 | **2.736** | **0.095** | **2.884** | **0.113** |
| Membrane Transport | 13.052 | 0.834 | 12.886 | 1.296 | 12.605 | 1.010 | 13.211 | 0.809 | **12.145** | **0.702** | **12.684** | **0.627** |
| Metabolism | 2.366 | 0.093 | 2.373 | 0.116 | 2.331 | 0.079 | 2.338 | 0.099 | **2.313** | **0.106** | **2.420** | **0.100** |
| Metabolism of Cofactors and Vitamins | 4.096 | 0.251 | 4.100 | 0.299 | 4.286 | 0.167 | 4.283 | 0.112 | **4.410** | **0.140** | **4.273** | **0.149** |
| Metabolism of Other Amino Acids | **1.397** | **0.152** | **1.548** | **0.191** | 1.385 | 0.068 | 1.365 | 0.057 | **1.430** | **0.053** | **1.389** | **0.077** |
| Metabolism of Terpenoids and Polyketides | 1.660 | 0.138 | 1.751 | 0.148 | 1.611 | 0.074 | 1.573 | 0.077 | **1.671** | **0.076** | **1.595** | **0.062** |
| Nervous System | 0.092 | 0.015 | 0.094 | 0.017 | 0.112 | 0.011 | 0.109 | 0.014 | **0.106** | **0.015** | **0.116** | **0.014** |
| Nucleotide Metabolism | 4.014 | 0.171 | 4.013 | 0.417 | 4.068 | 0.193 | 3.960 | 0.143 | **4.214** | **0.183** | **3.919** | **0.130** |
| Replication and Repair | 8.937 | 0.516 | 8.728 | 0.969 | 9.130 | 0.329 | 8.861 | 0.213 | **9.390** | **0.322** | **8.830** | **0.257** |
| Transcription | **2.871** | **0.352** | **2.683** | **0.221** | 3.084 | 0.144 | 3.178 | 0.152 | **2.953** | **0.112** | **3.027** | **0.097** |
| Translation | 5.869 | 0.324 | 5.734 | 0.707 | **5.956** | **0.189** | **5.720** | **0.171** | **6.111** | **0.241** | **5.701** | **0.200** |
| Transport and Catabolism | **0.230** | **0.040** | **0.268** | **0.042** | 0.228 | 0.045 | 0.201 | 0.057 | 0.223 | 0.036 | 0.230 | 0.055 |
| Xenobiotics Biodegradation and Metabolism | 1.712 | 0.437 | 1.920 | 0.805 | 1.521 | 0.111 | 1.598 | 0.126 | **1.517** | **0.089** | **1.614** | **0.102** |

Table S8. Body weights for wild vervets consuming a non-Western diet and for captive vervets before and after consuming a Western diet. Data are shown only for those vervets included in final analyses.

| Individual | Diet | Weight before diet shift | Weight after diet shift |
| --- | --- | --- | --- |
| 07 | non-Western | na | 2.8 |
| 11 | non-Western | na | 2.68 |
| 18 | non-Western | na | 3.99 |
| 20 | non-Western | na | 3.27 |
| 26 | non-Western | na | 6.71 |
| 30 | non-Western | na | 2.94 |
| 52 | non-Western | na | 3.32 |
| 56 | non-Western | na | 2.6 |
| 57 | non-Western | na | 2.32 |
| 60 | non-Western | na | 4.21 |
| 61 | non-Western | na | na |
| 62 | non-Western | na | 2.45 |
| 64 | non-Western | na | 3.96 |
| Average |  | na | 3.44 |
| SD |  | na | 1.21 |
| 1089 | Western | 4.73 | 4.87 |
| 1103 | Western | 5.41 | 5.68 |
| 1104 | Western | 4.70 | 4.28 |
| 1204 | Western | 5.56 | 5.18 |
| 1286 | Western | 4.33 | 4.02 |
| 1388 | Western | 4.37 | 4.23 |
| 1400 | Western | 5.67 | 4.67 |
| 1449 | Western | 5.92 | 5.53 |
| 1159 | Western | 6.48 | 6.71 |
| 1201 | Western | 5.45 | 6.17 |
| 1356 | Western | 8.64 | 9.46 |
| 1430 | Western | 5.96 | 5.93 |
| 1462 | Western | 6.80 | 7.39 |
| Average |  | 5.69 | 5.70 |
| SD |  | 1.17 | 1.51 |

Supplementary Figures

Figure S1. Principle components analysis (PCoA) plot based on weighted UniFrac distances illustrating clustering patterns in gut microbiomes across sampling groups at the OTU level. Vervet outliers have been removed.

Figure S2. Average (± SD) unweighted UniFrac distance between Western and non-Western vervet and human samples. HW = Western human; HNW = non-Western human; VW = Western vervet; VNW = non-Western vervet. Letters represent pairs of sample categories with significantly different UniFrac distances (p < 0.05).


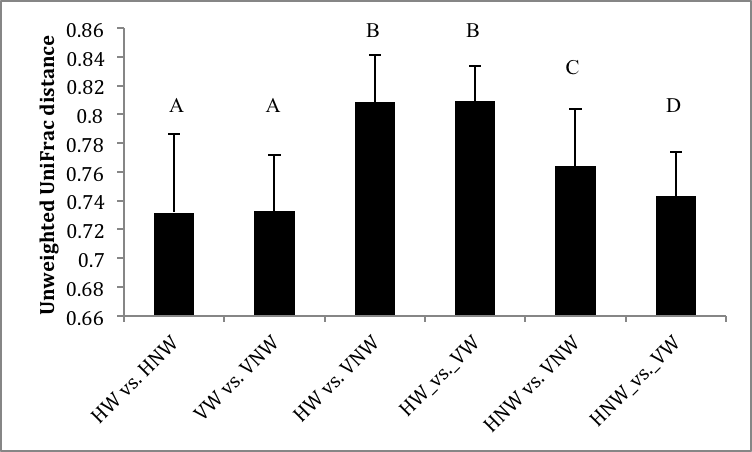


Figure S3. Principle components analysis (PCoA) plot based on unweighted UniFrac distances illustrating clustering patterns in gut microbiomes across sampling groups at the OTU level. Mangabeys (*C. agilis*) from the Central African Republic have been included to illustrate the lack of effect of increased host genetic diversity in primates.

Figure S4. Principle components analysis (PCoA) plot based on unweighted UniFrac distances illustrating clustering patterns in gut microbiomes across sampling groups at the OTU level. TWD vervet outliers have not been removed.

Figure S5. Principle components analysis (PCoA) plot based on weighted UniFrac distances illustrating clustering patterns in gut microbiomes across sampling groups at the OTU level. TWD vervet outliers have not been removed.
